# C-reactive protein/albumin ratio is the most significant inflammatory marker in unresectable pancreatic cancer treated with FOLFIRINOX or gemcitabine plus nab-paclitaxel

**DOI:** 10.1038/s41598-023-34962-7

**Published:** 2023-05-31

**Authors:** Tsuyoshi Shirakawa, Akitaka Makiyama, Mototsugu Shimokawa, Taiga Otsuka, Yudai Shinohara, Futa Koga, Yujiro Ueda, Junichi Nakazawa, Satoshi Otsu, Azusa Komori, Shiho Arima, Masaru Fukahori, Hiroki Taguchi, Takuya Honda, Taro Shibuki, Kenta Nio, Yasushi Ide, Norio Ureshino, Toshihiko Mizuta, Kenji Mitsugi, Koichi Akashi, Eishi Baba

**Affiliations:** 1 Department of Medical Oncology, Fukuoka Wajiro Hospital, 2-2-75 Wajirogaoka, Higashi-Ku, Fukuoka-Shi, Fukuoka, 811-0213 Japan; 2 Department of Internal Medicine, Karatsu Higashi-Matsuura Medical Association Center, 2566-11 Chiyoda-machi, Karatsu-Shi, Saga, 847-0041 Japan; 3 Department of Hematology/Oncology, Japan Community Healthcare Organization Kyushu Hospital, 1-8-1 Kishinoura, Yahatanishi-Ku, Kitakyushu-Shi, Fukuoka, 806-8501 Japan; 4 Cancer Center, Gifu University Hospital, 1-1 Yanagido, Gifu-Shi, Gifu, 501-1194 Japan; 5 Clinical Research Institute, National Kyushu Cancer Center, 3-1-1 Notame, Minami-Ku, Fukuoka-Shi, Fukuoka, 811-1395 Japan; 6 Department of Biostatistics, Yamaguchi University Graduate School of Medicine, 1-1-1 Minamikogushi, Ube-Shi, Yamaguchi, 755-8505 Japan; 7 Department of Medical Oncology, Saga-Ken Medical Center Koseikan, 400 Kase-Machi, Saga-Shi, Saga, 840-8571 Japan; 8 Department of Internal Medicine, Minato Medical Clinic, 3-11-3 Nagahama, Chuo-Ku, Fukuoka-Shi, Fukuoka, 810-0072 Japan; 9 Department of Hepatobiliary and Pancreatology, Saga-Ken Medical Center Koseikan, 400 Kase-Machi, Saga-Shi, Saga, 840-8571 Japan; 10 Department of Hematology and Oncology, Japanese Red Cross Kumamoto Hospital, 2-1-1 Nagamine-Minami, Higashi-Ku, Kumamoto-Shi, Kumamoto, 861-8520 Japan; 11 Department of Medical Oncology, Kagoshima City Hospital, 37-1 Uearata-Cho, Kagoshima-Shi, Kagoshima, 890-8760 Japan; 12 Department of Medical Oncology and Hematology, Oita University Faculty of Medicine, 1-1 Idaigaoka, Hasama-Machi, Yufu-Shi, Oita, 879-5593 Japan; 13 Department of Gastrointestinal Medical Oncology, National Hospital Organization Shikoku Cancer Center, 160 Kou, Minamiumemoto-Machi, Matsuyama-Shi, Ehime 791-0280 Japan; 14 Digestive and Lifestyle Diseases, Kagoshima University Graduate School of Medical and Dental Sciences, 8-35-1 Sakuragaoka, Kagoshima-Shi, Kagoshima, 890-8520 Japan; 15 Division of Gastroenterology, Department of Medicine, Kurume University School of Medicine, 67 Asahi-Machi, Kurume-Shi, Fukuoka, 830-0011 Japan; 16 Kyoto Innovation Center for Next Generation Clinical Trials and iPS Cell Therapy (Ki-CONNECT), Kyoto University Hospital, 54 Kawaharacho, Shogoin, Sakyo-Ku, Kyoto, 606-8507 Japan; 17 Department of Gastroenterology, Saiseikai Sendai Hospital, 2-46 Harada-Cho, Satsumasendai-Shi, Kagoshima, 895-0074 Japan; 18 Department of Gastroenterology, Kagoshima City Hospital, 37-1 Uearata-Cho, Kagoshima-Shi, Kagoshima, 890-8760 Japan; 19 Department of Gastroenterology and Hepatology, Nagasaki University Graduate School of Biomedical Sciences, 1-7-1 Sakamoto, Nagasaki-Shi, Nagasaki, 852-8501 Japan; 20 Department of Internal Medicine, Imari Arita Kyoritsu Hospital, 860 Ninose-Ko, Arita-Cho, Nishi-Matsuura-Gun, Saga, 849-4193 Japan; 21 Department of Hepatobiliary and Pancreatic Oncology, National Cancer Center Hospital East, 6-5-1 Kashiwanohara, Kashiwa-Shi, Chiba, 277-8577 Japan; 22 Department of Medical Oncology, Sasebo Kyosai Hospital, 10-17 Shimanji-Cho, Sasebo-Shi, Nagasaki, 857-8575 Japan; 23 Department of Medical Oncology, Hamanomachi Hospital, 3-3-1 Nagahama, Chuo-Ku, Fukuoka-Shi, Fukuoka, 810-8539 Japan; 24 Department of Internal Medicine, Karatsu Red Cross Hospital, 2430 Watada, Karatsu-Shi, Saga, 847-8588 Japan; 25 Department of Internal Medicine, National Hospital Organization Saga Hospital, 1-20-1 Hinode, Saga-Shi, Saga, 849-8577 Japan; 26 Department of Medical Oncology, Kimitsu Chuo Hospital, 1010 Sakurai, Kisarazu-Shi, Chiba, 292-8535 Japan; 27 Department of Internal Medicine, Fujikawa Hospital, 1-2-6 Matsubara, Saga-Shi, Saga, 840-0831 Japan; 28 Department of Medicine and Biosystemic Science, Kyushu University Graduate School of Medical Sciences, 3-1-1 Maidashi, Higashi‑ku, Fukuoka, 812-8582 Japan; 29 Department of Oncology and Social Medicine, Graduate School of Medical Sciences, Kyushu University, 3-1-1 Maidashi, Higashi‑ku, Fukuoka, 812-8582 Japan

**Keywords:** Cancer, Biomarkers, Gastroenterology, Medical research, Oncology, Risk factors

## Abstract

There are limited absolute biomarkers for determining the prognosis before first- and second-line palliative chemotherapy in unresectable pancreatic cancer (urPC) patients. To find the best prognostic inflammatory marker, we investigated relationships between overall survival (OS) and six inflammatory markers; C-reactive protein/albumin ratio (CAR), neutrophil–lymphocyte ratio (NLR), prognostic nutrition index (PNI), platelet–lymphocyte ratio (PLR), Glasgow prognostic score (GPS), and prognostic index (PI). We examined 255 patients who received gemcitabine + nab-paclitaxel or FOLFIRINOX as first-line chemotherapy and 159 patients who subsequently underwent second-line chemotherapy. First-line patients with lower CAR had better OS compared to those with a higher CAR (hazard ratio 0.57; 95% confidential index 0.42–77; *P* < 0.01). Similarly, lower NLR (*P* = 0.01), higher PNI (*P* = 0.04), lower PLR (*P* = 0.03), GPS score of 0 (*P* < 0.01) and PI score of 0 (*P* < 0.01) were all associated with better OS. CAR demonstrated the best superiority for determining survival prognosis through the use of area under the curve of time-dependent receiver-operating characteristic curves. Furthermore, a lower CAR before second-line therapy exhibited better OS versus higher CAR (*P* < 0.01). Therefore, CAR might be a useful biomarker for predicting urPC patient prognosis in both first- and second-line chemotherapy.

## Introduction

Pancreatic cancer remains one of the cancers with the poorest survival rates. It is the fourth leading cause of cancer-related death in Japan, and the seventh leading cause of cancer-related death worldwide^1,2^. While surgical resection can be a curative treatment for localized pancreatic cancer, just 15% of pancreatic cancer patients are viewed as being candidates for curative resection, as approximately 80% of these patients have unresectable cancers at the time of diagnosis^3^.

Palliative chemotherapy is indicated for unresectable pancreatic cancer patients, with a large increase in recent years in the development of new treatments. Based on the results of prospective randomized phase 3 trials, gemcitabine + nab-paclitaxel (GnP) or FOLFIRINOX (FFX) have now become the standard first-line chemotherapy treatments for metastatic pancreatic cancer^4,5^. The median overall survival (OS) for the GnP and FFX cases has been reported to be 8.5 months and 11.1 months, respectively^4,5^. Second- or later-line chemotherapy, such as nanoliposomal irinotecan with fluorouracil and folic acid, fluorouracil-based, and gemcitabine-based regimens have been shown to have efficacy for pancreatic cancer, with a median OS of 3 to 9 months^6–9^. Moreover, olaparib exhibited an anticancer effect after use as first-line chemotherapy for BRCA-mutated cases^10^. However, the overall prognosis for pancreatic cancer remains poor, with the 5-year survival being less than 10%^11,12^.

Although there should be some reasons as to why pancreatic cancer has such a poor prognosis, at the current time we only have limited absolute biomarkers that can be used to evaluate the prognosis or determine the risk stratification prior to the implementation of the first-line chemotherapy^13,14^. Furthermore, it remains unknown as to what types of patients second-line chemotherapy could potentially bring survival benefits to. As many patients who are at an unresectable stage receive palliative chemotherapy, it is very important to be able to identify both reliable and easily obtainable markers that can be used for better risk stratification of survival and optimal treatment plans. For example, if the prognosis of pancreatic cancer patients could be evaluated more accurately, this would make it possible to assess indications for chemotherapy or choice of regimen, thereby potentially providing more personalized therapy.

Inflammatory markers have possibility for use as prognostic biomarkers. It has been previously reported that there is a correlation between inflammation and malignant tumors^15^. Furthermore, other studies have shown that pretreatment serum inflammatory markers, such as C-reactive protein (CRP), the CRP/albumin ratio (CAR), neutrophil–lymphocyte ratio (NLR), platelet–lymphocyte ratio (PLR), and the modified Glasgow prognostic score (mGPS), are related to the prognosis for various types of cancer^16,17^. However, the prognostic value of these markers has yet to be fully examined, especially in patients with unresectable pancreatic cancer who are classified as candidates for first- and second-line palliative chemotherapy. Therefore, the present study focused on comparing and examining the prognostic significance of different inflammatory markers in patients with unresectable pancreatic cancer who are seen during routine clinical practice.

## Results

### Patient characteristics

**Figure 1:**
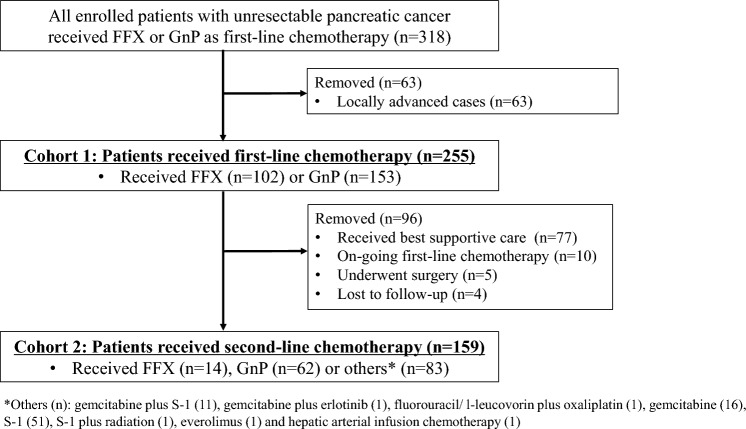
Flow diagram of criteria for patient inclusion or exclusion. *FFX* FOLFIRINOX, *GnP* gemcitabine plus nab-paclitaxel.

From December 2013 to March 2017, FFX and GnP were initially administered to 118 and 200 patients with unresectable pancreatic cancer, respectively. After excluding 63 locally advanced cases, 102 and 153 patients being administered FFX or GnP, respectively, were analyzed in Cohort 1 (Fig. 1). In this group, there were 96 cases that were excluded, with 77 receiving best supportive care, 10 continuing first-line chemotherapy, 5 undergoing surgery^18^ and 4 being lost to follow-up. In Cohort 2, there were 14, 62, and 83 patients who were subsequently treated with the FFX, GnP, or others, respectively.

Table 1 summarizes the baseline characteristics, and Table 2 summarizes the baseline laboratory data. By the end of the follow-up period on July 31, 2018, a total of 197 patients (61.9%) had died. Of these, 195 died from pancreatic cancer, while 2 died from other diseases. The median OS was 11.5 months for the FFX group and 11.1 months for the GnP group^19^. The median follow-up duration was 10.7 months (95% confidential index (95%CI), 9.8–11.5).

**Table 1:** Baseline characteristics.

Characteristics		Cohort 1 (n = 255)	Cohort 2 (n = 159)
Age, years	Median (range)	65 (29–86)	64 (29–87)
Sex, n (%)	Male	158 (62)	92 (58)
ECOG PS, n (%)	0	161 (63)	63 (40)
	1	78 (31)	74 (47)
	≥ 2	16 (6)	21 (13)
	Unknown	–	1 (1)
Malignant history, n (%)	Yes	34 (13)	19 (12)
Malignant family history, n (%)	Yes	75 (29)	48 (30)
Previous tumor resection, n (%)	Yes	44 (17)	20 (13)
Previous biliary drainage, n (%)	Yes	65 (26)	39 (25)
Pancreatic tumor location, n (%)	Head	123 (48)	75 (47)
	Body/tail	132 (52)	84 (53)
Histology, n (%)	Adenocarcinoma	212 (83)	138 (87)
	Others	10 (4)	3 (2)
	Unknown	33 (13)	18 (11)
Site of metastatic disease, n (%)	Liver	154 (60)	100 (63)
	Peritoneum	62 (24)	37 (23)
	Lung	39 (15)	22 (14)
Number of metastatic sites, n (%)	≥ 2	97 (38)	59 (37)
Ascites, n (%)	Yes	56 (22)	29 (18)
First-line chemotherapy, n (%)	FFX	102 (40)	80 (50)
	GnP	153 (60)	79 (50)

*ECOG PS* Eastern Cooperative Oncology Group performance status, *FFX* FOLFIRINOX, *GnP* gemcitabine plus nab-paclitaxel.

**Table 2:** Baseline laboratory data.

Characteristics		Cohort 1 (n = 255)	Cohort 2 (n = 159)
White blood cell count, /μL	Median (range)	6050 (2400–23,650)	4750 (1620–21,910)
Neutrophil count, /μL	Median (range)	3940 (770–20,810)	2900 (610–21,250)
Lymphocyte count, /μL	Median (range)	1360 (230–8640)	1080 (190–3030)
Platelet count, × 10^4^/μL	Median (range)	20.7 (6.7–56.1)	18.3 (2.4–79.8)
Albumin concentration, g/dL	Median (range)	3.8 (2.2–4.8)	3.6 (1.5–4.6)
CRP, mg/dL	Median (range)	0.33 (0.01–17.00)	0.58 (0.01–16.33)
LDH, U/L	Median (range)	180 (74–1320)	208 (84–713)
CEA, ng/mL	Median (range)	5.5 (0.4–626.6)	7.5 (1.0–1379.3)
CA19-9, U/mL	Median (range)	832 (1–6,554,100)	1254 (1–7,200,000)
NLR	Median (range)	3.02 (0.28–36.88)	2.68 (0.37–49.4)
	≥ 5.00, n (%)	46 (18)	34 (21)
PLR	Median (range)	150.9 (16.1–1344.8)	164.5 (35.8–1270.0)
	≥ 150.0, n (%)	128 (50)	89 (56)
PNI	Median (range)	45 (25–66)	42 (19–54)
	< 47, n (%)	157 (62)	133 (84)
CAR	Median (range)	0.084 (0.002–7.182)	0.157 (0.003–6.387)
	≥ 0.540, n (%)	62 (24)	40 (25)
GPS, n (%)	0	146 (57)	80 (50)
	1	62 (24)	48 (30)
	2	47 (18)	31 (20)
PI, n (%)	0	165 (65)	100 (63)
	1	82 (32)	49 (31)
	2	8 (3)	10 (6)

*CRP* C-reactive protein, *LDH* lactate dehydrogenase, *CEA* carcinoembryonic antigen, *CA19-9* carbohydrate antigen 19–9, *NLR* neutrophil-to-lymphocyte ratio, *PLR* platelet-lymphocyte ratio, *PNI* prognostic nutrition index, *CAR* CRP-albumin ratio, *GPS* Glasgow prognostic score, *PI* prognostic index.

### Inflammatory markers in first-line chemotherapy

**Figure 2:**
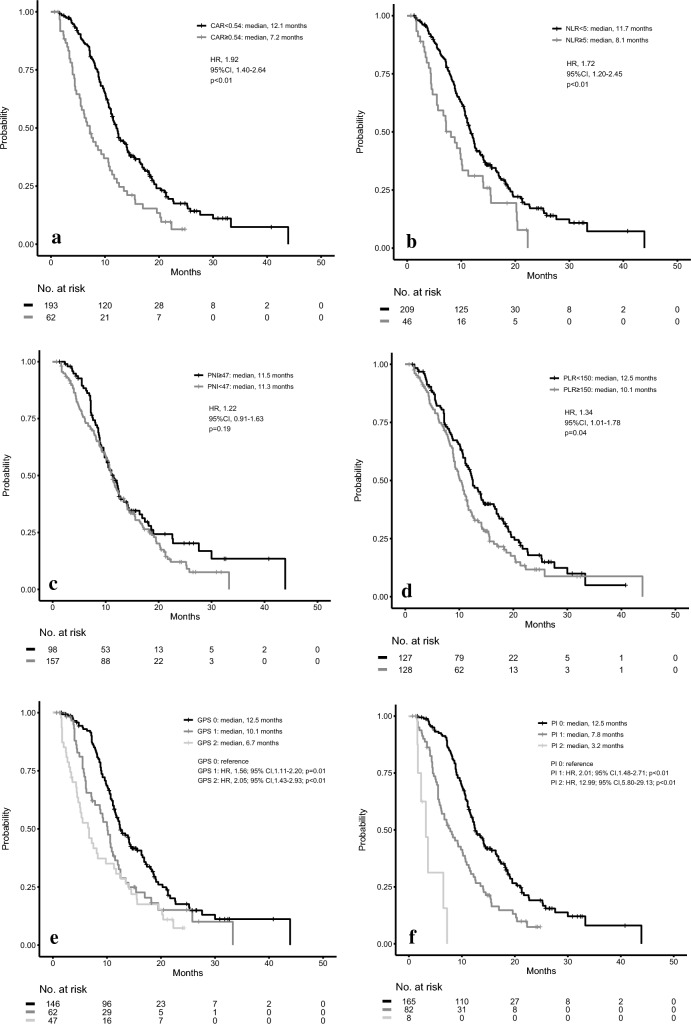
Kaplan–Meier curves for overall survival in first-line chemotherapy. (**a**) CAR < 0.54 group versus CAR ≥ 0.54 group, (**b**) NLR < 5 group versus NLR ≥ 5 group, (**c**) PNI ≥ 47 group versus PNI < 47 group, (**d**) PLR < 150 group versus PLR ≥ 150 group, (**e**) GPS score of 0 versus score of 1 versus score of 2, (**f**) PI score of 0 versus score of 1 versus score of 2. *CAR* C-reactive protein/albumin ratio, *NLR* neutrophil–lymphocyte ratio, *PNI* prognostic nutrition index, *PLR* platelet–lymphocyte ratio, *GPS* modified Glasgow prognostic score, *PI* prognostic index.

Subsequently, we then analyzed the OS of patients after dividing them into two groups based on their respective inflammatory marker values. The median OS of the CAR < 0.54 group was 12.1 months, while it was 7.2 months in the CAR ≥ 0.54 group. There was a statistically significant difference between the groups (hazard ratio (HR) for death, 1.92; 95%CI, 1.40–2.64; *P* < 0.01; Fig. 2a). The median OS of the NLR < 5 group was 11.7 months, while it was 8.1 months in the NLR ≥ 5 group, a difference that was statistically significant (HR for death, 1.72; 95%CI, 1.20–2.45; *P* < 0.01; Fig. 2b). Similarly, the median OS was 11.5 months for the prognostic nutrition index (PNI) ≥ 47 group, while it was 11.3 months for the PNI < 47 group (HR 1.22; 95%CI 0.91–1.63; *P* = 0.19; Fig. 2c). The median OS was 12.5 months for the PLR < 150 group, while it was 10.1 months for the PLR ≥ 150 group (HR 1.34; 95%CI 1.01–1.78; *P* = 0.04; Fig. 2d). In addition, patients with a GPS score of 0 (median OS, 12.5 months) had a significantly better OS as compared to those with a GPS score of 1 (median OS, 10.1 months) and 2 (median OS, 6.7 months), respectively (score of 1 HR, 1.56; 95%CI, 1.11–2.20; *P* = 0.01; score of 2 HR, 2.05; 95%CI, 1.43–2.93; *P* < 0.01; Fig. 2e). As for the prognostic index (PI), patients with a score of 0 (median OS, 12.5 months) had a significantly better OS as compared to those with a score of 1 (median OS, 7.8 months) and 2 (median OS, 3.2 months), respectively (score of 1 HR, 2.01; 95%CI, 1.48–2.71; *P* < 0.01; score of 2 HR, 12.99; 95%CI, 5.80–29.13; *P* < 0.01; Fig. 2f). On multivariate analysis that included Eastern Cooperative Oncology Group performance status (ECOG-PS), carbohydrate antigen 19–9 (CA19-9), CAR, age, and the number of metastases, CAR was significantly associated with OS (adjusted HR 1.66; 95%CI 1.17–2.34; *P* < 0.01). Supplemental Fig. 1a–f presents the progression-free survival (PFS) of patients after dividing them into sets of two groups by six inflammatory markers in first-line chemotherapy. There were significant differences between the groups in all markers except for PLR. Supplemental Fig. 2a–f presents the data for the FFX and GnP subgroups of the 6 inflammatory markers. The Supplemental Table presents the baseline characteristics of the CAR < 0.54 group and CAR ≥ 0.54 group for both the first- and second-line treatments. There were several significant differences in the patient characteristics, such as for the performance status (PS) or laboratory data, and between the CAR < 0.54 and CAR ≥ 0.54 groups for both the first- and second-line treatments. However, there was no significant difference observed between the two groups for the first-line regimens: FFX or GnP. Cox regression analysis for the four continuous variables, CAR, NLR, PLR, and PNI, was performed to compare their significance. In first-line chemotherapy, the HR of CAR was 1.72 (95%CI 1.47–2.01; *P* < 0.01). Similarly, the HRs of NLR, PLR, and PNI were 1.03 (95%CI 0.998–1.06; *P* = 0.07), 1.00 (95%CI 0.999–1.001; *P* = 0.29), and 0.98 (95%CI 0.96–0.999; *P* = 0.04), respectively.

### Inflammatory markers observed during second-line chemotherapy

**Figure 3:**
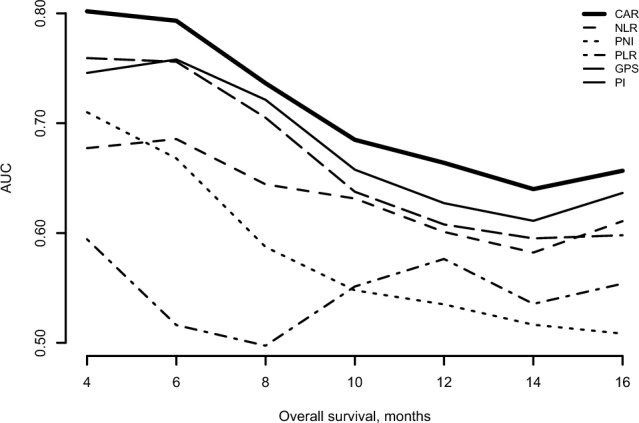
AUC of the time-dependent ROC curve for the prognostic performance evaluation of each inflammatory marker. *AUC* area under the curve, *ROC* receiver-operating characteristic, *CAR* C-reactive protein/albumin ratio, *NLR* neutrophil–lymphocyte ratio, *PNI* prognostic nutrition index, *PLR* platelet–lymphocyte ratio, *GPS* modified Glasgow prognostic score, *PI* prognostic index.

**Figure 4:**
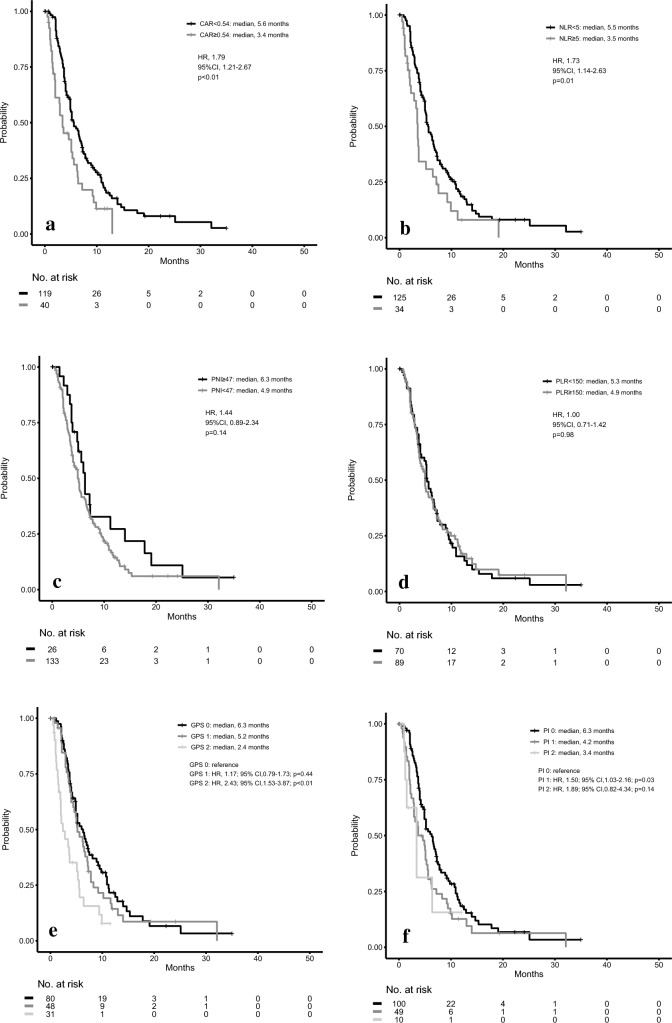
Kaplan–Meier curves for overall survival in the second-line chemotherapy patients. (**a**) CAR < 0.54 group versus CAR ≥ 0.54 group, (**b**) NLR < 5 group versus NLR ≥ 5 group, (**c**) PNI ≥ 47 group versus PNI < 47 group, (**d**) PLR < 150 group versus PLR ≥ 150 group, (**e**) GPS score of 0 versus score of 1 versus score of 2, (**f**) PI score of 0 versus score of 1 versus score of 2. *CAR* C-reactive protein/albumin ratio, *NLR* neutrophil–lymphocyte ratio, *PNI* prognostic nutrition index, *PLR* platelet–lymphocyte ratio, *GPS* modified Glasgow prognostic score, *PI* prognostic index.

In order to determine the most significant inflammatory marker that can be used to predict survival, we calculated the area under the curves (AUCs) for each of the markers to assess the prognosis performance for the first-line chemotherapy. CAR exhibited the highest value with regard to the 4- to 16-month OS (Fig. 3). When we calculated the Akaike’s information criterions (AICs) for the 6 markers used for the sensitivity analysis, the AICs for CAR, NLR, PLR, PNI, GPS, and PI were 1828.355, 1859.623, 1861.325, 1858.183, 1846.128, and 1829.535, respectively. Thus, as it was the most significant inflammatory marker with regard to the OS for the second-line chemotherapy in each patient group, we focused our analysis on CAR. After undergoing first-line chemotherapy, there were 159 patients who received second-line chemotherapy (Fig. 1). Of these, 119 patients were allocated to the CAR < 0.54 group, while there were 40 allocated to the CAR ≥ 0.54 group (Table 1). The median OS for the entire CAR < 0.54 group was 5.6 months, while it was 3.4 months for the entire CAR ≥ 0.54 group. There was a statistically significant difference between these two groups (HR 1.79; 95%CI 1.21–2.67; *P* < 0.01; Fig. 4a). The median OS of the NLR < 5 group was 5.5 months, while it was 3.5 months in the NLR ≥ 5 group, which was statistically significant (HR for death, 1.73; 95%CI, 1.14–2.63; *P* = 0.01; Fig. 4b). The median OS was 6.3 months for the PNI ≥ 47 group, while it was 4.9 months for the PNI < 47 group (HR 1.44; 95%CI 0.89–2.34; *P* = 0.14; Fig. 4c). The median OS was 5.3 months for the PLR < 150 group, while it was 4.9 months for the PLR ≥ 150 group (HR 1.00; 95%CI 0.71–1.42; *P* = 0.98; Fig. 4d). In addition, patients with a GPS score of 0 (median OS, 6.3 months) had a significantly better OS as compared to those with a GPS score of 2 (median OS, 2.4 months; HR, 2.43; 95%CI, 1.53–3.87; *P* < 0.01). However, this was not better than that for the patients with a GPS score of 1 (median OS, 5.2 months; HR, 1.17; 95%CI, 0.79–1.73; *P* = 0.44, Fig. 4e). As for the PI, although patients with a score of 0 (median OS, 6.3 months) had a significantly better OS as compared to those with a score of 1 (median OS, 4.2 months; HR, 1.50; 95%CI, 1.03–2.16; *P* = 0.03), this was not better as compared to those with a score of 2 (median OS, 3.4 months; HR, 1.89; 95%CI, 0.82–4.34; *P* = 0.14, Fig. 4f). Supplemental Fig. 3a–f presents the PFS of patients after dividing them into sets of two groups by six inflammatory markers in second-line chemotherapy. There were no significant differences between the groups in all markers.

In the FFX cases, the median OS for the CAR < 0.54 group was 4.9 months, while it was 4.6 months in the CAR ≥ 0.54 group (HR 2.17; 95%CI 0.56–8.44; *P* = 0.27; Supplemental Fig. 4a). There was a statistically significant difference observed in the GnP cases (CAR < 0.54, 6.7 months; CAR ≥ 0.54, 5.1 months; HR 2.25; 95%CI 1.19–4.25; *P* = 0.01; Supplemental Fig. 4b). In other regimens, the median OS for the CAR < 0.54 group was 5.2 months, whereas it was 2.9 months for the CAR ≥ 0.54 group. There was no significant difference between these two groups (HR 1.64; 95%CI 0.93–2.88; *P* = 0.09; Supplemental Fig. 4c). On multivariate analysis that included Eastern Cooperative Oncology Group performance status (ECOG-PS), carbohydrate antigen 19–9 (CA19-9), CAR, age, and the number of metastases, CAR was significantly associated with OS (adjusted HR 1.95; 95%CI 1.24–3.06; *P* < 0.01). Table 3 shows the distribution of the CAR-high and CAR-low between Cohorts 1 and 2. Cox regression analysis for the four continuous variables, CAR, NLR, PLR, and PNI, was performed to compare their significance. In second-line chemotherapy, the HR of CAR was 1.37 (95%CI 1.17–1.61; *P* < 0.01). Similarly, the HRs of NLR, PLR, and PNI were 1.02 (95%CI 0.99–1.06; *P* = 0.16), 1.00 (95%CI 0.999–1.001; *P* = 0.54), and 0.94 (95%CI 0.91–0.98; *P* < 0.01), respectively.

**Table 3:** Distribution of CAR-high and CAR-low between the Cohorts 1 and 2.

		Cohort 2			
		CAR-high	CAR-low	N/A	Total
Cohort 1	CAR-high	19	18	25	62
	CAR-low	21	101	71	193
	Total	40	119	96	255

*CAR* CRP-albumin ratio, *N/A* not applicable.

## Discussion

We investigated the adequacy of the prognosis prediction by measuring each of the six inflammatory markers at the start of both the first- and second-line chemotherapy treatments for Japanese pancreatic cancer patients who were undergoing palliative chemotherapy. Recently, NLR, CAR, and PNI have been evaluated for use as prognostic markers in pancreatic cancer^20–24^. These markers were reported to be useful for predicting survival among various diseases or therapeutic stages. For example, GPS was shown to be an independent marker of poor prognosis in hepatocellular carcinoma^16^, CAR was associated with the OS in hepatocellular carcinoma^25^, and NLR was an independent indicator of poor prognosis in patients with pancreatic cancer at the time of diagnosis^17,21^. Although previous reports have suggested that inflammatory markers might be useful as a predictor of the prognosis at a limited stage, our outcomes suggest that CAR could be the most significant biomarker, as it appears to be able to predict the survival of unresectable pancreatic cancer patients at the start of both the first- and second-line chemotherapy treatments. This study was carried out as a post-hoc analysis of the NAPOLEON study^19^, with the results showing that OS in the FFX and GnP groups was about 11 months. Our current study appears to be a proper analysis of valid clinical cases that meet the criteria for chemotherapy, with the data generally corresponding with the results of previous pivotal clinical trials^4,5^ that reported a median follow-up duration of 10.7 months.

We initially analyzed the prognosis of unresectable pancreatic cancer patients who underwent the standard first-line chemotherapy of FFX or GnP, by examining six inflammatory markers. While there was significant stratification of the OS observed for every inflammatory marker in all of the patients administered FFX, this was only seen for CAR in patients with GnP, when the analysis was carried out in more than 10 cases in each study arm. To definitively evaluate this, further analyses for the other markers in GnP cases will need to be undertaken, as our data showed there was a tendency of stratification in the OS at each of the cut-offs. The hazard ratio of CAR was the highest for the stratified analysis of the two arms. Furthermore, the AUC of the time-dependent ROC curve for the prognostic performance evaluation done during the first-line treatment showed that the best predictive value was for CAR. Thus, we speculated that CAR might be the best marker for predicting the survival at the initiation of first-line chemotherapy. As the median OS for the first-line chemotherapy in patients with unresectable pancreatic cancer has been reported to be 11.1 months^5^, and as the median OS for the CAR < 0.54 group was 12.1 months in our current study, this suggests that CAR might be the best tool for predicting the OS prior to the induction of chemotherapy. However, since the median OS for the CAR ≥ 0.54 group was 7.2 months, which was longer than the 6.8 months for patients being treated with gemcitabine^4^, the indication for chemotherapy should not be excluded for the CAR < 0.54 group. Although we also demonstrated there was a statistical significance for the PI, there were only a small number of patients with a score of 2 that were evaluated, thus making it hard to generalize the overall results. However, it is possible that GPS might also be useful in first-line cases.

In all cases that received second-line chemotherapy, OS was significantly longer in the CAR < 0.54 group than in CAR ≥ 0.54 group (median OS; 5.6 vs. 3.6 months). They were within the median OS of second- or later-line chemotherapy (3 to 9 months^6–9^), although the cohort 2 group included 50% of the gemcitabine-refractory patients. As both monotherapy and combination-therapy were included in the second-line regimens used in our study, this suggests that CAR might be a useful prognostication tool reflecting these initial cases. However, we cannot definitively state that this second-line chemotherapy was not effective for the CAR ≥ 0.54 group, as the OS of the fluorouracil group was 3.3 months^7^. There was no statistical significance found for the PNI, PLR, GPS, and PI. Significance was also shown for the NLR with the HR equal to that found for CAR. However, we considered CAR to be superior to NLR based on the HR of CAR, along with the fact that the AIC and AUC were higher for the first-line treatments. In addition, comparing the prognostic significance of CAR with that of other inflammatory parameters using their median values is important. The cut-off values of PLR and PNI in this study were very close to the median values without prognostic significance, so additional analyses were performed for CAR and NLR. Only CAR showed prognostic significance in both first and second lines using median values (Supplemental Fig. 5a–d). The present study included 255 patients, more than the previous study^20^, so the CAR cut-off of 0.54 could have enriched more aggressive disease in the CAR high group in first-line chemotherapy. However, the present study was retrospective, and we are now confirming the repeatability of the CAR cut-off of 0.54 in a prospective study with a different cohort.

CAR is calculated based on the serum concentrations of CRP and albumin. The inflammatory mediator, interleukin-6 (IL-6), is a remarkable inflammatory cytokine that is secreted by the immune cells or tumor cells that regulate the levels of CRP^26^, in addition to mediating the chemoresistance^27^. Also, it has been reported that IL-6 and CRP exhibit a significant correlation with their elevation related to poor survival^20,28–30^. Moreover, the IL-6 level reflects the state of the tumor^31^. Thus, a higher CRP is considered to be a prognostic factor, with previous results showing associations with various types of cancers in patients^32^. In gastroenterological cancers, it has been reported that there is a strong relationship between inflammation and low albumin levels^33^. Inflammatory cytokines suppress albumin synthesis at the hepatocytes^34,35^, with changes in the serum albumin concentration influencing the pharmacokinetics and hematologic toxicity in chemotherapy^36^. Low albumin causes a decrease in the binding-rate of anti-cancer drugs^37^, and increases in adverse events^38^. Therefore, as low albumin levels are known to weaken the effect of the chemotherapy, pretreatment albumin levels can be used to predict OS^39–41^. The reasons why CAR had greater prognostic power than other inflammatory markers were further considered. GPS and PI had the disadvantage of being categorical variables that failed to accurately reflect each patient’s disease condition. PLR and PNI were proven to be useful markers mainly for perioperative pancreatic cancer patients^23,24,46^, different from our inclusion criteria. There might be a large difference between previous studies and the present study because the present study excluded patients with locally advanced pancreatic cancer^20,21^. Therefore, CAR could show better prognostic significance than NLR both in first and second lines, on both Cox regression analysis and stratified analysis using their median values. Taken together, this suggests that CAR might be regarded as a prognostic marker. Doctors and patients could better prepare for future clinical courses if it is known that a patient’s prognosis would be predicted to be poor. In our present study, although it appeared that CAR might be the most significant inflammatory marker that can be used for unresectable pancreatic cancer cases, we will need to prospectively collect and analyze further data, as the other five markers also showed effectiveness in predicting the OS.

There were some limitations to the present study. First, as this study was a nonrandomized and retrospective study, our results could have been affected by selection bias. Moreover, we also need to consider differences in the patient characteristics that are present between the CAR-high and the CAR-low groups. Second, as our study included cases using FFX or GnP as first-line chemotherapy, we did not analyze the use of other regimens as first-line treatments. To overcome these limitations, we are now planning to undertake other prospective and retrospective studies with different cohorts consisting of all regimens in order to confirm the repeatability. Third, the ethnicity and location of the enrolled patients were limited given the present study’s focus on Japanese patients. Despite this, we were able to find common points between the current and previously reported studies^20,22^. Thus, these results suggest that CAR might be a universal marker. In addition, the present study only included a small number of patients who were diagnosed with malignancy based on imaging studies rather than by histological or cytological diagnoses. Moreover, there tended to be some patients evaluated during routine clinical practices who had no other choice except to undergo systemic chemotherapy in the absence of any histological evidence due to a variety of reasons, such as for example, patients who were diagnosed based on emergency or anatomically difficult positions. Thus, in order to build stronger evidence to support the findings of the present study, further studies are warranted.

In conclusion, CAR might be a significant and easy-to-use biomarker that can be utilized for accurate prediction of the prognosis in Japanese patients with unresectable pancreatic cancer who are undergoing first- and second-line chemotherapy. This information could further help clinicians to select appropriate therapeutic strategies for the individualized management of these patients.

## Methods

### Study design

This analysis was performed as a post-hoc analysis of the multicenter retrospective study of GnP or FFX in patients with unresectable pancreatic cancer (NAPOLEON study) that was conducted by specialists from 14 centers specializing in medical oncology and gastroenterology in Japan. The NAPOLEON study was approved by the Institutional Review Boards of each participating institution. We retrospectively reviewed the medical records of consecutive patients with unresectable and recurrent pancreatic cancer who were started on FFX or GnP as a first-line chemotherapy. The main results of the NAPOLEON study have been previously reported^19^.

From December 2013 to March 2017, 318 patients with advanced or recurrent pancreatic cancer received GnP or FFX as first-line chemotherapy. After excluding 63 locally advanced cases, 255 patients who received first-line chemotherapy were analyzed using the data from before the start of the first-line treatment. Subsequently, 159 patients who underwent second-line chemotherapy were then analyzed using the data obtained from just before the start of the second-line treatment (Fig. 1).

### Treatment methods

GnP was administered as follows: nab-paclitaxel (125 mg/m^2^), 30-min intravenous infusion; 30-min gemcitabine (1000 mg/m^2^), intravenous infusion on days 1, 8, and 15 every 4 weeks^4^. The FFX group was comprised of patients who received both the original and modified regimens. Original-FFX was sequentially administered as follows: oxaliplatin (85 mg/m^2^), 2-h intravenous infusion; l-leucovorin (200 mg/m^2^), 2-h intravenous infusion; intravenous infusion of irinotecan (150–180 mg/m^2^), 30 min later and over a 90-min period; fluorouracil (400 mg/m^2^), an intravenous bolus; and continuous intravenous fluorouracil infusion (2400 mg/m^2^) for 46 h every 2 weeks^5^. Modified-FFX was sequentially administered as follows: oxaliplatin (85 mg/m^2^), 2-h intravenous infusion; l-leucovorin (200 mg/m^2^), 2-h intravenous infusion; irinotecan (150 mg/m^2^), intravenous infusion 30 min later over a 90-min period; and continuous intravenous fluorouracil infusion (2400 mg/m^2^) for 46 h every 2 weeks^42^.

### Definition of inflammatory markers

CAR was calculated as follows: serum CRP level (mg/dL) divided by the serum albumin level (g/dL). Similarly, NLR and PLR were calculated as follows: neutrophil count (/μL) divided by the lymphocyte count (/μL), platelet count (/μL) divided by the lymphocyte count (/μL), respectively. We calculated the PNI using the following formula: 10 × serum albumin level (g/dL) + 0.005 × lymphocyte count (/μL). The score of the GPS was defined as follows: serum CRP level ≤ 1.0 (mg/dL) and serum albumin level ≥ 3.5 (g/dL), score of 0; CRP > 1.0 (mg/dL) or albumin < 3.5 (g/dL), score of 1; CRP > 1.0 (mg/dL) and albumin < 3.5 (g/dL), score of 2. In addition, the score of the PI was defined as follows: serum CRP level ≤ 1.0 (mg/dL) and white blood cell count (WBC) ≤ 11,000/μL, score of 0; CRP > 1.0 (mg/dL) and WBC ≤ 11,000 (/μL), score of 1; CRP ≤ 1.0 (mg/dL) and WBC > 11,000 (/μL), score of 1; CRP > 1.0 (mg/dL) and WBC > 11,000 (/μL), score of 2.

### Evaluations and statistical analysis

Patient characteristics evaluated at the start of first- and second-line chemotherapy included age, sex, ECOG performance status, past history, primary tumor site, disease status, metastatic site, treatment regimen at the first-line chemotherapy, lactate dehydrogenase (LDH), carcinoembryonic antigen (CEA), CA19-9 and inflammatory factors. These were all analyzed in order to investigate the correlation with the prognosis by Cox regression model. We used the multiple imputation method^43^ to impute missing data for white blood cell count, neutrophil count, lymphocyte count, platelet count, albumin concentration, and C-reactive protein concentration. The cut-off values for CAR, NLR, PLR, and PNI were adopted as 0.54, 5, 150, and 47, respectively, based on previous studies^20,44–47^. Subsequently, patients were then divided into two groups (low or high) according to each of the inflammatory markers. The compatibility of inflammatory markers was examined through the use of the AUC of the time-dependent ROC curve^48^. These results were then used to evaluate the prognostic performance for each of the inflammatory markers. We also used the AIC for the sensitivity analysis. This was a mathematical method that is used for evaluating how well a model fits the data it was generated from, with the best-fit model defined as that with the lowest value for the AIC^49^.

OS was calculated from the date of administration of the first-line or second-line chemotherapy to the date of death from any cause or was censored at the final follow-up examination. PFS was calculated from the date of first-line or second-line chemotherapy administration to the date of progression or death from any cause, whichever occurred first or was censored at the final follow-up examination.

Survival was estimated using the Kaplan–Meier method, with comparisons of the probability of survival performed using the log-rank test and the Cox proportional hazards model. HRs are expressed with 95%CIs. Factors showing differences with *P* values of < 0.05 were considered statistically significant. Prognostic factors judged to be clinically meaningful and those with *P* values of < 0.05 were selected. Data were collected by clinicians with expertise in clinical research under the supervision of the statistician and then centrally managed. This study was conducted with the approval of the Institutional Review Board of each participating institution, and in accordance with the provisions of the Declaration of Helsinki. Statistical analyses were performed using R version 4.2.0 (R Foundation for Statistical Computing, Vienna, Austria).

### Ethics approval and consent to participate

This study was conducted in accordance with the ethical guideline of the Declaration of Helsinki and was centrally approved by the Institutional review board of Saga Medical Center Koseikan (study ID 17-09-01–02), and also approved by the Institutional Review Boards or Ethics Committee of following institutions: Imari Arita Kyoritsu Hospital, Japanese Red Cross Kumamoto Hospital, Kagoshima City Hospital, Oita University Hospital, Kagoshima University Hospital, Kurume University Hospital, Japan Community Healthcare Organization Kyushu Hospital, Saiseikai Sendai Hospital, Nagasaki University Hospital, Hamanomachi Hospital, Sasebo Kyosai Hospital, Karatsu Red Cross Hospital and Fukuoka Wajiro Hospital prior to the study. Because this study was a retrospective observational study carried out in Japan, informed consent was obtained using the opt-in/opt-out approach according to each participating institution’s policy.

### Supplementary Information

Supplementary Information.

## Data Availability

All data generated or analyzed in this study are stored in a secured research database. Although they are not publicly available, they are available through the corresponding author upon reasonable request.

## Code availability

We used R ver. 4.2.0 (R Foundation for Statistical Computing, Vienna, Austria) for statistical analyses.
