# Author Correction: C-reactive protein/albumin ratio is the most significant inflammatory marker in unresectable pancreatic cancer treated with FOLFIRINOX or gemcitabine plus nab-paclitaxel

**DOI:** 10.1038/s41598-024-55673-7

**Published:** 2024-03-04

**Authors:** Tsuyoshi Shirakawa, Akitaka Makiyama, Mototsugu Shimokawa, Taiga Otsuka, Yudai Shinohara, Futa Koga, Yujiro Ueda, Junichi Nakazawa, Satoshi Otsu, Azusa Komori, Shiho Arima, Masaru Fukahori, Hiroki Taguchi, Takuya Honda, Taro Shibuki, Kenta Nio, Yasushi Ide, Norio Ureshino, Toshihiko Mizuta, Kenji Mitsugi, Koichi Akashi, Eishi Baba

**Affiliations:** 1 Department of Medical Oncology, Fukuoka Wajiro Hospital, 2-2-75 Wajirogaoka, Higashi-Ku, Fukuoka-Shi, Fukuoka, 811-0213 Japan; 2 Department of Internal Medicine, Karatsu Higashi-Matsuura Medical Association Center, 2566-11 Chiyoda-machi, Karatsu-Shi, Saga, 847-0041 Japan; 3 Department of Hematology/Oncology, Japan Community Healthcare Organization Kyushu Hospital, 1-8-1 Kishinoura, Yahatanishi-Ku, Kitakyushu-Shi, Fukuoka, 806-8501 Japan; 4 Cancer Center, Gifu University Hospital, 1-1 Yanagido, Gifu-Shi, Gifu, 501-1194 Japan; 5 Clinical Research Institute, National Kyushu Cancer Center, 3-1-1 Notame, Minami-Ku, Fukuoka-Shi, Fukuoka, 811-1395 Japan; 6 Department of Biostatistics, Yamaguchi University Graduate School of Medicine, 1-1-1 Minamikogushi, Ube-Shi, Yamaguchi, 755-8505 Japan; 7 Department of Medical Oncology, Saga-Ken Medical Center Koseikan, 400 Kase-Machi, Saga-Shi, Saga, 840-8571 Japan; 8 Department of Internal Medicine, Minato Medical Clinic, 3-11-3 Nagahama, Chuo-Ku, Fukuoka-Shi, Fukuoka, 810-0072 Japan; 9 Department of Hepatobiliary and Pancreatology, Saga-Ken Medical Center Koseikan, 400 Kase-Machi, Saga-Shi, Saga, 840-8571 Japan; 10 Department of Hematology and Oncology, Japanese Red Cross Kumamoto Hospital, 2-1-1 Nagamine-Minami, Higashi-Ku, Kumamoto-Shi, Kumamoto, 861-8520 Japan; 11 Department of Medical Oncology, Kagoshima City Hospital, 37-1 Uearata-Cho, Kagoshima-Shi, Kagoshima, 890-8760 Japan; 12 Department of Medical Oncology and Hematology, Oita University Faculty of Medicine, 1-1 Idaigaoka, Hasama-Machi, Yufu-Shi, Oita, 879-5593 Japan; 13 Department of Gastrointestinal Medical Oncology, National Hospital Organization Shikoku Cancer Center, 160 Kou, Minamiumemoto-Machi, Matsuyama-Shi, Ehime 791-0280 Japan; 14 Digestive and Lifestyle Diseases, Kagoshima University Graduate School of Medical and Dental Sciences, 8-35-1 Sakuragaoka, Kagoshima-Shi, Kagoshima, 890-8520 Japan; 15 Division of Gastroenterology, Department of Medicine, Kurume University School of Medicine, 67 Asahi-Machi, Kurume-Shi, Fukuoka, 830-0011 Japan; 16 Kyoto Innovation Center for Next Generation Clinical Trials and iPS Cell Therapy (Ki-CONNECT), Kyoto University Hospital, 54 Kawaharacho, Shogoin, Sakyo-Ku, Kyoto, 606-8507 Japan; 17 Department of Gastroenterology, Saiseikai Sendai Hospital, 2-46 Harada-Cho, Satsumasendai-Shi, Kagoshima, 895-0074 Japan; 18 Department of Gastroenterology, Kagoshima City Hospital, 37-1 Uearata-Cho, Kagoshima-Shi, Kagoshima, 890-8760 Japan; 19 Department of Gastroenterology and Hepatology, Nagasaki University Graduate School of Biomedical Sciences, 1-7-1 Sakamoto, Nagasaki-Shi, Nagasaki, 852-8501 Japan; 20 Department of Internal Medicine, Imari Arita Kyoritsu Hospital, 860 Ninose-Ko, Arita-Cho, Nishi-Matsuura-Gun, Saga, 849-4193 Japan; 21 Department of Hepatobiliary and Pancreatic Oncology, National Cancer Center Hospital East, 6-5-1 Kashiwanohara, Kashiwa-Shi, Chiba, 277-8577 Japan; 22 Department of Medical Oncology, Sasebo Kyosai Hospital, 10-17 Shimanji-Cho, Sasebo-Shi, Nagasaki, 857-8575 Japan; 23 Department of Medical Oncology, Hamanomachi Hospital, 3-3-1 Nagahama, Chuo-Ku, Fukuoka-Shi, Fukuoka, 810-8539 Japan; 24 Department of Internal Medicine, Karatsu Red Cross Hospital, 2430 Watada, Karatsu-Shi, Saga, 847-8588 Japan; 25 Department of Internal Medicine, National Hospital Organization Saga Hospital, 1-20-1 Hinode, Saga-Shi, Saga, 849-8577 Japan; 26 Department of Medical Oncology, Kimitsu Chuo Hospital, 1010 Sakurai, Kisarazu-Shi, Chiba, 292-8535 Japan; 27 Department of Internal Medicine, Fujikawa Hospital, 1-2-6 Matsubara, Saga-Shi, Saga, 840-0831 Japan; 28 Department of Medicine and Biosystemic Science, Kyushu University Graduate School of Medical Sciences, 3-1-1 Maidashi, Higashi‑ku, Fukuoka, 812-8582 Japan; 29 Department of Oncology and Social Medicine, Graduate School of Medical Sciences, Kyushu University, 3-1-1 Maidashi, Higashi‑ku, Fukuoka, 812-8582 Japan

Correction to: *Scientific Reports* 10.1038/s41598-023-34962-7, published online 31 May 2023

The original version of this Article contained an error in the Supplementary Information File, where the Kaplan–Meier curves and numbers at risk were incorrect.

The original Supplementary Information file is provided below.

The original Article has been corrected.

### Supplementary Information

Supplementary Information.

