# Supplementary material for: C-reactive protein/albumin ratio is the most significant inflammatory marker in unresectable pancreatic cancer treated with FOLFIRINOX or gemcitabine plus nab-paclitaxel
Source: Sci Rep. 2023 May 31;13:8815. doi: 10.1038/s41598-023-34962-7 (PMC10232544; doi:10.1038/s41598-023-34962-7)
Supplement: Supplementary file 1 — Supplementary Information. [file 41598_2023_34962_MOESM1_ESM.pdf]

**Supplemental Figure legends**

**Supplemental Figure 1.**

Kaplan-Meier curves for progression-free survival in the first-line chemotherapy patients. (a) CAR < 0.54 group versus CAR ≥ 0.54 group, (b) NLR < 5 group versus NLR ≥ 5 group, (c) PNI ≥ 47 group versus PNI < 47 group, (d) PLR < 150 group versus PLR ≥ 150 group, (e) GPS score of 0 versus score of 1 versus score of 2, (f) PI score of 0 versus score of 1 versus score of 2.

*CAR* C-reactive protein/albumin ratio, *NLR* neutrophil–lymphocyte ratio, *PNI* prognostic nutrition index, *PLR* platelet–lymphocyte ratio, *GPS* modified Glasgow prognostic score, *PI* prognostic index

**Supplemental Figure 2.**

Kaplan-Meier curves for overall survival for the FFX or GnP cases in first-line chemotherapy patients. CAR < 0.54 group versus CAR ≥ 0.54 group in the FFX (a) and GnP (b) cases, NLR < 5 group versus NLR ≥ 5 group in the FFX (c) and GnP (d) cases, PNI ≥ 47 group versus PNI < 47 group in the FFX (e) and GnP (f) cases, PLR < 150 group versus PLR ≥ 150 group in the FFX (g) and GnP (h) cases, GPS score of 0 versus score of 1 versus score of 2 in FFX (i) and GnP (j) cases, PI score of 0 versus score of 1 versus score of 2 in the FFX (k) and GnP (l) cases.

*FFX* FOLFIRINOX*, GnP* gemcitabine plus nab-paclitaxel, *CAR* C-reactive protein/albumin ratio, *NLR* neutrophil–lymphocyte ratio, *PNI* prognostic nutrition index, *PLR* platelet–lymphocyte ratio, *GPS* modified Glasgow prognostic score, *PI* prognostic index

**Supplemental Figure 3.**

Kaplan-Meier curves for progression-free survival in the second-line chemotherapy patients. (a) CAR < 0.54 group versus CAR ≥ 0.54 group, (b) NLR < 5 group versus NLR ≥ 5 group, (c) PNI ≥ 47 group versus PNI < 47 group, (d) PLR < 150 group versus PLR ≥ 150 group, (e) GPS score of 0 versus score

of 1 versus score of 2, (f) PI score of 0 versus score of 1 versus score of 2.

*CAR* C-reactive protein/albumin ratio, *NLR* neutrophil–lymphocyte ratio, *PNI* prognostic nutrition index, *PLR* platelet–lymphocyte ratio, *GPS* modified Glasgow prognostic score, *PI* prognostic index

**Supplemental Figure 4.**

Kaplan-Meier curves for overall survival for the FFX or GnP cases in second-line chemotherapy patients. CAR < 0.54 group versus CAR ≥ 0.54 group in the FFX (a), GnP (b) and other (c) cases.

*FFX* FOLFIRINOX*,* *GnP* gemcitabine plus nab-paclitaxel, *CAR* C-reactive protein/albumin ratio

**Supplemental Figure 5.**

Kaplan-Meier curves for overall survival in the first- and second-line chemotherapy patients. (a) CAR < 0.084 group versus CAR ≥ 0.084 group in first-line, (b) NLR < 3.02 group versus NLR ≥ 3.02 group in first-line, (c) CAR < 0.157 group versus CAR ≥ 0.157 group in second-line, (d) NLR < 2.68 group versus NLR ≥ 2.68 group in second-line.

*CAR* C-reactive protein/albumin ratio, *NLR* neutrophil–lymphocyte ratio

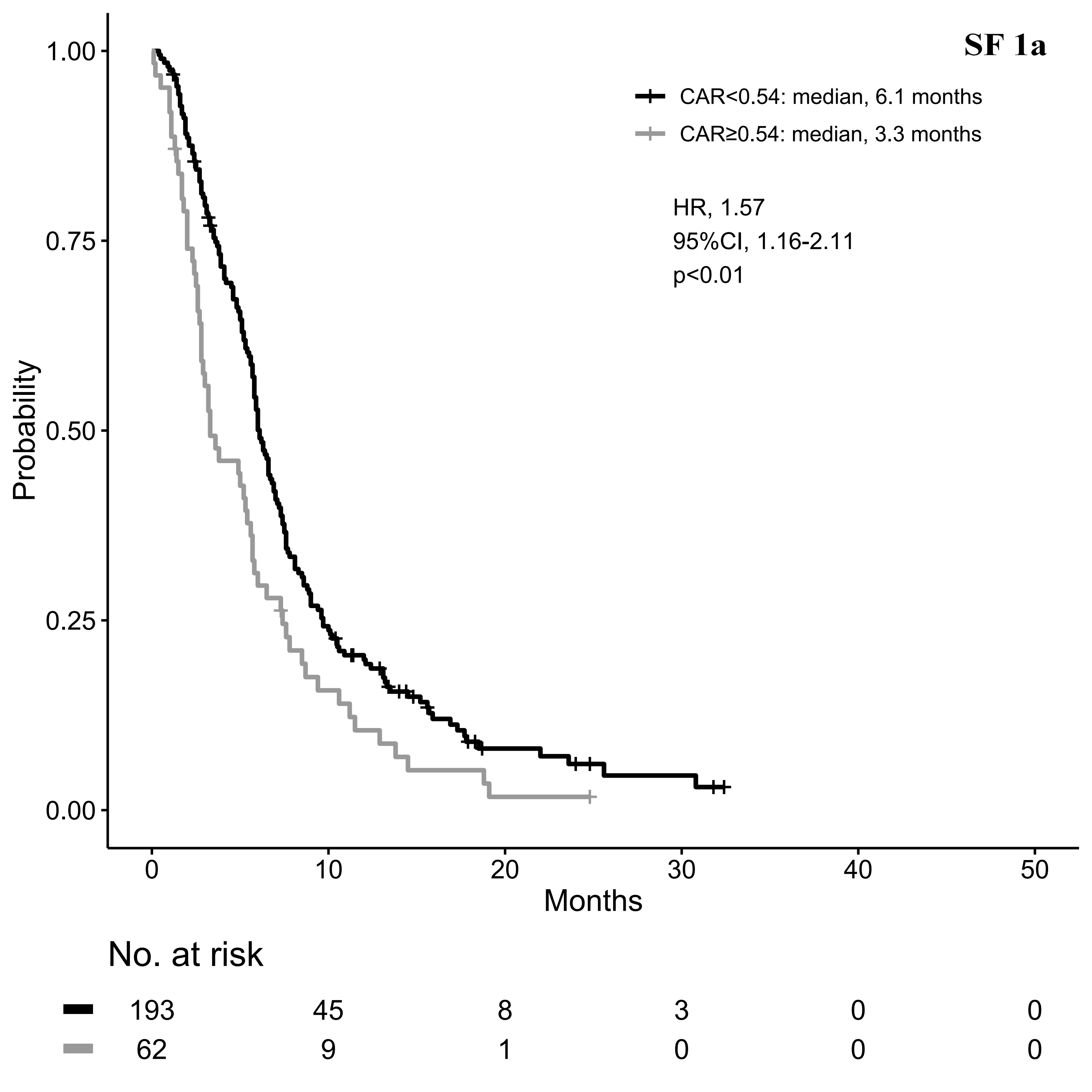

SF 1a
CAR<0.54: median, 6.1 months
CAR≥0.54: median, 3.3 months
HR, 1.57
95%CI, 1.16-2.11
p<0.01
Probability
1.00
0.75
0.50
0.25
0.00
0
10
20
30
40
50
Months
No. at risk
193
45
8
3
0
0
62
9
1
0
0
0

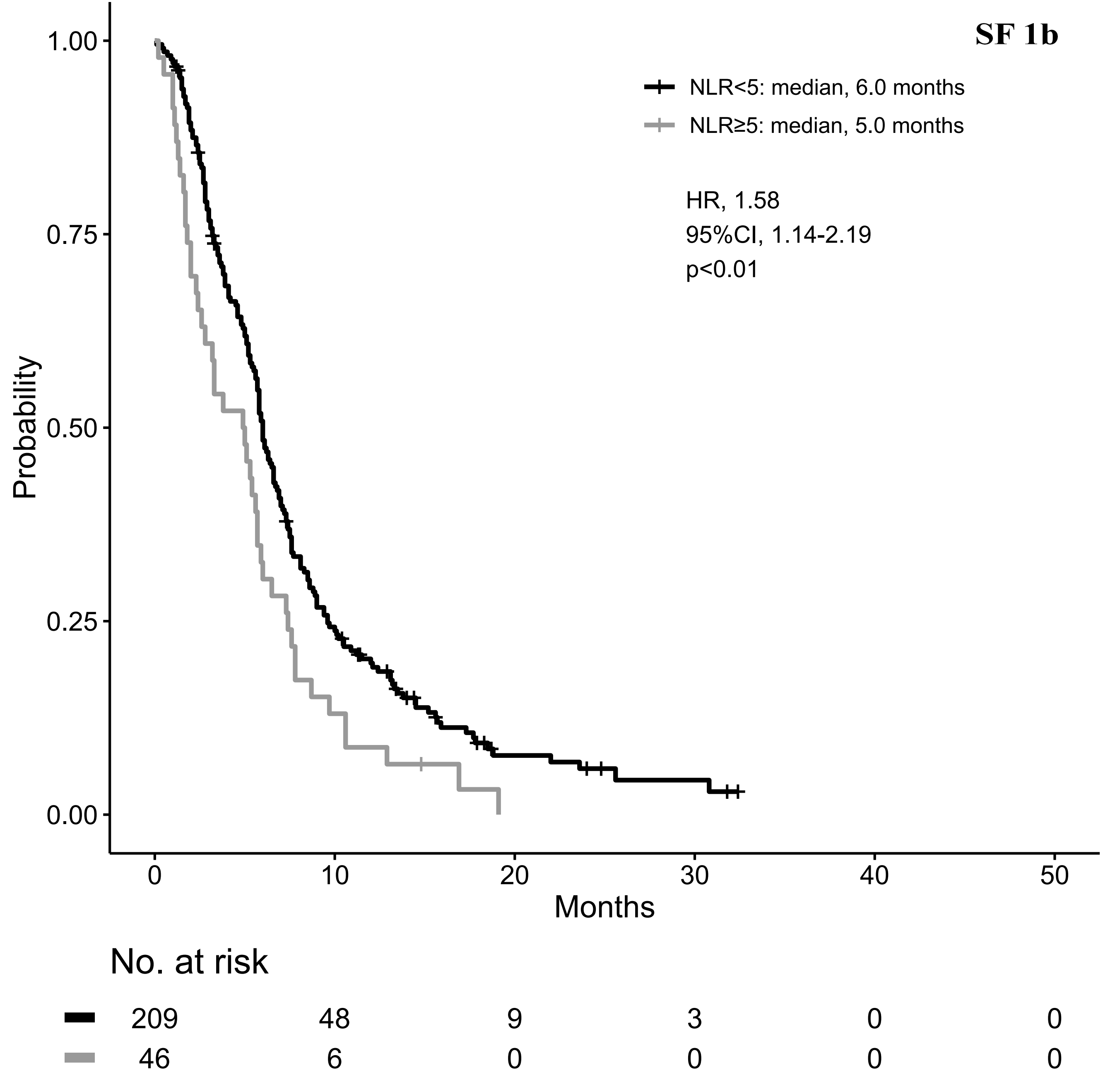
SF 1b
NLR<5: median, 6.0 months
NLR≥5: median, 5.0 months
HR, 1.58
95%CI, 1.14-2.19
p<0.01
Probability
1.00
0.75
0.50
0.25
0.00
0
10
20
30
40
50
Months
No. at risk
209
48
9
3
0
0
46
6
0
0
0
0

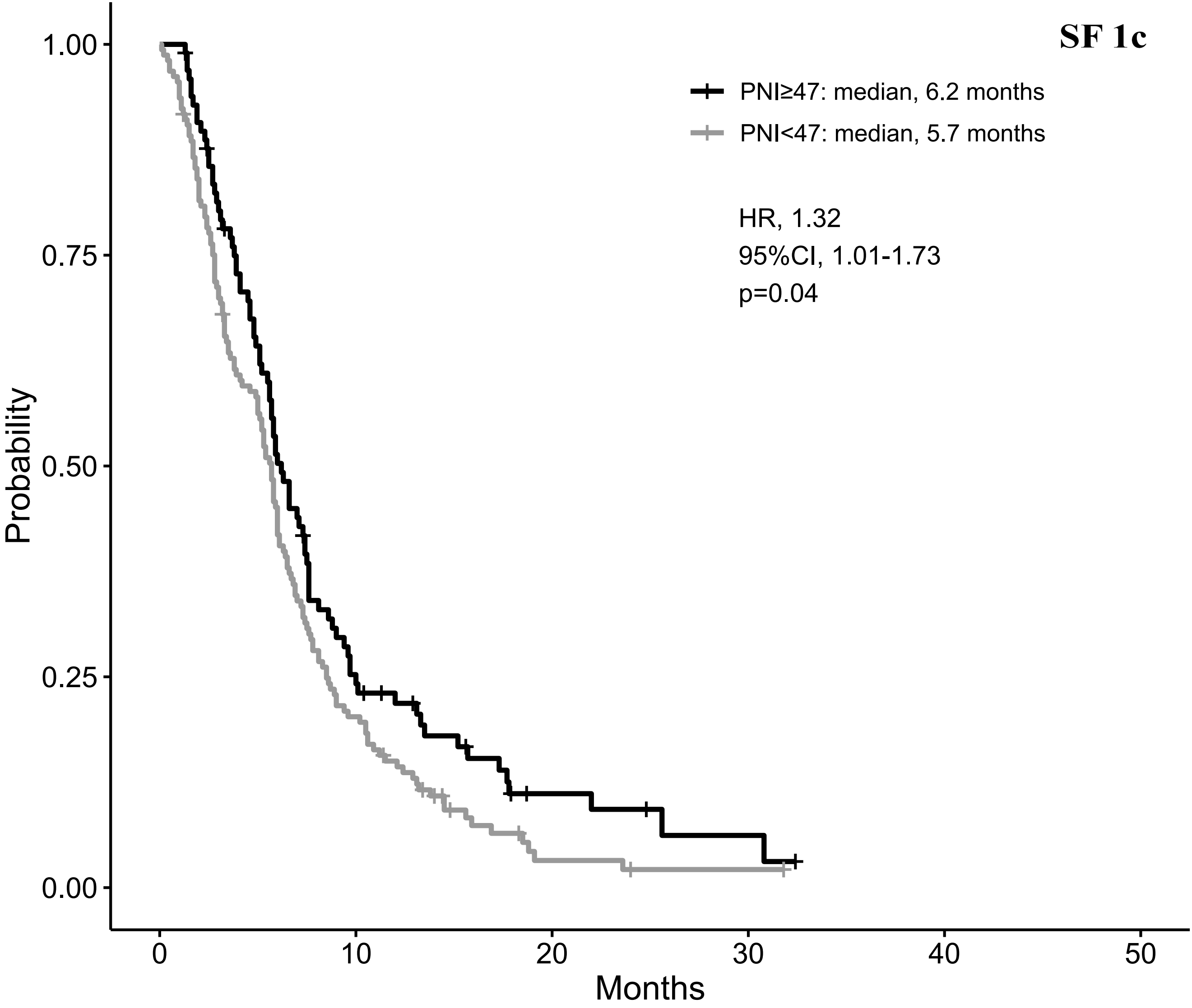


No. at risk

| | 0 | 10 | 20 | 30 | 40 | 50 |
|---|---|---|---|---|---|---|
| PNI≥47 | 98 | 23 | 6 | 2 | 0 | 0 |
| PNI<47 | 157 | 31 | 3 | 1 | 0 | 0 |

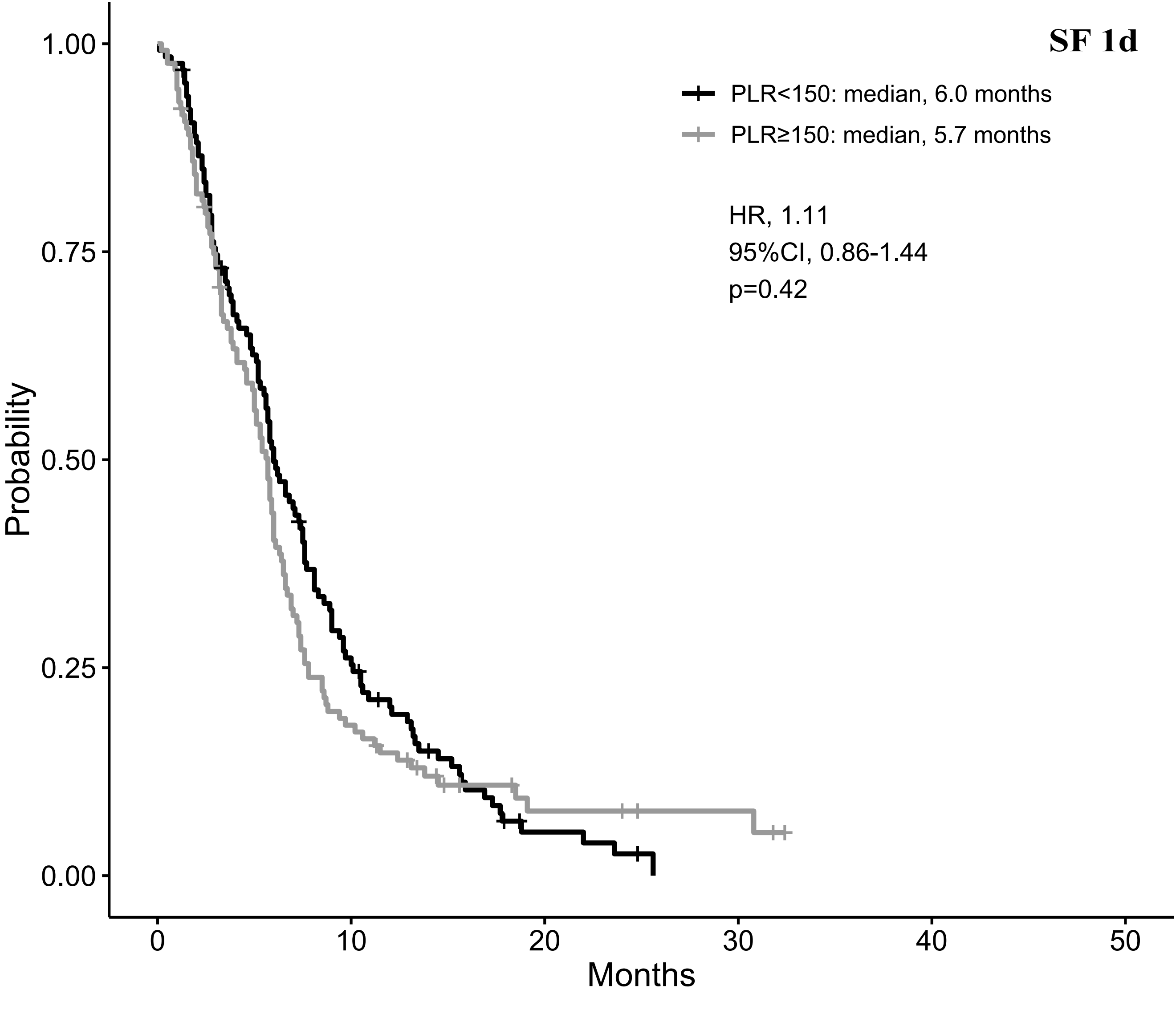


No. at risk

| | | | | | | |
|---|---|---|---|---|---|---|
| PLR<150 | 127 | 32 | 4 | 0 | 0 | 0 |
| PLR≥150 | 128 | 22 | 5 | 3 | 0 | 0 |

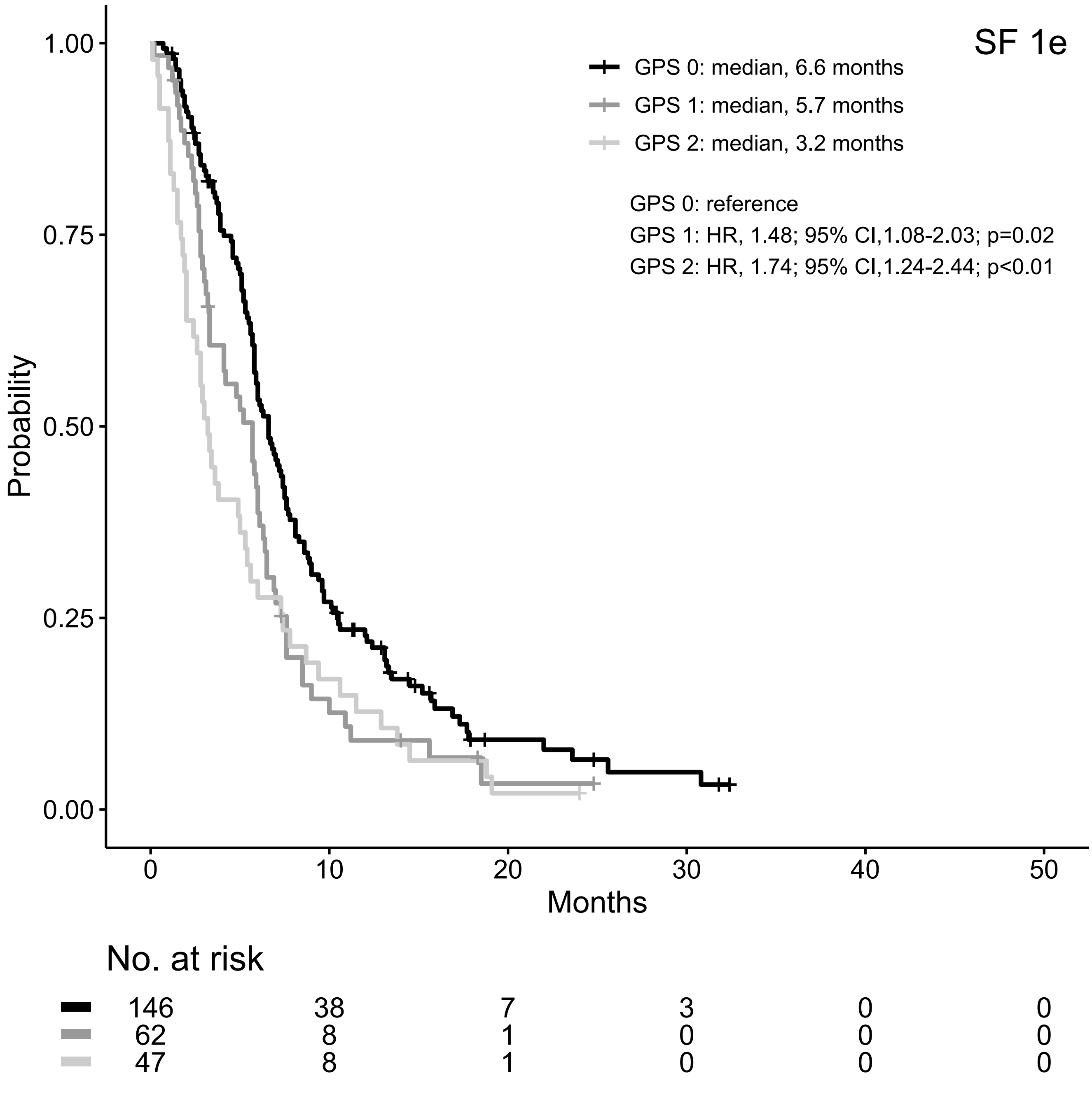
SF 1e
GPS 0: median, 6.6 months
GPS 1: median, 5.7 months
GPS 2: median, 3.2 months
GPS 0: reference
GPS 1: HR, 1.48; 95% CI,1.08-2.03; p=0.02
GPS 2: HR, 1.74; 95% CI,1.24-2.44; p<0.01
Probability
1.00
0.75
0.50
0.25
0.00
0
10
20
30
40
50
Months
No. at risk
146 38 7 3 0 0
62 8 1 0 0 0
47 8 1 0 0 0

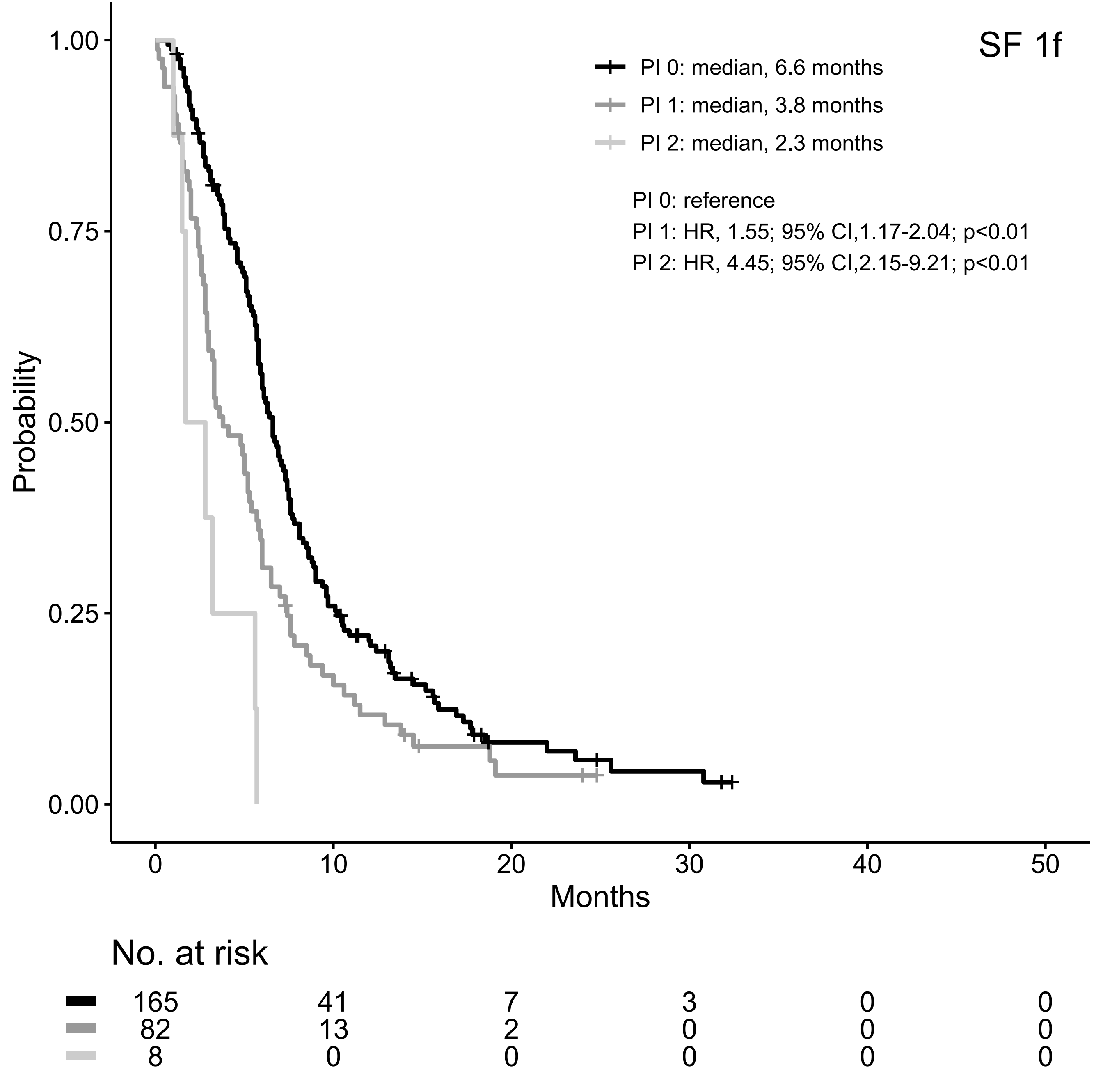

SF 1f
PI 0: median, 6.6 months
PI 1: median, 3.8 months
PI 2: median, 2.3 months
PI 0: reference
PI 1: HR, 1.55; 95% CI,1.17-2.04; p<0.01
PI 2: HR, 4.45; 95% CI,2.15-9.21; p<0.01
Probability
1.00
0.75
0.50
0.25
0.00
0
10
20
30
40
50
Months
No. at risk
165 41 7 3 0 0
82 13 2 0 0 0
8 0 0 0 0 0

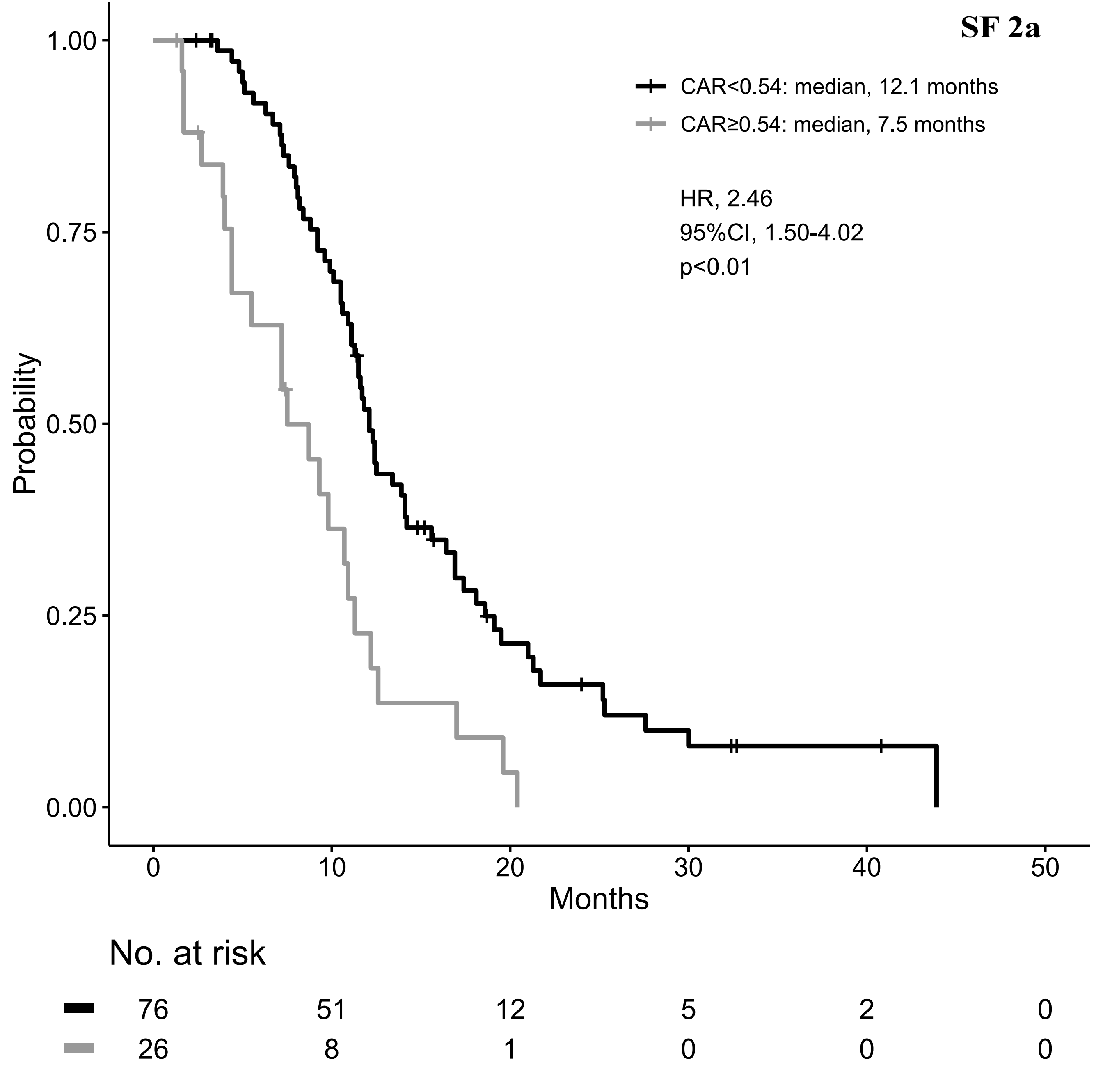
SF 2a
CAR<0.54: median, 12.1 months
CAR≥0.54: median, 7.5 months
HR, 2.46
95%CI, 1.50-4.02
p<0.01
Probability
1.00
0.75
0.50
0.25
0.00
0
10
20
30
40
50
Months
No. at risk
76 51 12 5 2 0
26 8 1 0 0 0

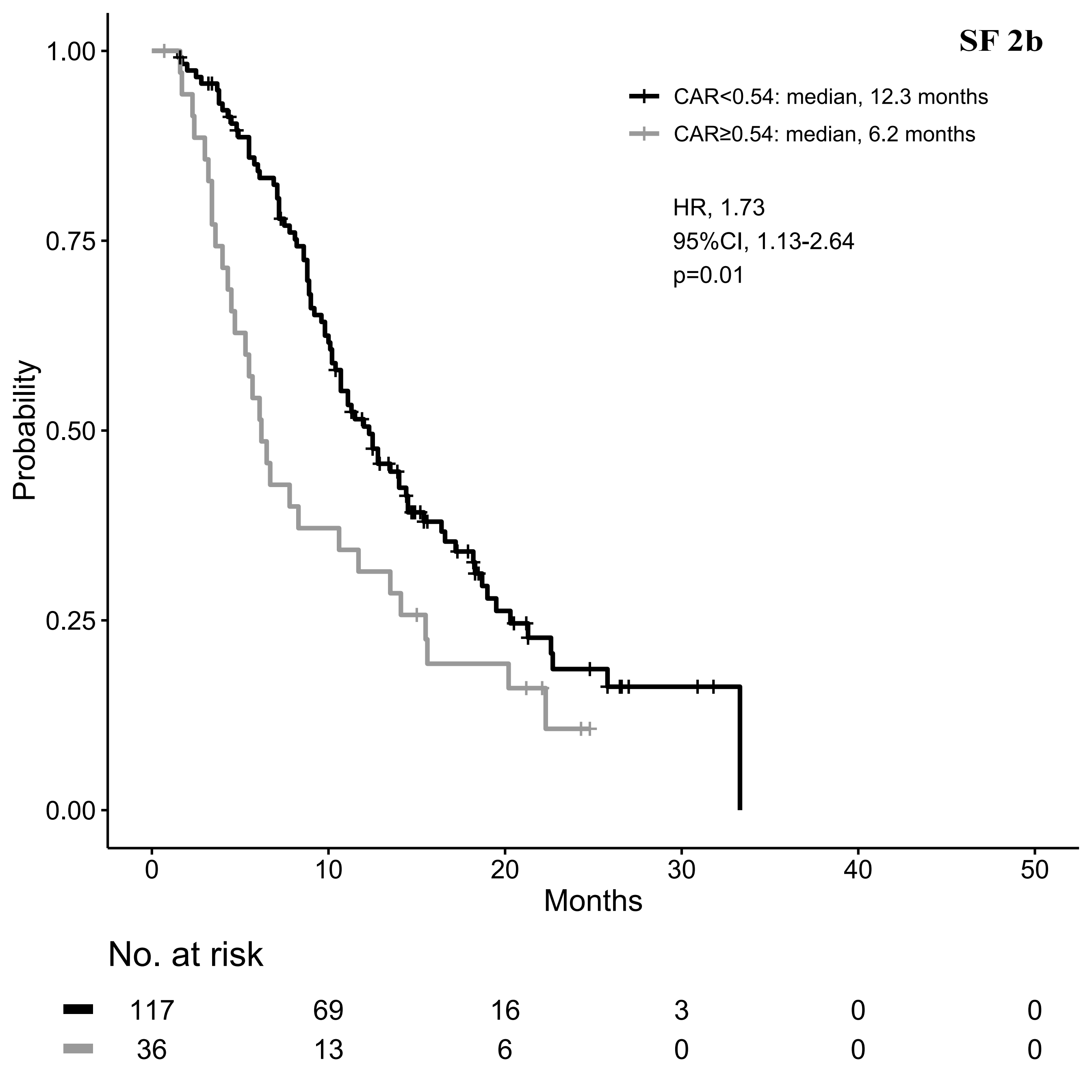
SF 2b
CAR<0.54: median, 12.3 months
CAR≥0.54: median, 6.2 months
HR, 1.73
95%CI, 1.13-2.64
p=0.01
Probability
1.00
0.75
0.50
0.25
0.00
0
10
20
30
40
50
Months
No. at risk
117 69 16 3 0 0
36 13 6 0 0 0

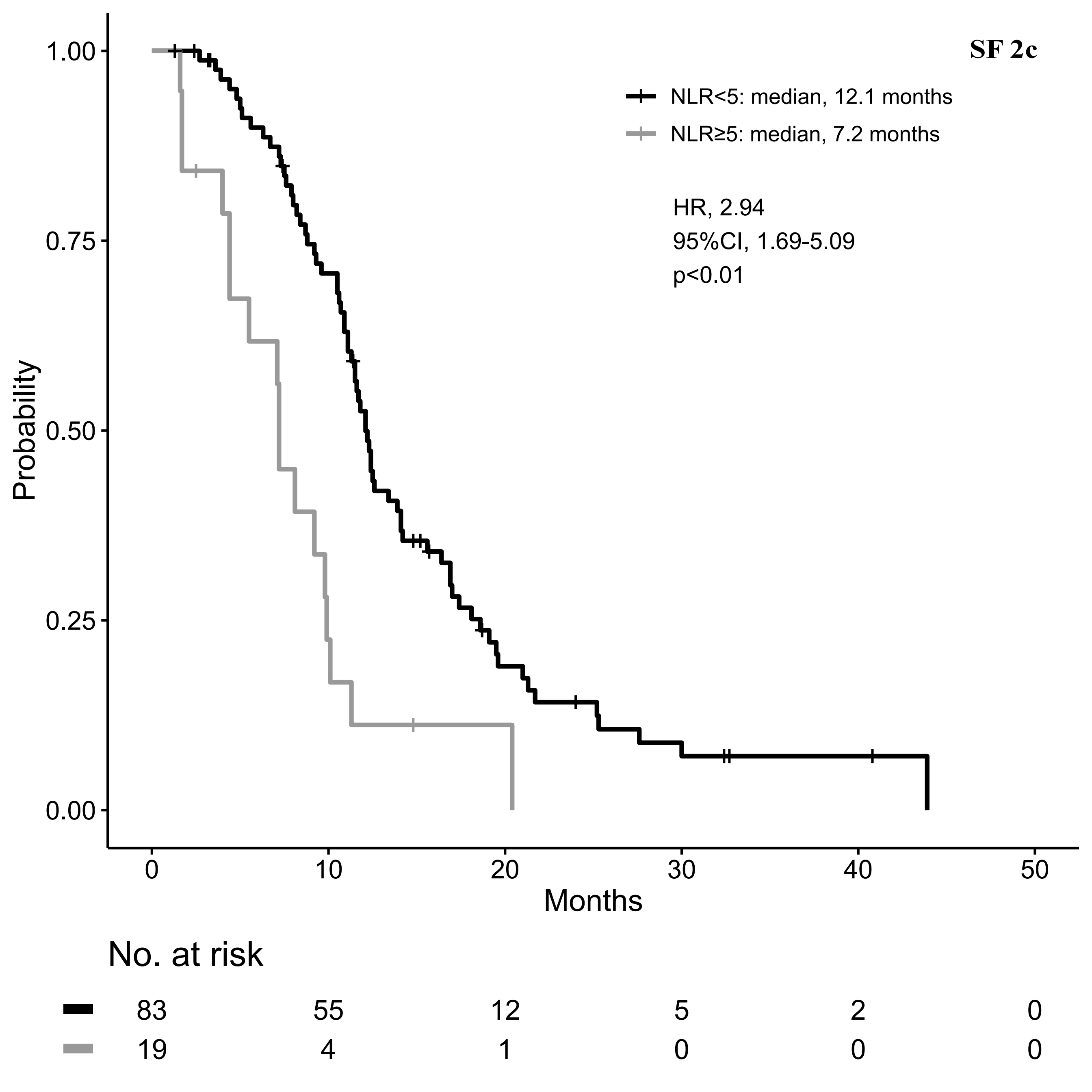
SF 2c
NLR<5: median, 12.1 months
NLR≥5: median, 7.2 months
HR, 2.94
95%CI, 1.69-5.09
p<0.01
Probability
1.00
0.75
0.50
0.25
0.00
0
10
20
30
40
50
Months
No. at risk
83 55 12 5 2 0
19 4 1 0 0 0

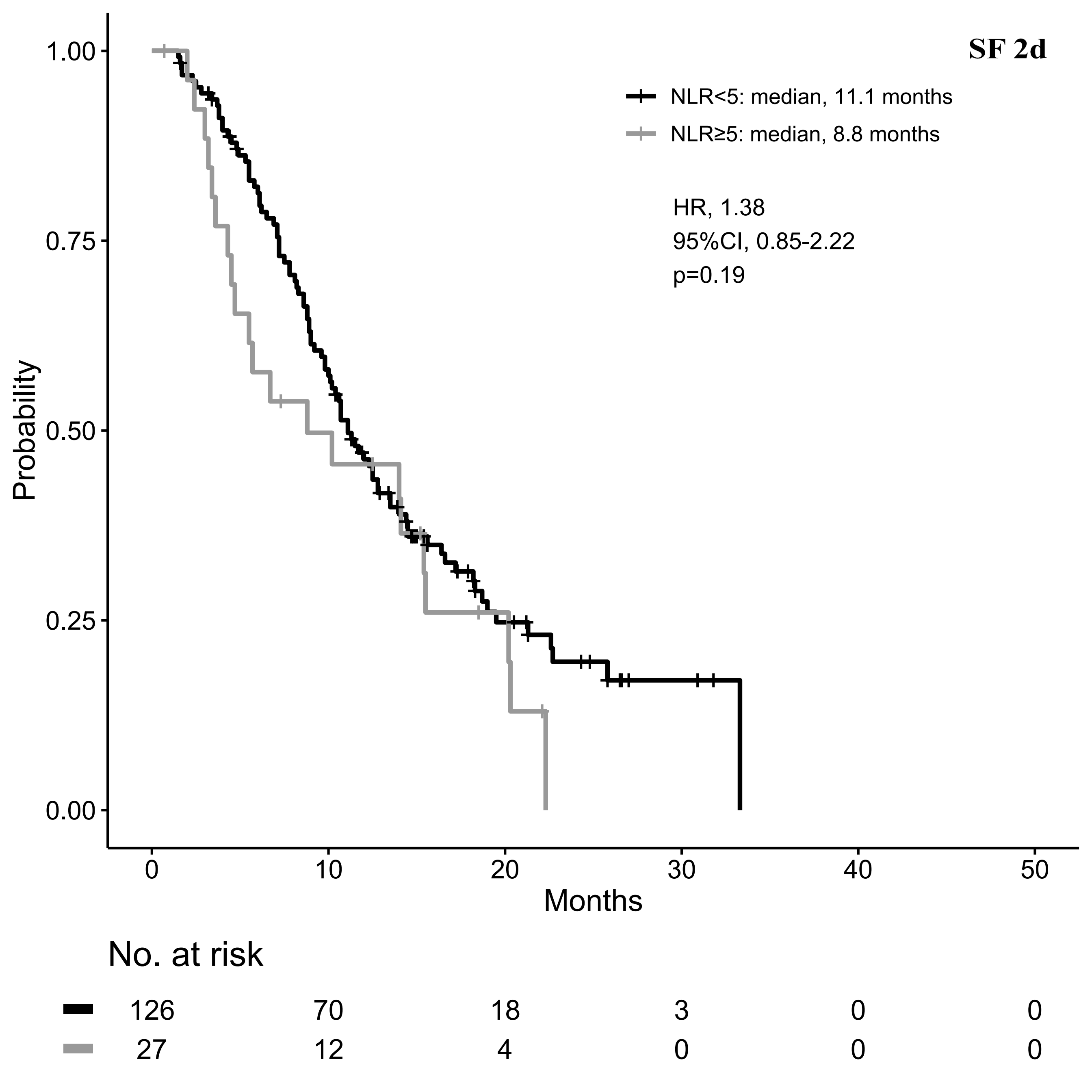
SF 2d
NLR<5: median, 11.1 months
NLR≥5: median, 8.8 months
HR, 1.38
95%CI, 0.85-2.22
p=0.19
Probability
1.00
0.75
0.50
0.25
0.00
0
10
20
30
40
50
Months
No. at risk
126
70
18
3
0
0
27
12
4
0
0
0

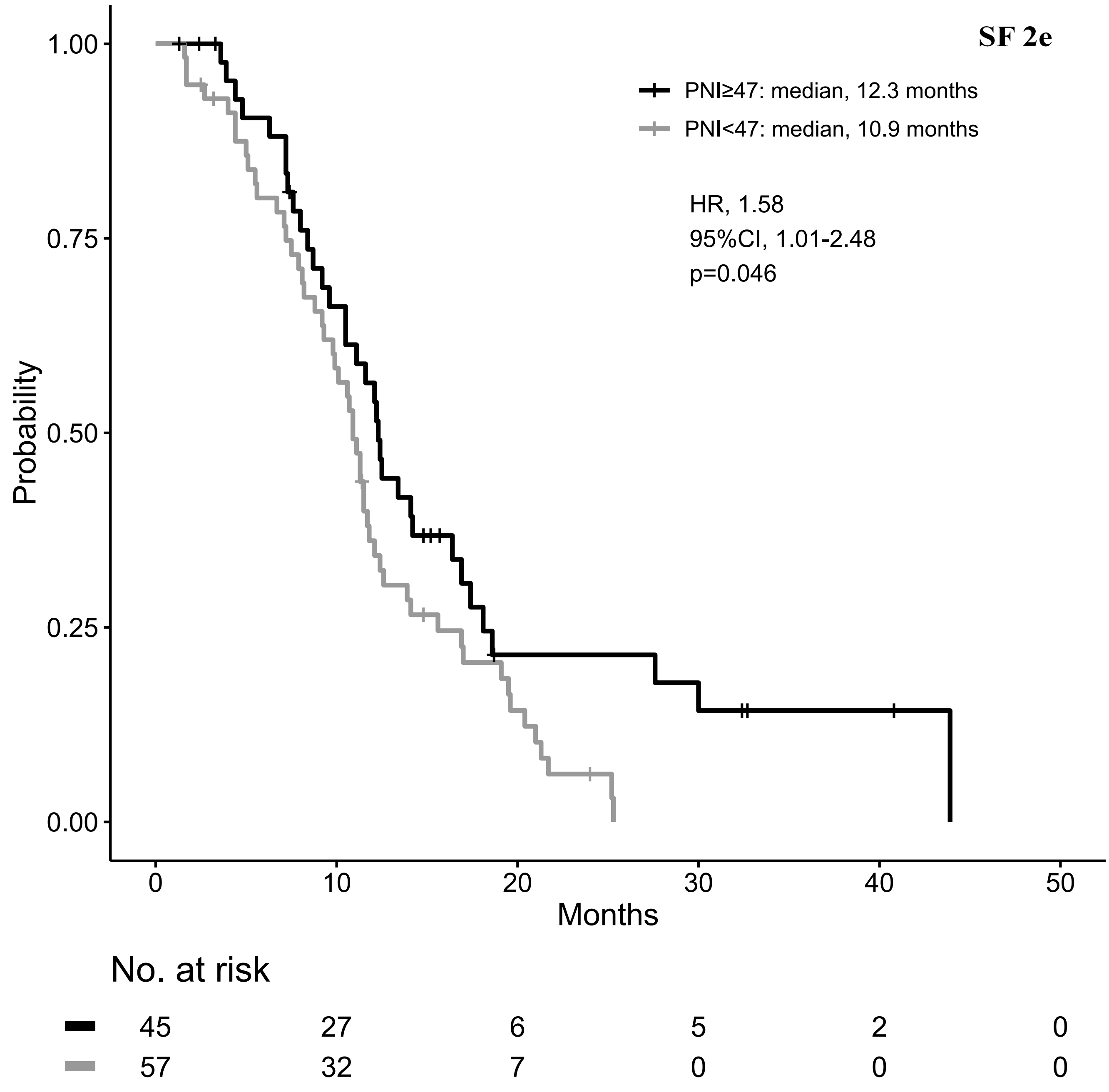

SF 2e
PNI≥47: median, 12.3 months
PNI<47: median, 10.9 months
HR, 1.58
95%CI, 1.01-2.48
p=0.046
Probability
1.00
0.75
0.50
0.25
0.00
0
10
20
30
40
50
Months
No. at risk
45
27
6
5
2
0
57
32
7
0
0
0

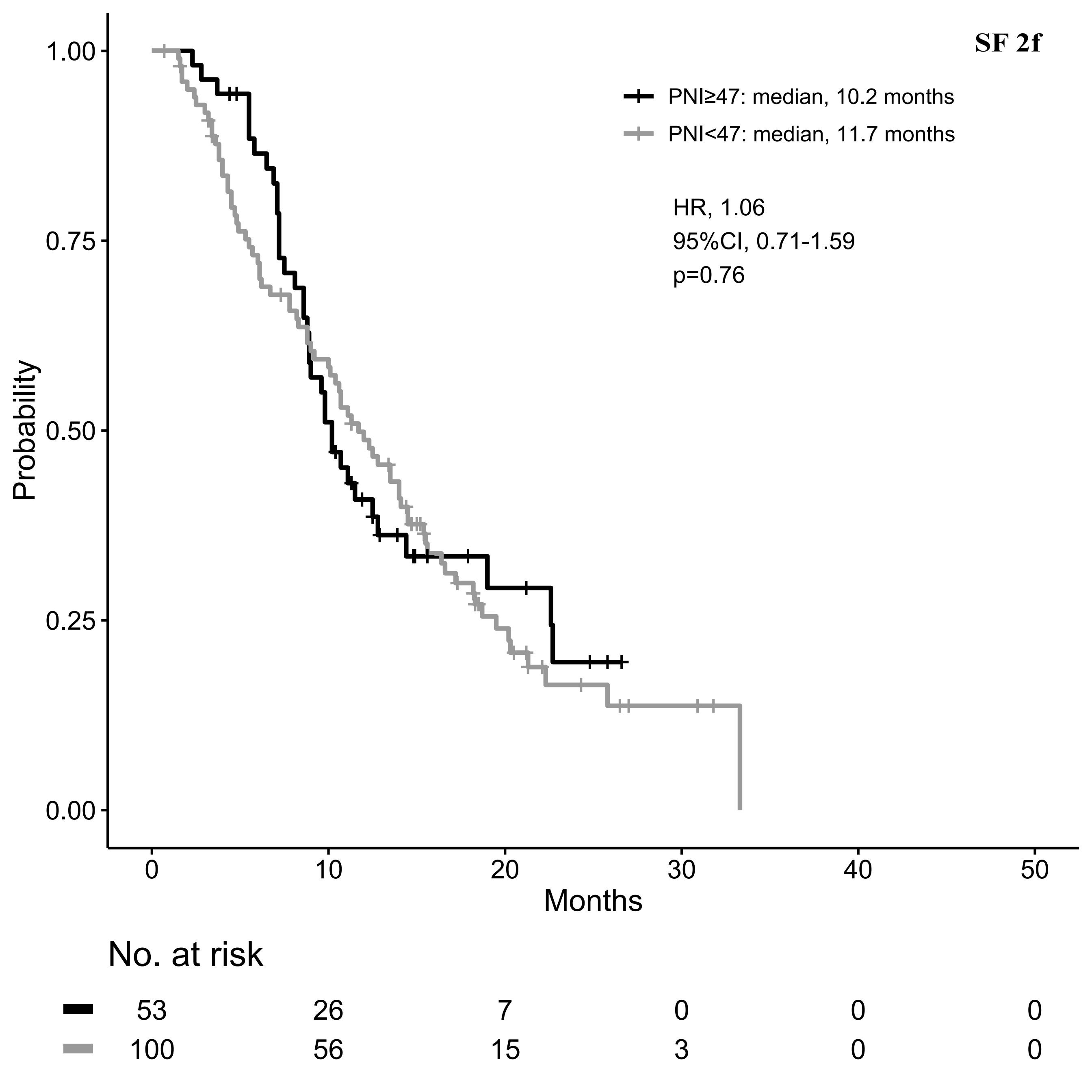

SF 2f
PNI≥47: median, 10.2 months
PNI<47: median, 11.7 months
HR, 1.06
95%CI, 0.71-1.59
p=0.76
Probability
1.00
0.75
0.50
0.25
0.00
0
10
20
30
40
50
Months
No. at risk
53 26 7 0 0 0
100 56 15 3 0 0

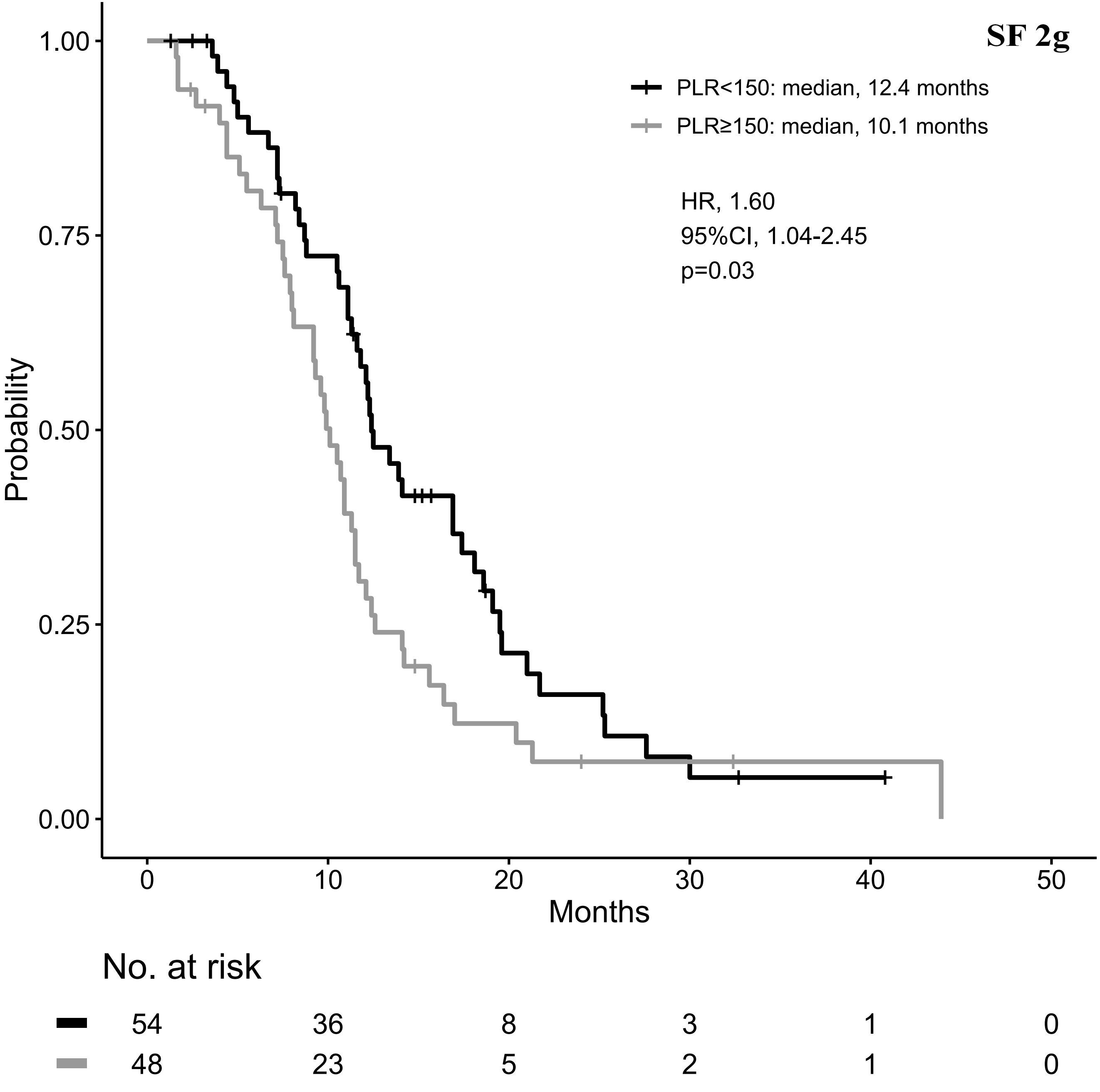
SF 2g
PLR<150: median, 12.4 months
PLR≥150: median, 10.1 months
HR, 1.60
95%CI, 1.04-2.45
p=0.03
Probability
1.00
0.75
0.50
0.25
0.00
0
10
20
30
40
50
Months
No. at risk
54 36 8 3 1 0
48 23 5 2 1 0

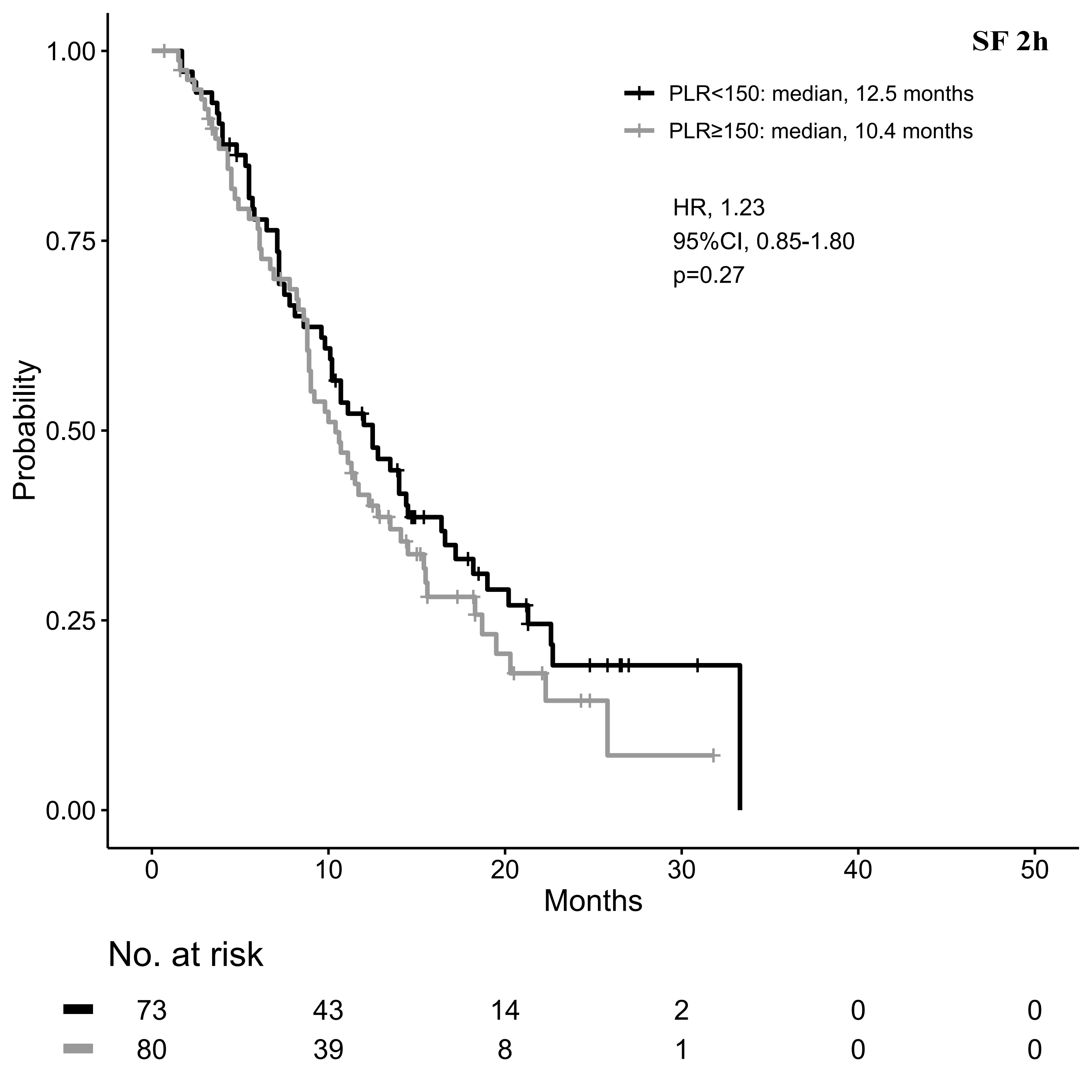

SF 2h
PLR<150: median, 12.5 months
PLR≥150: median, 10.4 months
HR, 1.23
95%CI, 0.85-1.80
p=0.27
Probability
1.00
0.75
0.50
0.25
0.00
0
10
20
30
40
50
Months
No. at risk
73 43 14 2 0 0
80 39 8 1 0 0

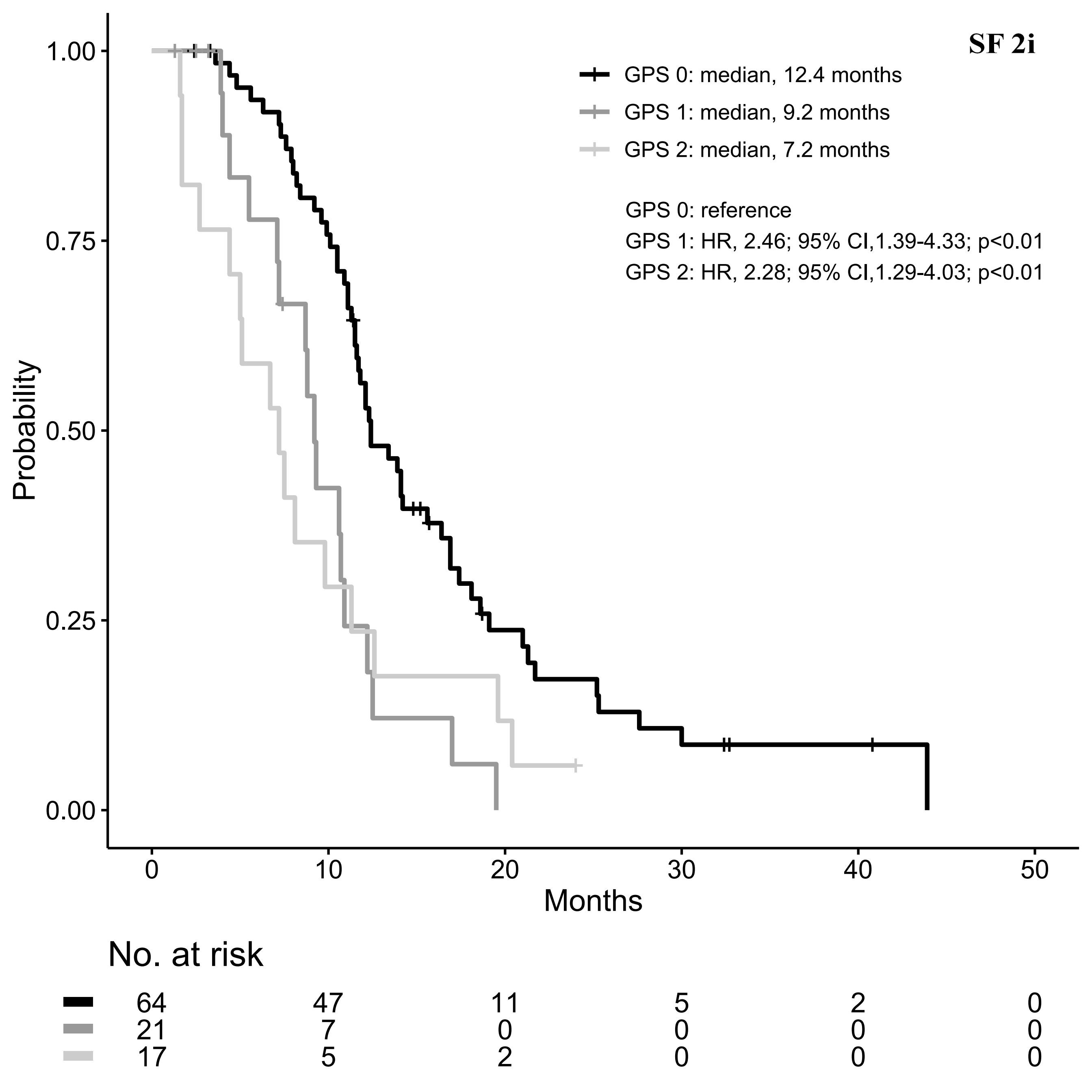
SF 2i
GPS 0: median, 12.4 months
GPS 1: median, 9.2 months
GPS 2: median, 7.2 months
GPS 0: reference
GPS 1: HR, 2.46; 95% CI,1.39-4.33; p<0.01
GPS 2: HR, 2.28; 95% CI,1.29-4.03; p<0.01
Probability
1.00
0.75
0.50
0.25
0.00
0
10
20
30
40
50
Months
No. at risk
64 47 11 5 2 0
21 7 0 0 0 0
17 5 2 0 0 0

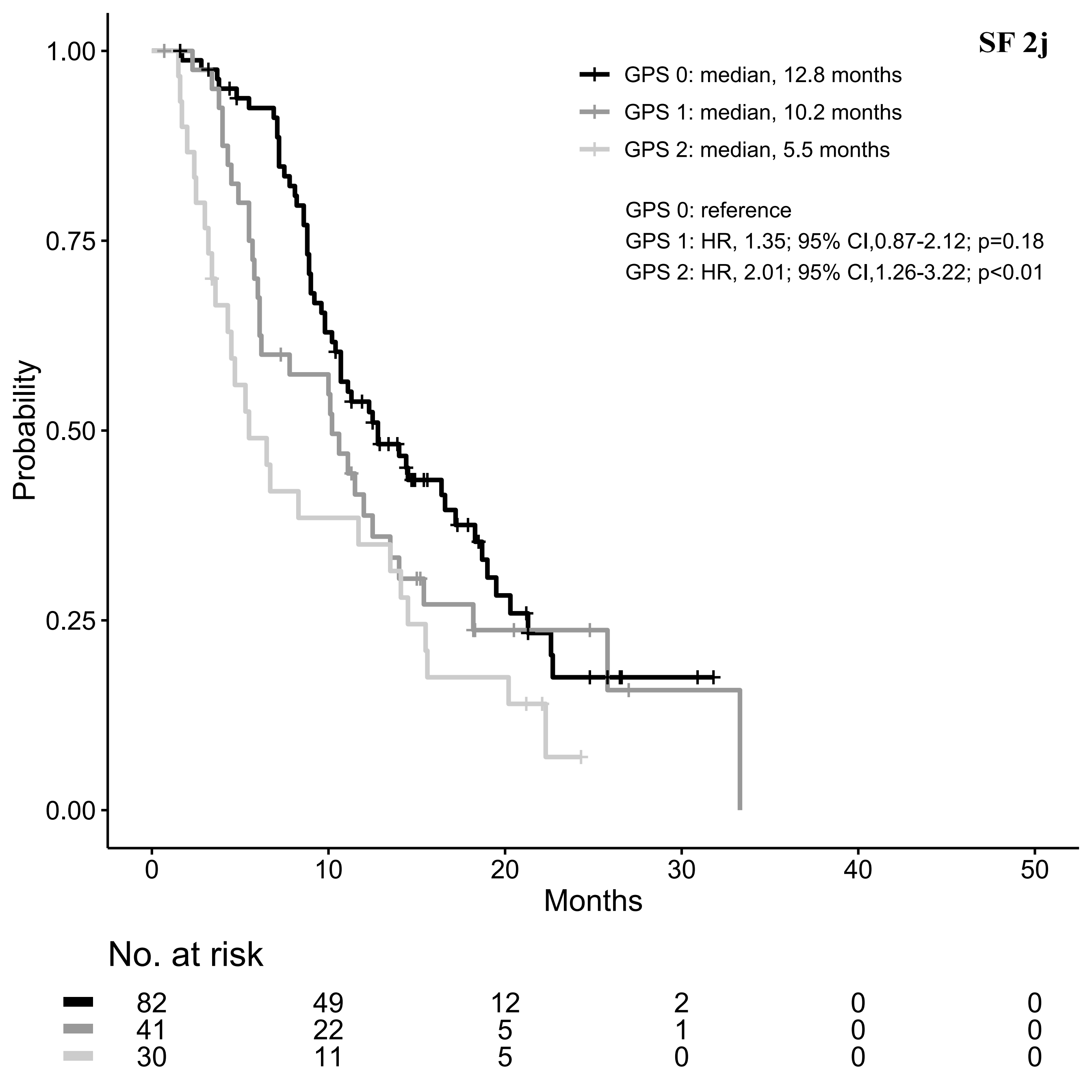
SF 2j
GPS 0: median, 12.8 months
GPS 1: median, 10.2 months
GPS 2: median, 5.5 months
GPS 0: reference
GPS 1: HR, 1.35; 95% CI,0.87-2.12; p=0.18
GPS 2: HR, 2.01; 95% CI,1.26-3.22; p<0.01
Probability
1.00
0.75
0.50
0.25
0.00
0
10
20
30
40
50
Months
No. at risk
82 49 12 2 0 0
41 22 5 1 0 0
30 11 5 0 0 0

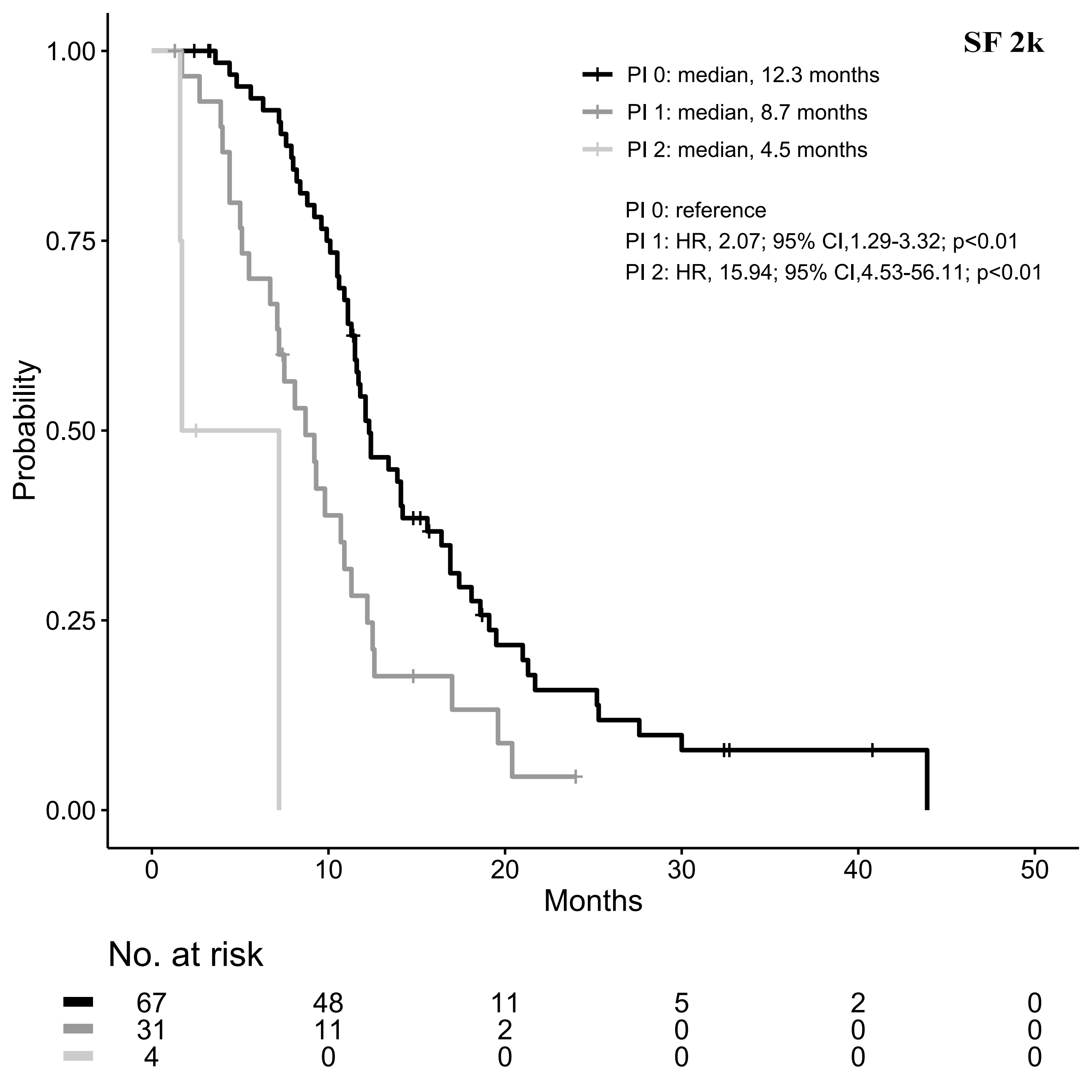

SF 2k
PI 0: median, 12.3 months
PI 1: median, 8.7 months
PI 2: median, 4.5 months
PI 0: reference
PI 1: HR, 2.07; 95% CI,1.29-3.32; p<0.01
PI 2: HR, 15.94; 95% CI,4.53-56.11; p<0.01
Probability
1.00
0.75
0.50
0.25
0.00
0
10
20
30
40
50
Months
No. at risk
67 48 11 5 2 0
31 11 2 0 0 0
4 0 0 0 0 0

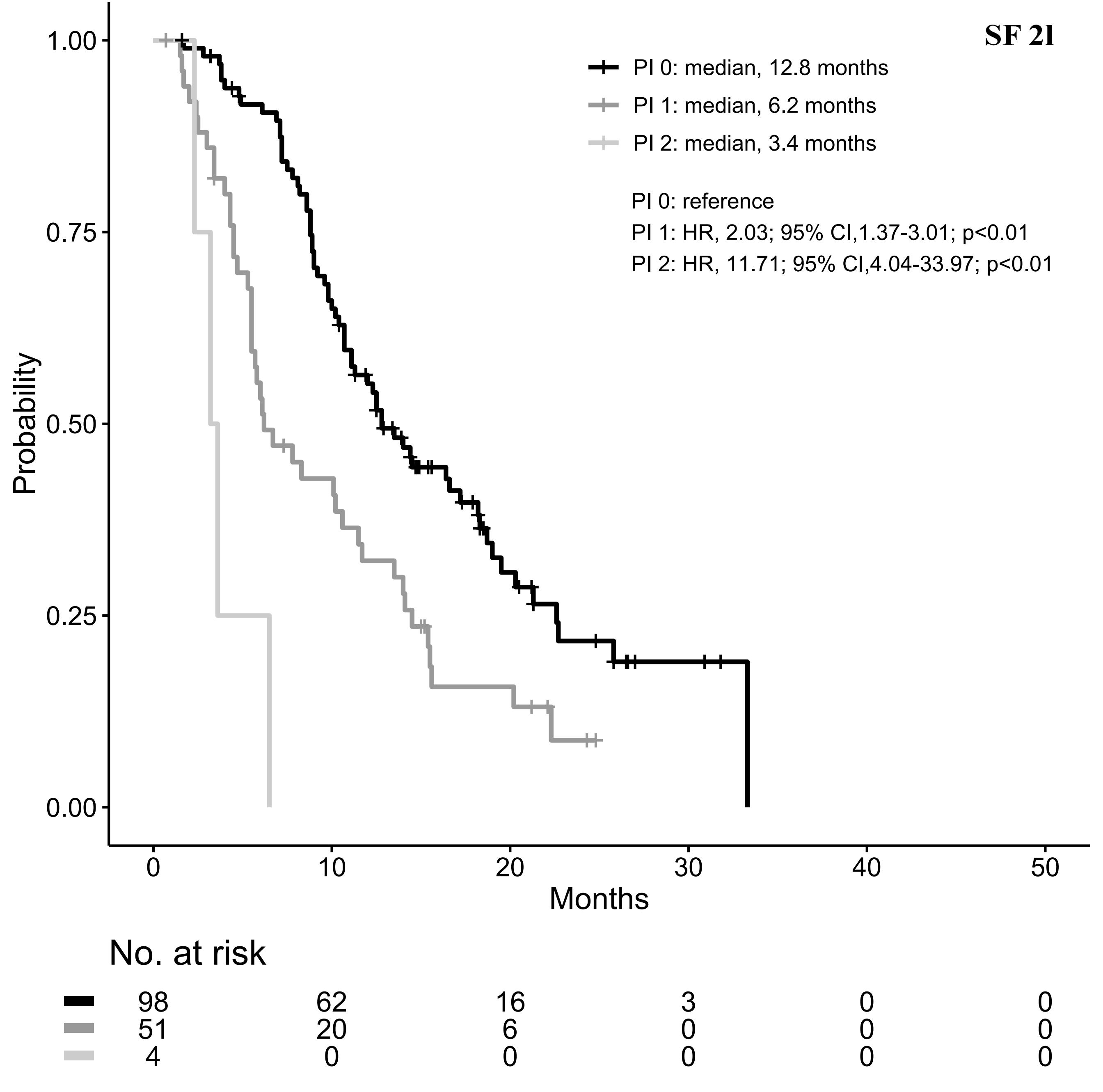
SF 2l
PI 0: median, 12.8 months
PI 1: median, 6.2 months
PI 2: median, 3.4 months
PI 0: reference
PI 1: HR, 2.03; 95% CI,1.37-3.01; p<0.01
PI 2: HR, 11.71; 95% CI,4.04-33.97; p<0.01
Probability
1.00
0.75
0.50
0.25
0.00
0
10
20
30
40
50
Months
No. at risk
98 62 16 3 0 0
51 20 6 0 0 0
4 0 0 0 0 0

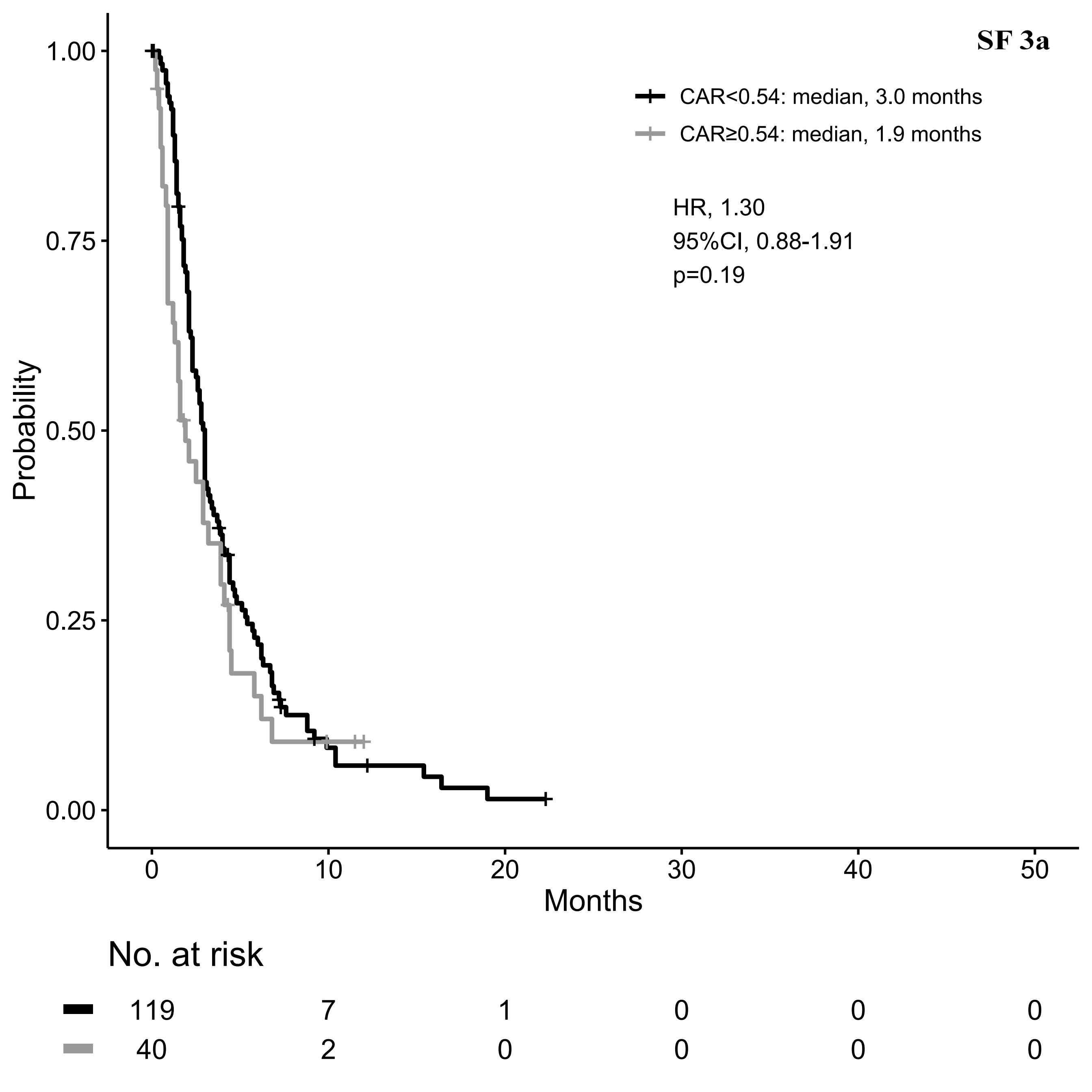
SF 3a
CAR<0.54: median, 3.0 months
CAR≥0.54: median, 1.9 months
HR, 1.30
95%CI, 0.88-1.91
p=0.19
Probability
1.00
0.75
0.50
0.25
0.00
0
10
20
30
40
50
Months
No. at risk
119
7
1
0
0
0
40
2
0
0
0
0

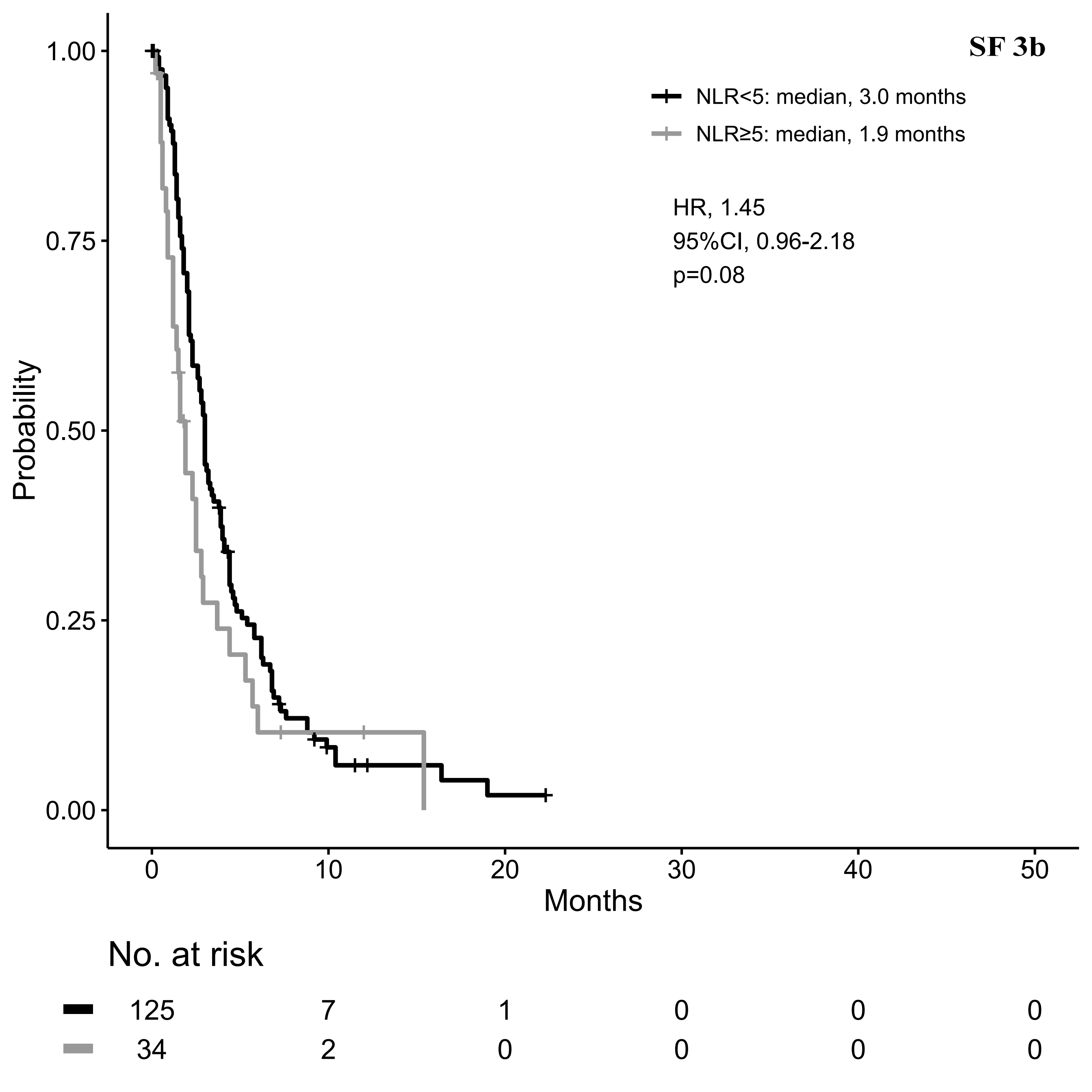

SF 3b
NLR<5: median, 3.0 months
NLR≥5: median, 1.9 months
HR, 1.45
95%CI, 0.96-2.18
p=0.08
Probability
1.00
0.75
0.50
0.25
0.00
0
10
20
30
40
50
Months
No. at risk
125 7 1 0 0 0
34 2 0 0 0 0

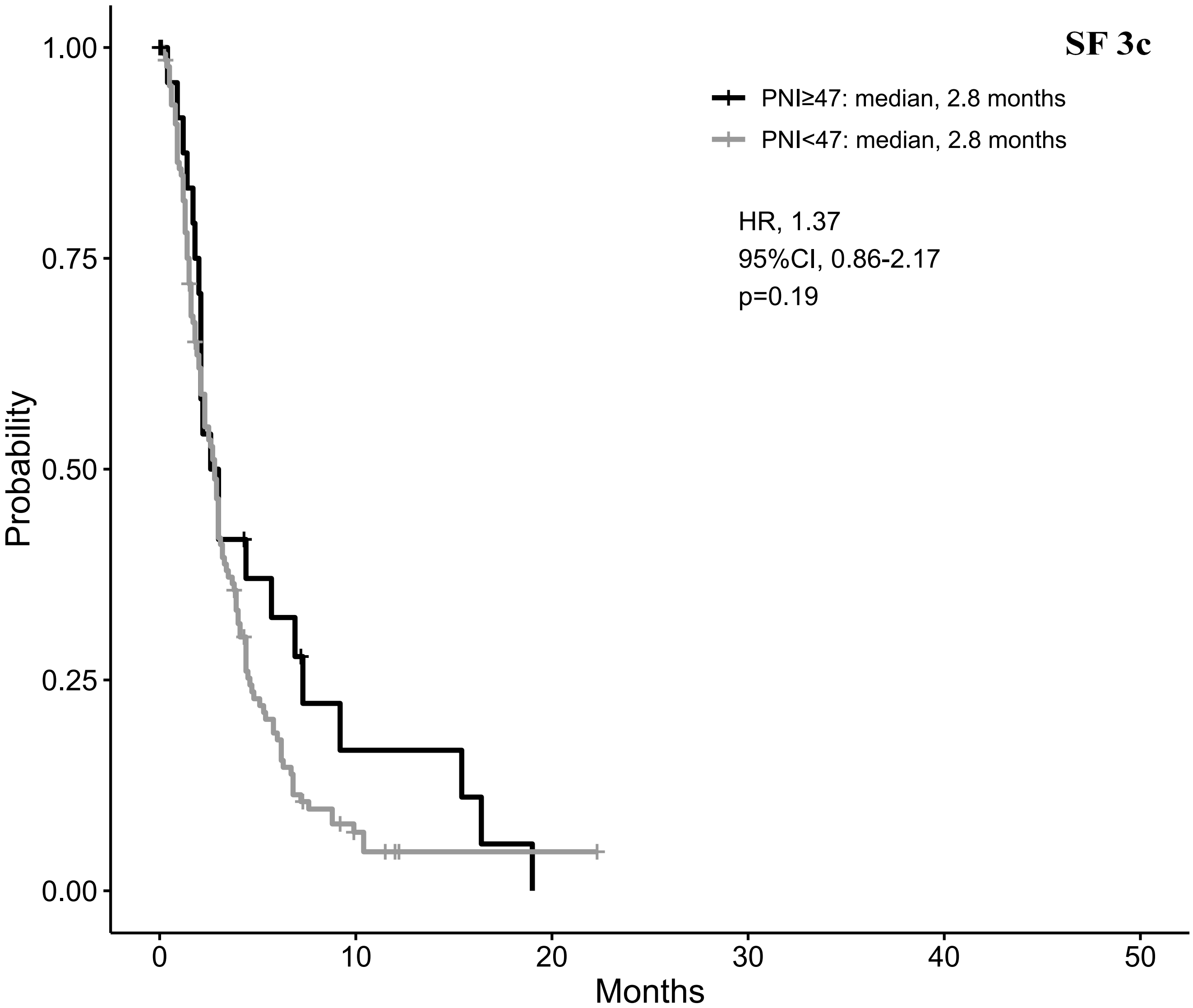


| No. at risk | | | | | | |
|---|---|---|---|---|---|---|
| PNI≥47 | 26 | 3 | 0 | 0 | 0 | 0 |
| PNI<47 | 133 | 6 | 1 | 0 | 0 | 0 |

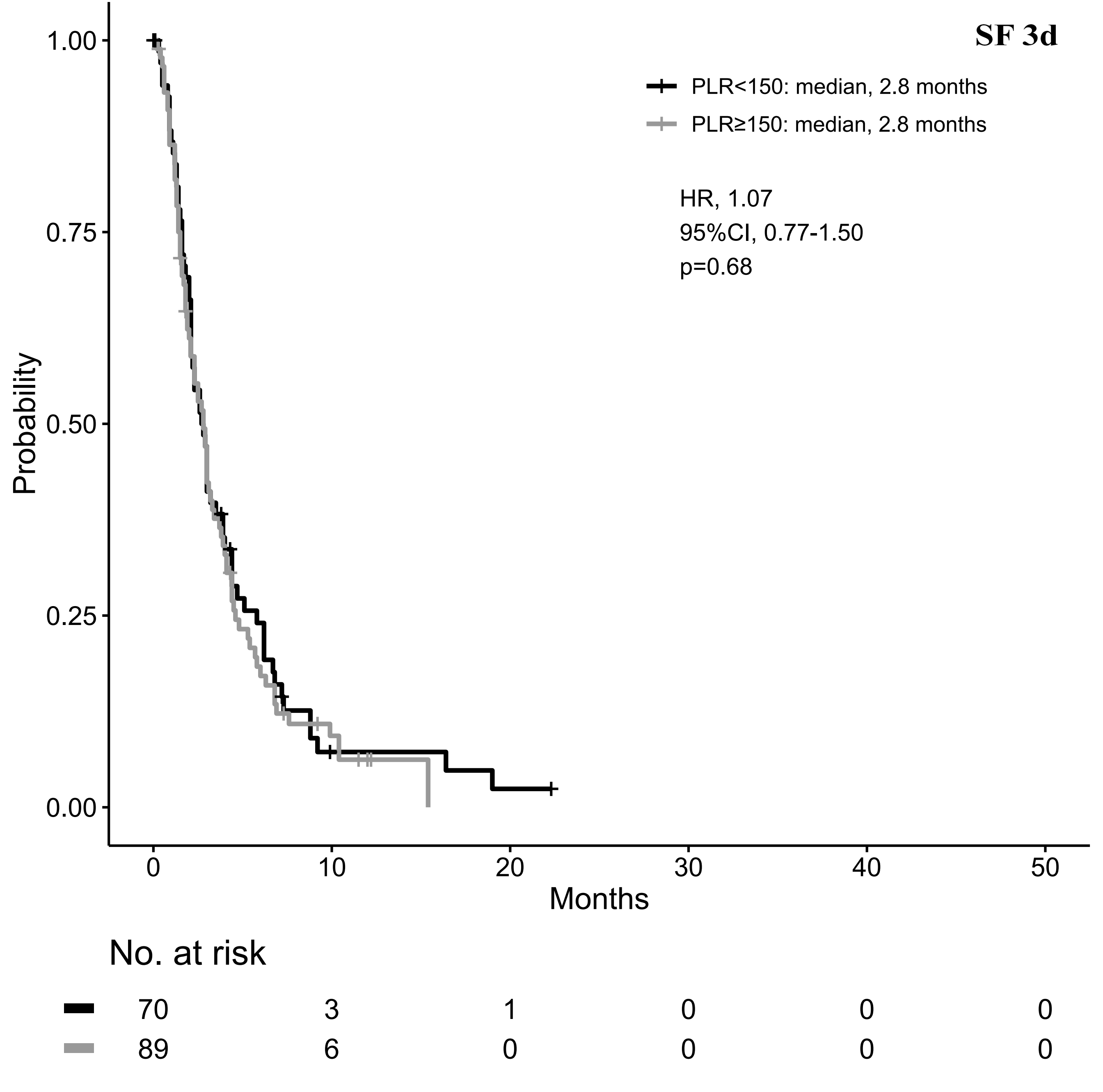

SF 3d
PLR<150: median, 2.8 months
PLR≥150: median, 2.8 months
HR, 1.07
95%CI, 0.77-1.50
p=0.68
Probability
1.00
0.75
0.50
0.25
0.00
0
10
20
30
40
50
Months
No. at risk
70 3 1 0 0 0
89 6 0 0 0 0

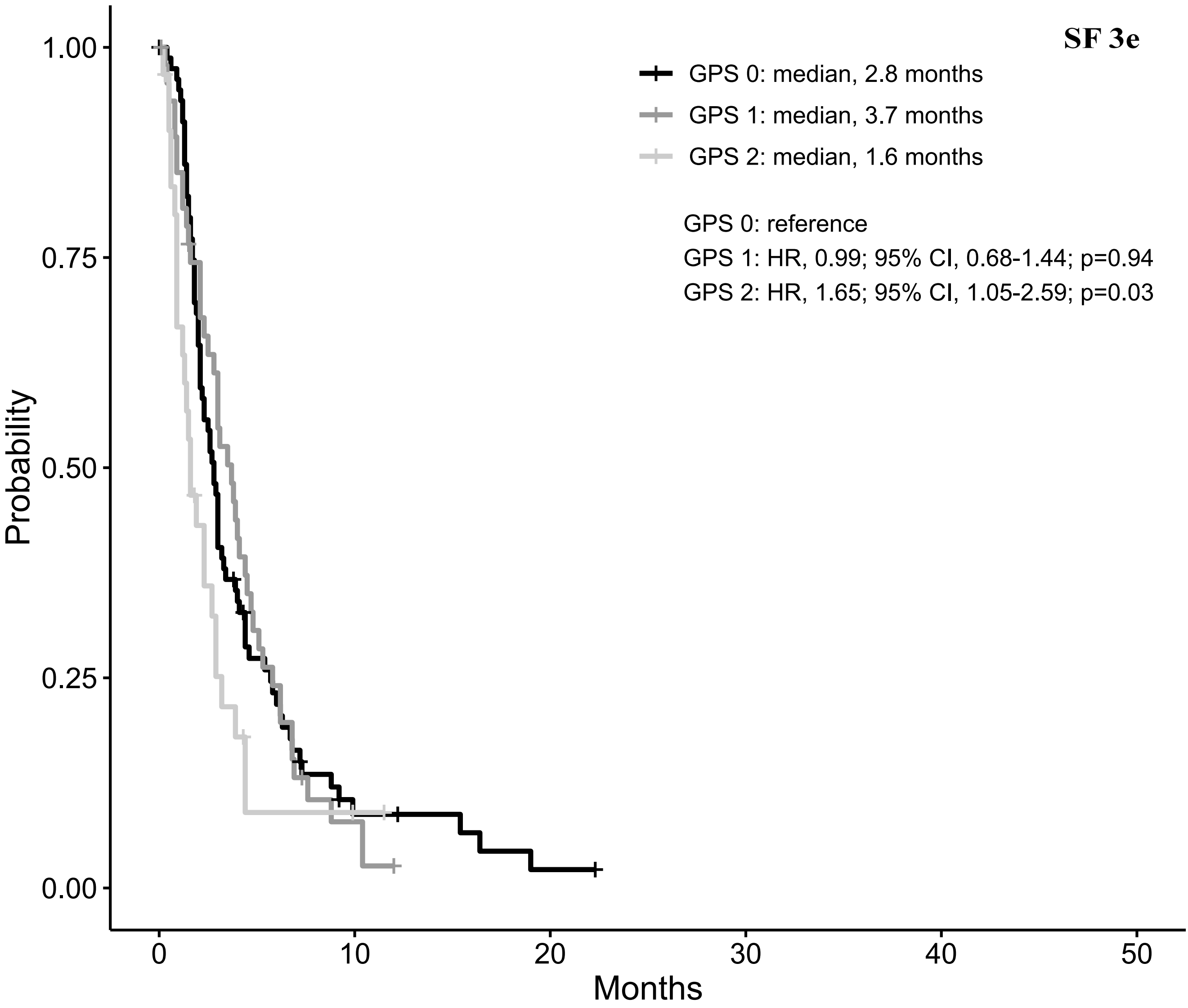


No. at risk

| | 0 | 10 | 20 | 30 | 40 | 50 |
|---|---|---|---|---|---|---|
| GPS 0 | 80 | 5 | 1 | 0 | 0 | 0 |
| GPS 1 | 48 | 3 | 0 | 0 | 0 | 0 |
| GPS 2 | 31 | 1 | 0 | 0 | 0 | 0 |

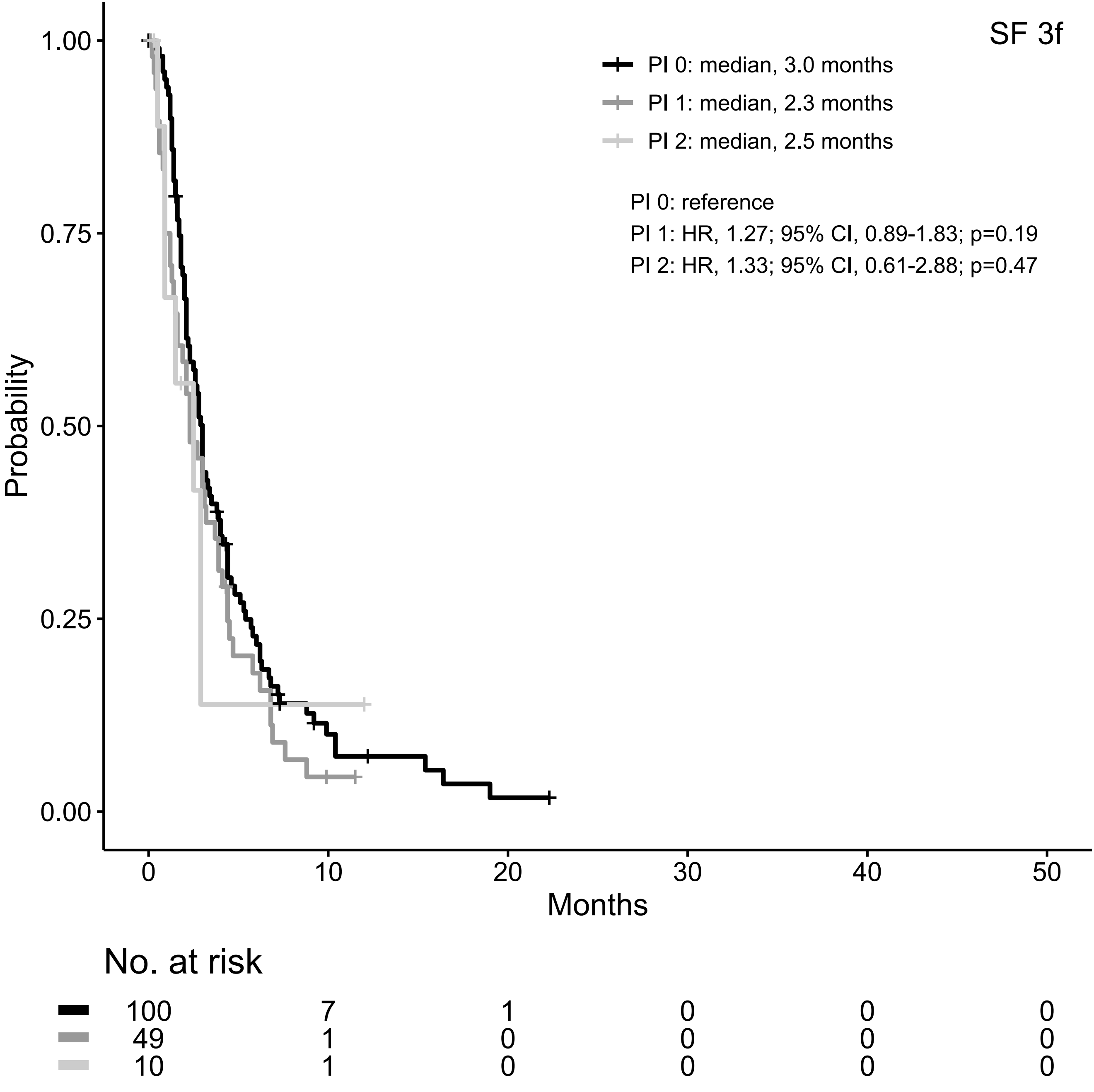
SF 3f
PI 0: median, 3.0 months
PI 1: median, 2.3 months
PI 2: median, 2.5 months
PI 0: reference
PI 1: HR, 1.27; 95% CI, 0.89-1.83; p=0.19
PI 2: HR, 1.33; 95% CI, 0.61-2.88; p=0.47
Probability
1.00
0.75
0.50
0.25
0.00
0
10
20
30
40
50
Months
No. at risk
100 7 1 0 0 0
49 1 0 0 0 0
10 1 0 0 0 0

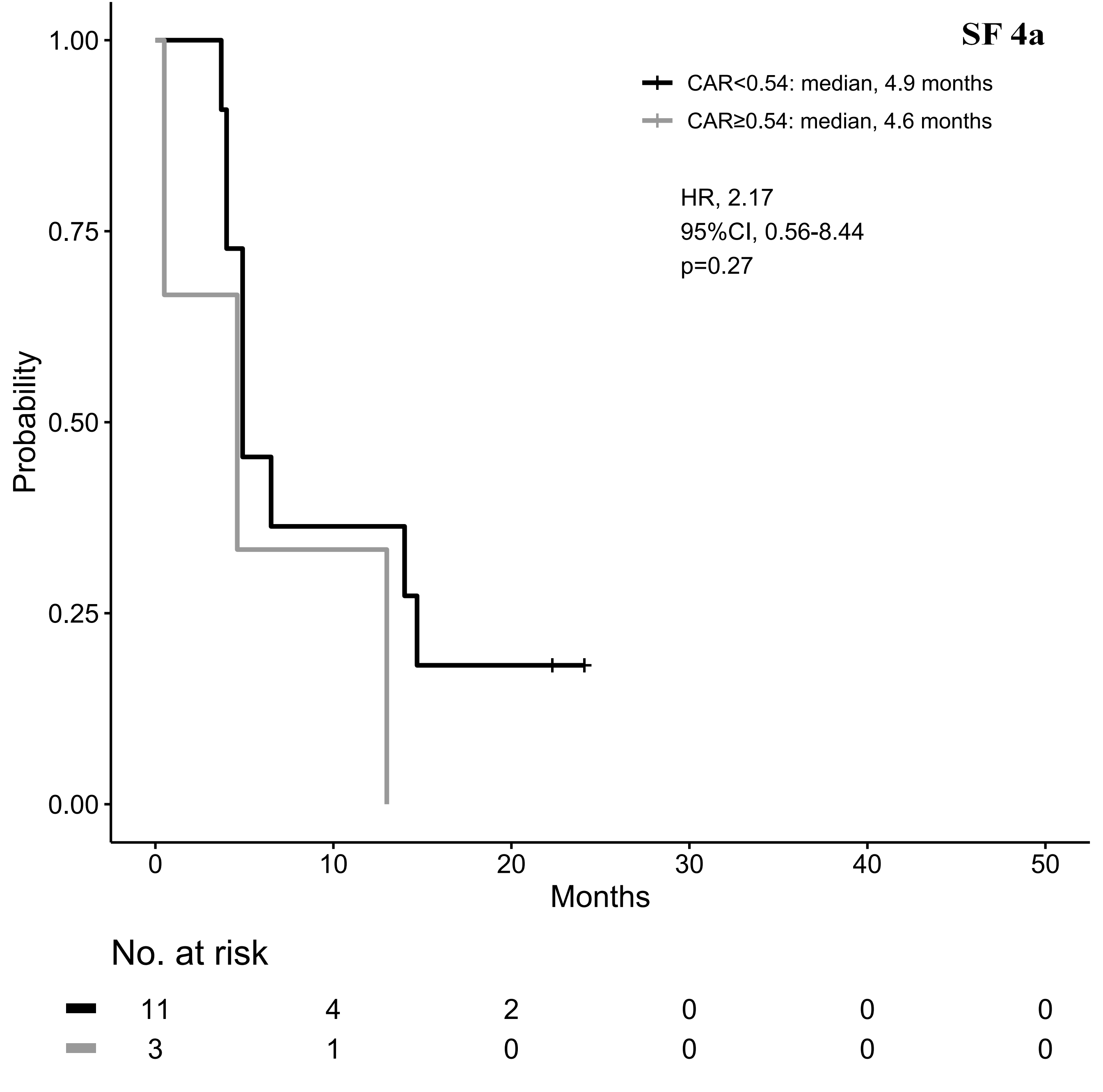

SF 4a
CAR<0.54: median, 4.9 months
CAR≥0.54: median, 4.6 months
HR, 2.17
95%CI, 0.56-8.44
p=0.27
Probability
1.00
0.75
0.50
0.25
0.00
0
10
20
30
40
50
Months
No. at risk
11
4
2
0
0
0
3
1
0
0
0
0

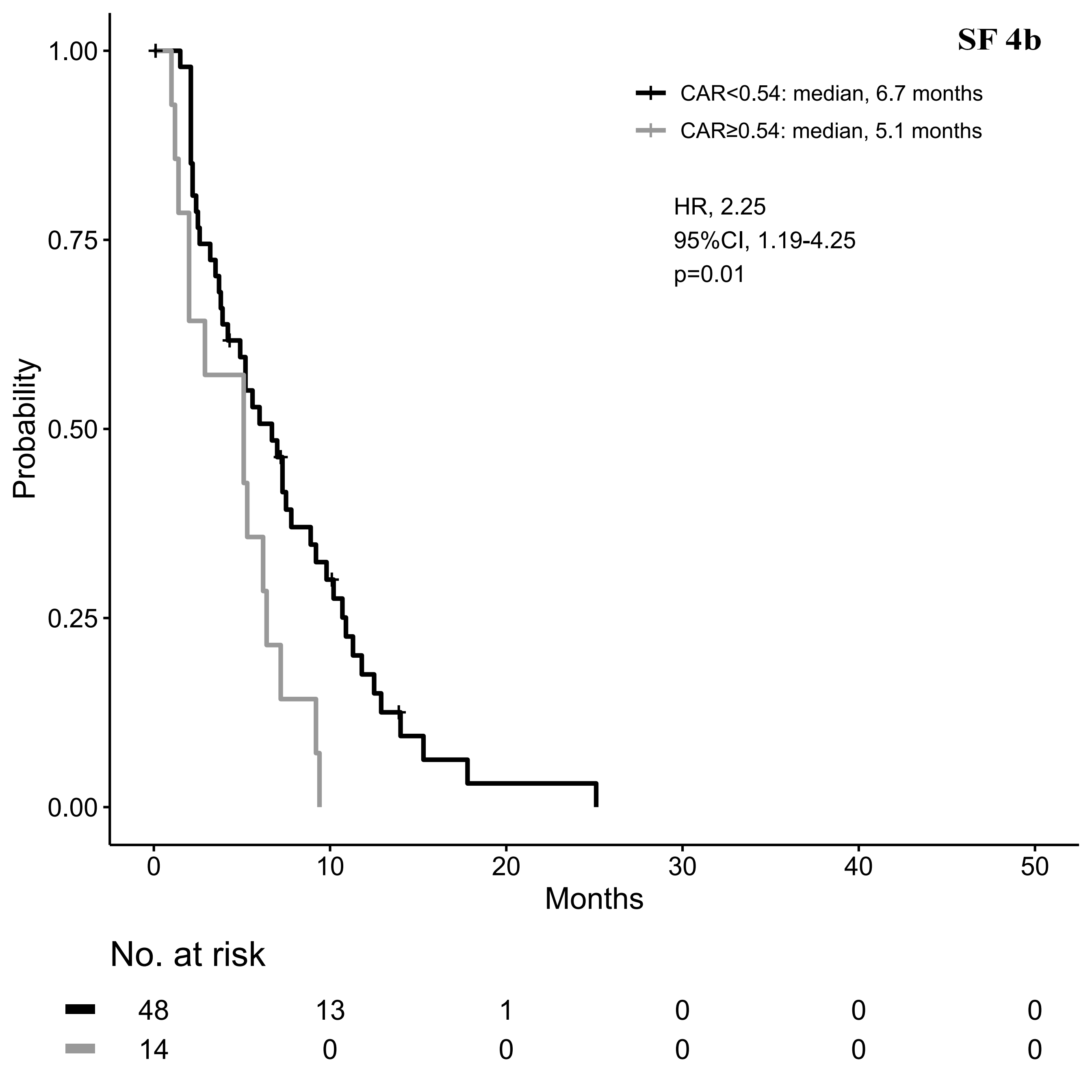
SF 4b
CAR<0.54: median, 6.7 months
CAR≥0.54: median, 5.1 months
HR, 2.25
95%CI, 1.19-4.25
p=0.01
Probability
1.00
0.75
0.50
0.25
0.00
0
10
20
30
40
50
Months
No. at risk
48
13
1
0
0
0
14
0
0
0
0
0

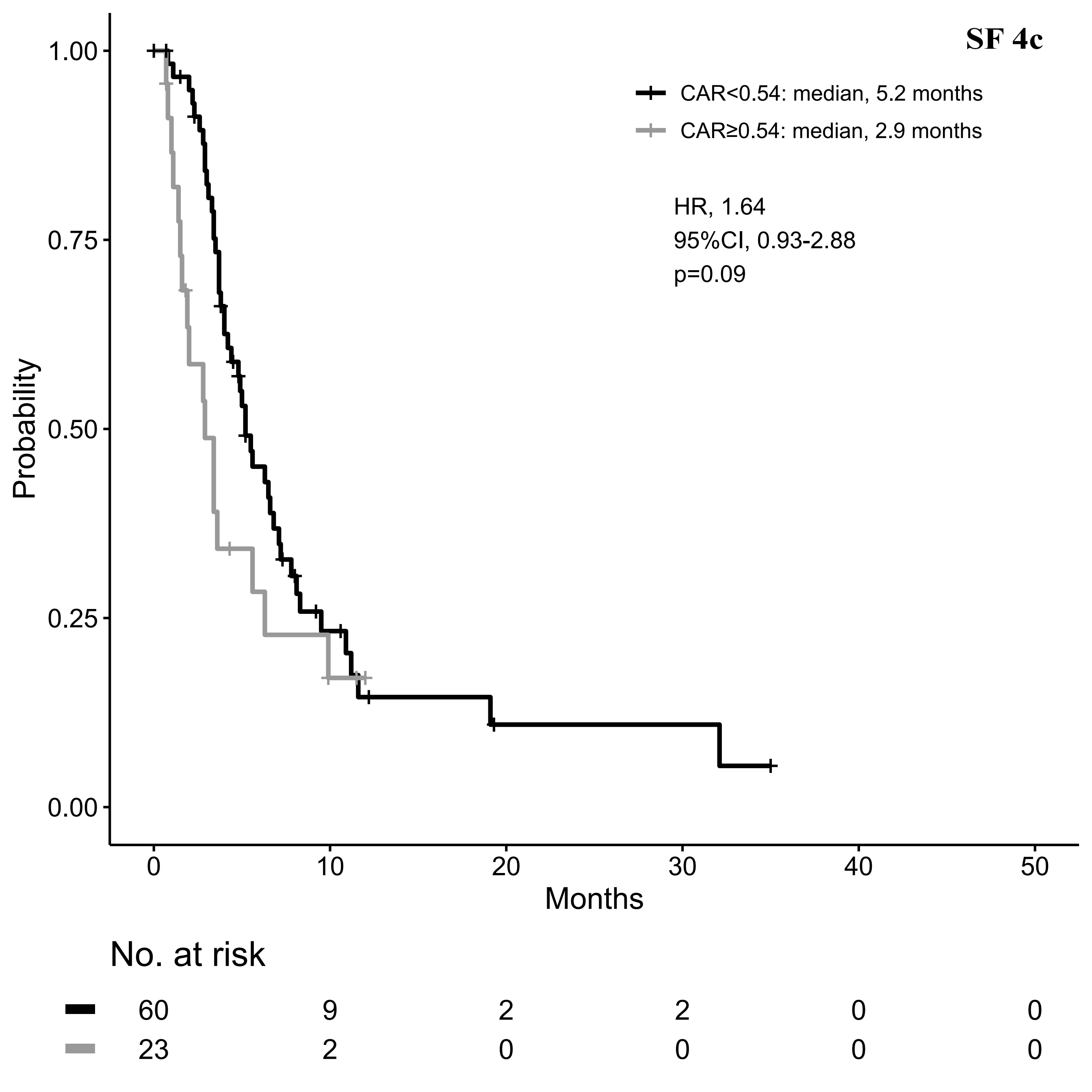
SF 4c
CAR<0.54: median, 5.2 months
CAR≥0.54: median, 2.9 months
HR, 1.64
95%CI, 0.93-2.88
p=0.09
Probability
1.00
0.75
0.50
0.25
0.00
0
10
20
30
40
50
Months
No. at risk
60 9 2 2 0 0
23 2 0 0 0 0

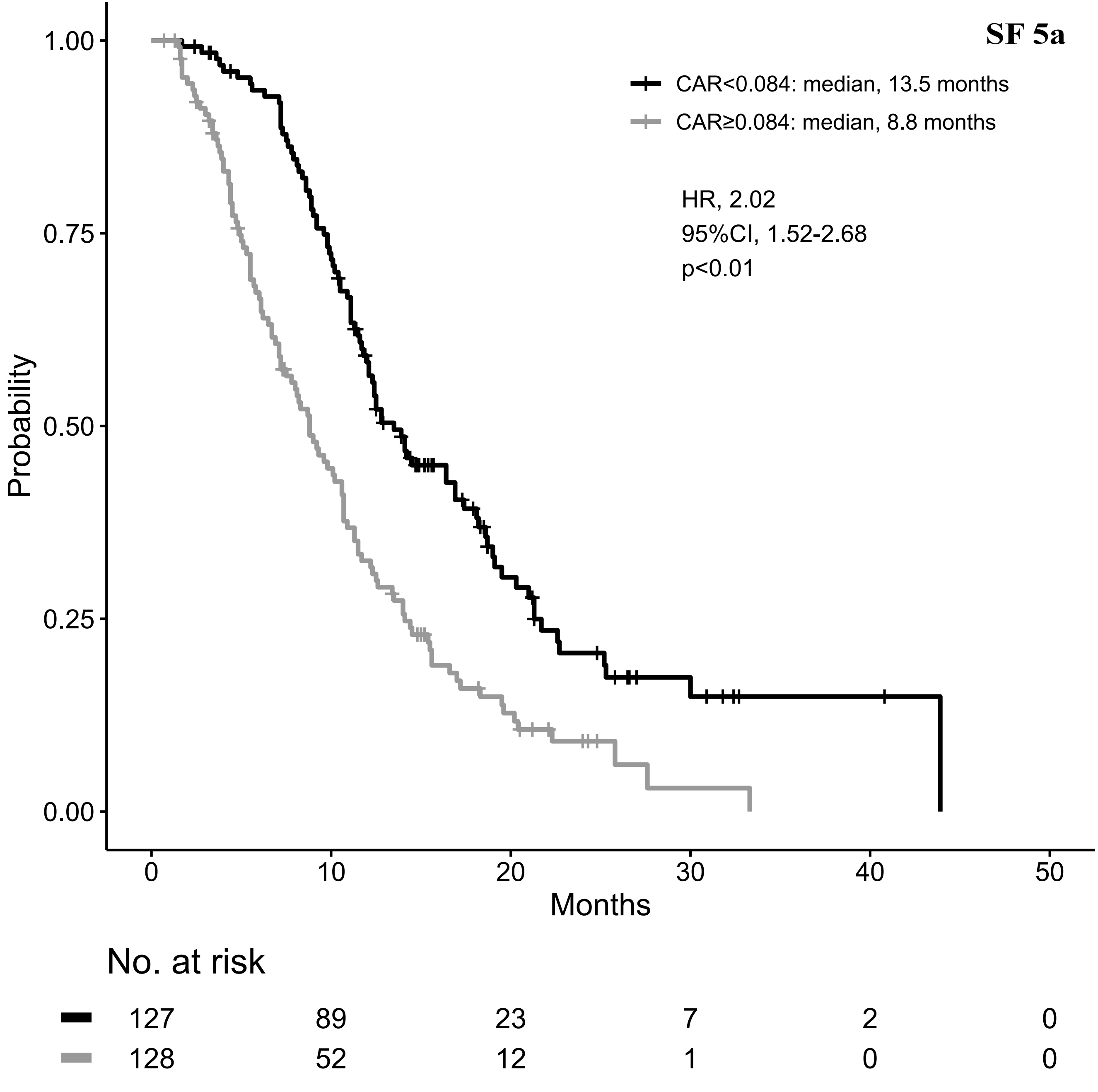
SF 5a
CAR<0.084: median, 13.5 months
CAR≥0.084: median, 8.8 months
HR, 2.02
95%CI, 1.52-2.68
p<0.01
Probability
1.00
0.75
0.50
0.25
0.00
0
10
20
30
40
50
Months
No. at risk
127 89 23 7 2 0
128 52 12 1 0 0

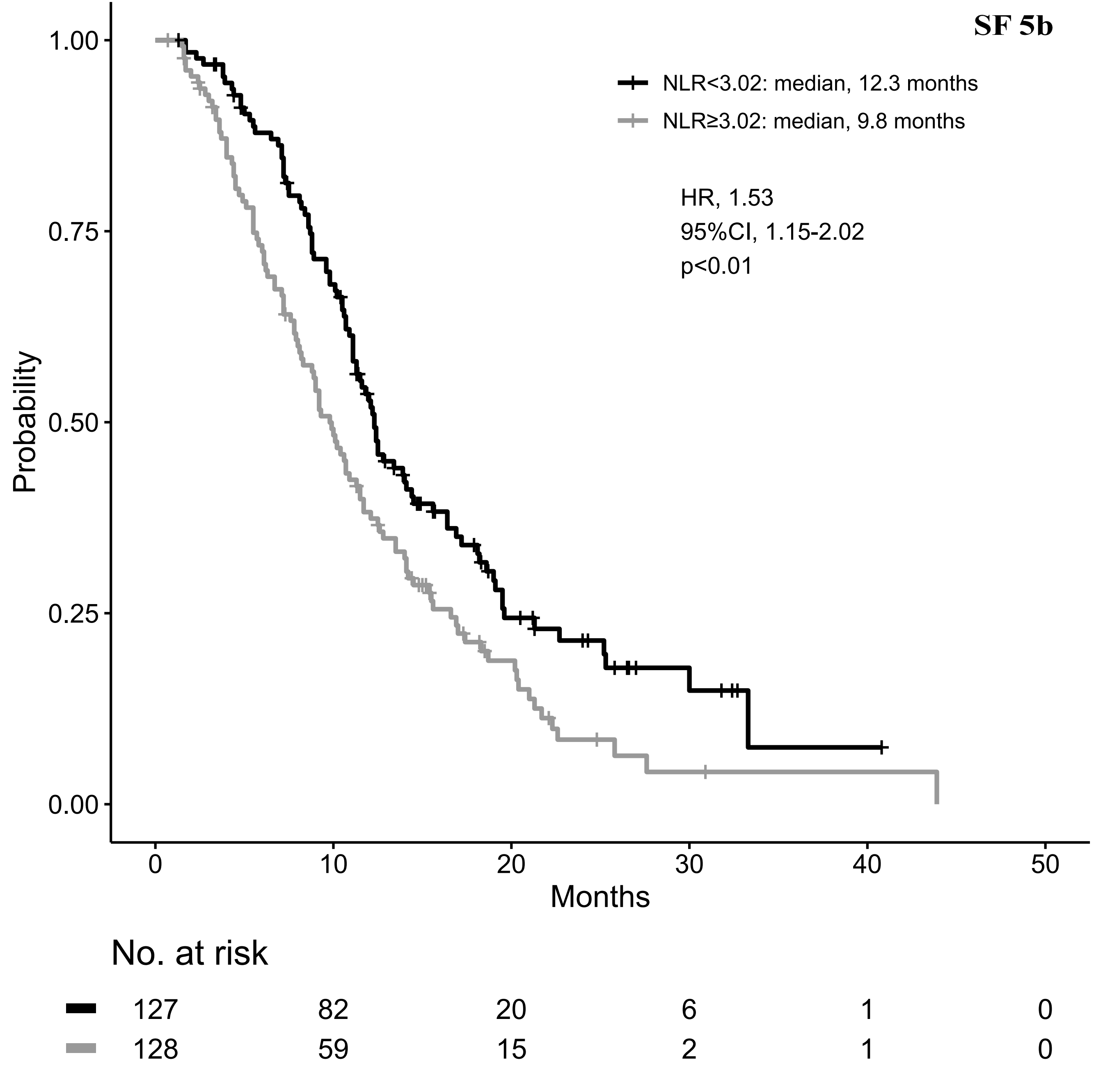
SF 5b
NLR<3.02: median, 12.3 months
NLR≥3.02: median, 9.8 months
HR, 1.53
95%CI, 1.15-2.02
p<0.01
Probability
1.00
0.75
0.50
0.25
0.00
0
10
20
30
40
50
Months
No. at risk
127 82 20 6 1 0
128 59 15 2 1 0

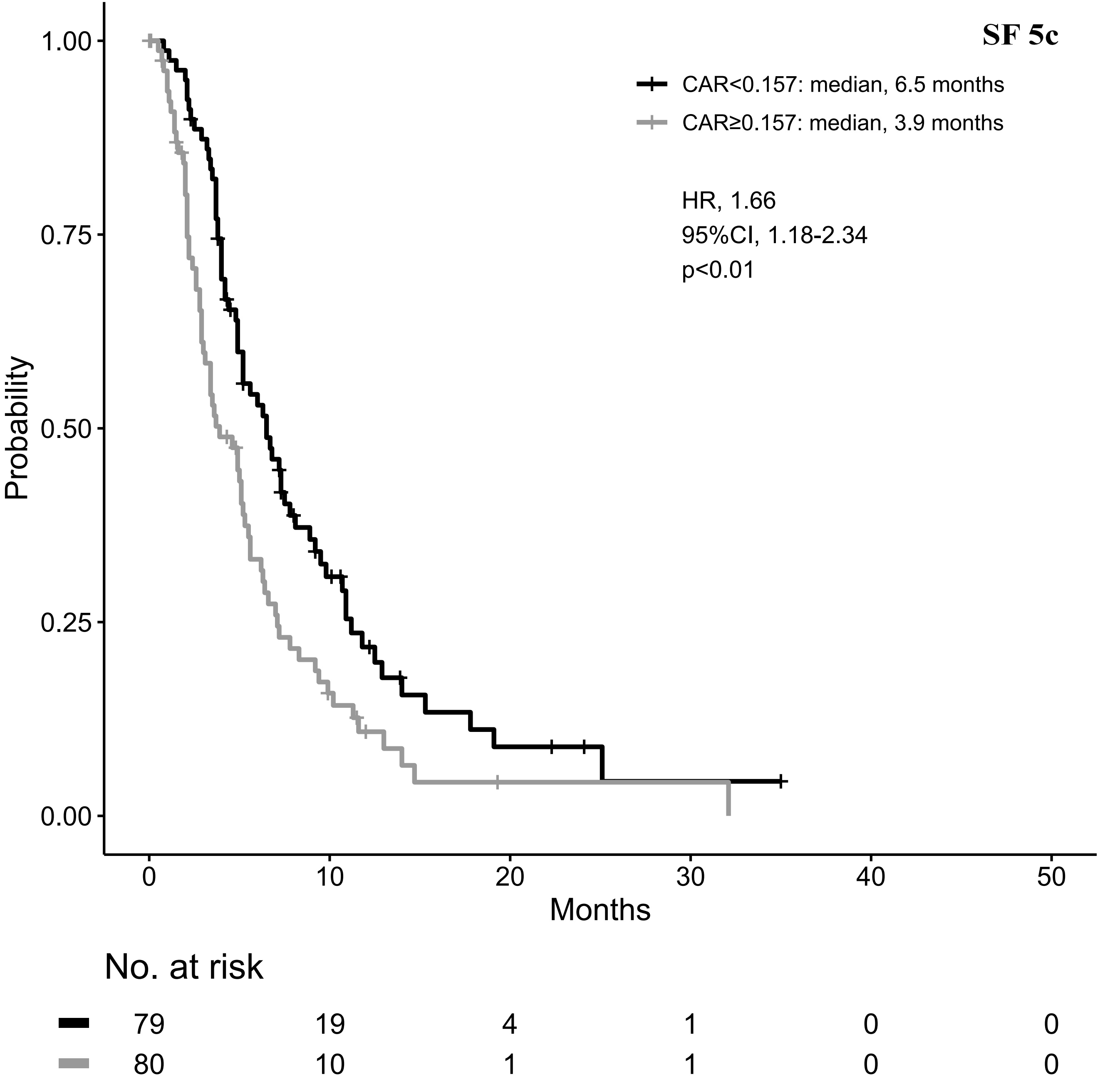

SF 5c
CAR<0.157: median, 6.5 months
CAR≥0.157: median, 3.9 months
HR, 1.66
95%CI, 1.18-2.34
p<0.01
Probability
1.00
0.75
0.50
0.25
0.00
0
10
20
30
40
50
Months
No. at risk
79 19 4 1 0 0
80 10 1 1 0 0

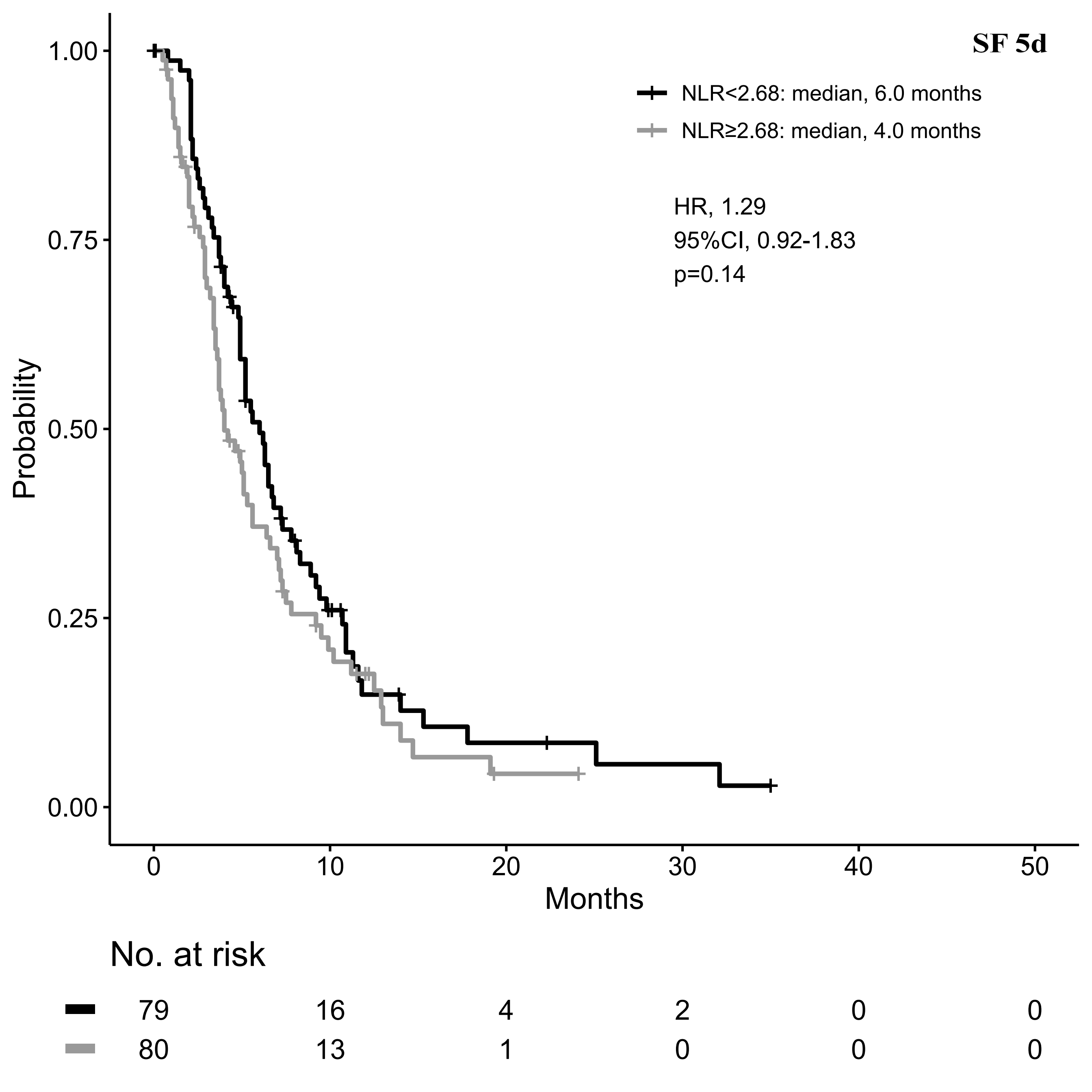
SF 5d
NLR<2.68: median, 6.0 months
NLR≥2.68: median, 4.0 months
HR, 1.29
95%CI, 0.92-1.83
p=0.14
Probability
1.00
0.75
0.50
0.25
0.00
0
10
20
30
40
50
Months
No. at risk
79 16 4 2 0 0
80 13 1 0 0 0

**Supplemental Table. Baseline characteristics of the CAR < 0.54 group and CAR ≥ 0.54 group**

| Characteristics | | Cohort 1 | | | Cohort 2 | | |
|---|---|---|---|---|---|---|---|
| | | CAR < 0.540 | CAR ≥ 0.540 | P | CAR < 0.540 | CAR ≥ 0.540 | P |
| | | n=193 (%) | n=62 (%) | | n=119 (%) | n=40 (%) | |
| Age, years | median (range) | 66 (29–86) | 63 (35–77) | < 0.01 | 65 (29–87) | 62 (36–80) | 0.24 |
| | ≥ 65 y.o. | 110 (57) | 20 (32) | < 0.01 | 62 (52) | 16 (40) | 0.19 |
| Sex, n (%) | male | 119 (62) | 39 (63) | 0.86 | 119 (62) | 39 (63) | 0.49 |
| ECOG PS, n (%) | 0 | 130 (67) | 31 (50) | < 0.01 | 54 (45) | 9 (23) | 0.047 |
| | 1 | 56 (29) | 22 (35) | | 52 (44) | 22 (55) | |
| | ≥ 2 | 7 (4) | 9 (15) | | 13 (11) | 8 (20) | |
| | unknown | 0 (0) | 0 (0) | | 0 (0) | 1 (3) | |
| Malignant history, n (%) | yes | 28 (15) | 6 (10) | 0.52 | 16 (13) | 3 (8) | 0.14 |
| Malignant family history, n (%) | yes | 59 (31) | 16 (26) | 0.77 | 41 (34) | 7 (18) | 0.04 |
| Previous tumor resection, n (%) | yes | 40 (21) | 4 (6) | 0.01 | 18 (15) | 2 (5) | 0.10 |
| Previous biliary drainage, n (%) | yes | 56 (29) | 9 (15) | 0.02 | 29 (24) | 10 (25) | 0.94 |
| Pancreatic tumor location, n (%) | head | 101 (52) | 22 (35) | 0.02 | 57 (48) | 18 (45) | 0.75 |
| | body/tail | 92 (48) | 40 (65) | | 62 (52) | 22 (55) | |
| Histology, n (%) | adenocarcinoma | 157 (81) | 55 (89) | 0.35 | 104 (87) | 34 (85) | 0.91 |
| | others | 9 (5) | 1 (2) | | 2 (2) | 1 (3) | |
| | unknown | 27 (14) | 6 (10) | | 13 (11) | 5 (13) | |
| Site of metastatic disease, n (%) | liver | 105 (54) | 49 (79) | < 0.01 | 73 (61) | 27 (68) | 0.49 |
| | peritoneum | 48 (25) | 14 (23) | 0.72 | 31 (26) | 6 (15) | 0.15 |
| | lung | 33 (17) | 6 (10) | 0.16 | 17 (14) | 5 (13) | 0.78 |
| Number of metastatic sites, n (%) | ≥ 2 | 66 (34) | 31 (50) | 0.03 | 46 (39) | 13 (33) | 0.49 |
| Ascites, n (%) | yes | 36 (19) | 20 (32) | 0.02 | 23 (19) | 6 (15) | 0.54 |
| First-line chemotherapy, n (%) | FFX | 76 (39) | 26 (42) | 0.72 | 60 (50) | 20 (50) | 0.96 |
| | GnP | 117 (61) | 36 (58) | | 59 (50) | 20 (50) | |
| White blood cell count, /μL | median (range) | 5590 (2400–11900) | 8400 (4350–23650) | < 0.01 | 4100 (1620–14200) | 7180 (1910–21910) | <0.01 |
| Neutrophil count, /μL | median (range) | 3620 (770–8680) | 5750 (2240–20810) | < 0.01 | 2500 (610–11720) | 5260 (1230–21250) | <0.01 |
| Lymphocyte count, /μL | median (range) | 1400 (440–3710) | 1170 (230–8640) | 0.02 | 1080 (260–2630) | 1100 (190–3030) | 0.95 |
| Platelet count, $\times 10^4$/μL | median (range) | 19.9 (6.7–46.5) | 23.3 (11.6–56.1) | < 0.01 | 17.6 (6.2–50.8) | 20.5 (2.4–79.8) | 0.07 |
| Albumin concentration, g/dL | median (range) | 4.0 (2.5–4.8) | 3.34 (2.2–4.5) | < 0.01 | 3.7 (2.2–4.6) | 3.3 (1.5–4.1) | < 0.01 |
| CRP, mg/dL | median (range) | 0.17 (0.01–2.12) | 4.28 (1.73–17.0) | < 0.01 | 0.27 (0.01–2.00) | 3.67 (2.00–16.33) | < 0.01 |
| LDH, U/L | median (range) | 174 (74–808) | 203 (108–1320) | < 0.01 | 206 (84–713) | 225 (112–447) | 0.07 |
| CEA, ng/mL | median (range) | 4.7 (0.4–626.6) | 13.9 (0.6–369.8) | < 0.01 | 7.2 (1.3–391.6) | 10.8 (1.0–1379.3) | 0.23 |
| CA19-9, U/mL | median (range) | 659 (1–1242609) | 1821 (2–6554100) | 0.12 | 808 (1–115905) | 4111 (2–7200000) | 0.02 |
| NLR | median (range) | 2.68 (0.28–8.37) | 4.52 (1.25–36.88) | < 0.01 | 2.16 (0.37–30.00) | 4.70 (1.32–49.42) | < 0.01 |
| | ≥ 5.00, n (%) | 17 (9) | 29 (47) | < 0.01 | 15 (13) | 19 (48) | < 0.01 |
| PLR | median (range) | 137.3 (44.4–906.6) | 209.5 (16.1–1344.8) | < 0.01 | 160.8 (46.6–1270.0) | 178.4 (35.8–1043.1) | 0.23 |
| | ≥ 150.0, n (%) | 85 (44) | 43 (69) | < 0.01 | 64 (54) | 25 (63) | 0.34 |
| PNI | median (range) | 46 (29–60) | 39 (25–66) | < 0.01 | 43 (24–54) | 39 (19–51) | < 0.01 |
| | < 47, n (%) | 104 (54) | 53 (85) | < 0.01 | 95 (80) | 38 (95) | 0.03 |

| | | | | | | | |
|---|---|---|---|---|---|---|---|
| CAR | median (range) | 0.044 (0.002–0.523) | 1.222 (0.546–7.182) | < 0.01 | 0.074 (0.003–0.513) | 1.175 (0.562–6.387) | < 0.01 |
| | ≥ 0.540, n (%) | 0 (0) | 62 (100) | < 0.01 | 0 (0) | 40 (100) | < 0.01 |
| GPS, n (%) | 0 | 146 (76) | 0 (0) | < 0.01 | 80 (67) | 0 (0) | < 0.01 |
| | 1 | 36 (19) | 26 (42) | | 35 (29) | 13 (33) | |
| | 2 | 11 (6) | 36 (58) | | 4 (3) | 27 (68) | |
| PI, n (%) | 0 | 165 (85) | 0 (0) | < 0.01 | 100 (84) | 0 (0) | < 0.01 |
| | 1 | 28 (15) | 54 (87) | | 18 (15) | 31 (78) | |
| | 2 | 0 (0) | 8 (13) | | 1 (1) | 9 (23) | |

Abbreviations: ECOG PS, Eastern Cooperative Oncology Group performance status; FFX, FOLFIRINOX; GnP, gemcitabine plus nab-paclitaxel; CRP, C-reactive protein; LDH, lactate dehydrogenase; CEA, carcinoembryonic antigen; CA19-9, carbohydrate antigen 19-9; NLR, neutrophil-to-lymphocyte ratio; PLR, platelet-lymphocyte ratio; PNI, prognostic nutrition index; CAR, CRP-albumin ratio; GPS, Glasgow prognostic score; PI, prognostic index.
