# Supplementary material for: Author Correction: C-reactive protein/albumin ratio is the most significant inflammatory marker in unresectable pancreatic cancer treated with FOLFIRINOX or gemcitabine plus nab-paclitaxel
Source: Sci Rep. 2024 Mar 4;14:5311. doi: 10.1038/s41598-024-55673-7 (PMC10912623; doi:10.1038/s41598-024-55673-7)
Supplement: Supplementary file 1 — Supplementary Information. [file 41598_2024_55673_MOESM1_ESM.pdf]

Supplemental Figure legends

Supplemental Figure 1.

Kaplan-Meier curves for progression-free survival in the first-line chemotherapy patients. (a) CAR < 0.54 group versus CAR ≥ 0.54 group, (b) NLR < 5 group versus NLR ≥ 5 group, (c) PNI ≥ 47 group versus PNI < 47 group, (d) PLR < 150 group versus PLR ≥ 150 group, (e) GPS score of 0 versus score of 1 versus score of 2, (f) PI score of 0 versus score of 1 versus score of 2.

CAR C-reactive protein/albumin ratio, *NLR* neutrophil–lymphocyte ratio, *PNI* prognostic nutrition index, *PLR* platelet–lymphocyte ratio, *GPS* modified Glasgow prognostic score, *PI* prognostic index

Supplemental Figure 2.

Kaplan-Meier curves for overall survival for the FFX or GnP cases in first-line chemotherapy patients. CAR < 0.54 group versus CAR ≥ 0.54 group in the FFX (a) and GnP (b) cases, NLR < 5 group versus NLR ≥ 5 group in the FFX (c) and GnP (d) cases, PNI ≥ 47 group versus PNI < 47 group in the FFX (e) and GnP (f) cases, PLR < 150 group versus PLR ≥ 150 group in the FFX (g) and GnP (h) cases, GPS score of 0 versus score of 1 versus score of 2 in FFX (i) and GnP (j) cases, PI score of 0 versus score of 1 versus score of 2 in the FFX (k) and GnP (l) cases.

FFX FOLFIRINOX*, GnP* gemcitabine plus nab-paclitaxel, *CAR* C-reactive protein/albumin ratio, *NLR* neutrophil–lymphocyte ratio, *PNI* prognostic nutrition index, *PLR* platelet–lymphocyte ratio, *GPS* modified Glasgow prognostic score, *PI* prognostic index

Supplemental Figure 3.

Kaplan-Meier curves for progression-free survival in the second-line chemotherapy patients. (a) CAR < 0.54 group versus CAR ≥ 0.54 group, (b) NLR < 5 group versus NLR ≥ 5 group, (c) PNI ≥ 47 group versus PNI < 47 group, (d) PLR < 150 group versus PLR ≥ 150 group, (e) GPS score of 0 versus score

of 1 versus score of 2, (f) PI score of 0 versus score of 1 versus score of 2.

CAR C-reactive protein/albumin ratio, *NLR* neutrophil–lymphocyte ratio, *PNI* prognostic nutrition index, *PLR* platelet–lymphocyte ratio, *GPS* modified Glasgow prognostic score, *PI* prognostic index

Supplemental Figure 4.

Kaplan-Meier curves for overall survival for the FFX or GnP cases in second-line chemotherapy patients. CAR < 0.54 group versus CAR ≥ 0.54 group in the FFX (a), GnP (b) and other (c) cases.

FFX FOLFIRINOX, *GnP* gemcitabine plus nab-paclitaxel, *CAR* C-reactive protein/albumin ratio

Supplemental Figure 5.

Kaplan-Meier curves for overall survival in the first- and second-line chemotherapy patients. (a) CAR < 0.084 group versus CAR ≥ 0.084 group in first-line, (b) NLR < 3.02 group versus NLR ≥ 3.02 group in first-line, (c) CAR < 0.157 group versus CAR ≥ 0.157 group in second-line, (d) NLR < 2.68 group versus NLR ≥ 2.68 group in second-line.

CAR C-reactive protein/albumin ratio, *NLR* neutrophil–lymphocyte ratio

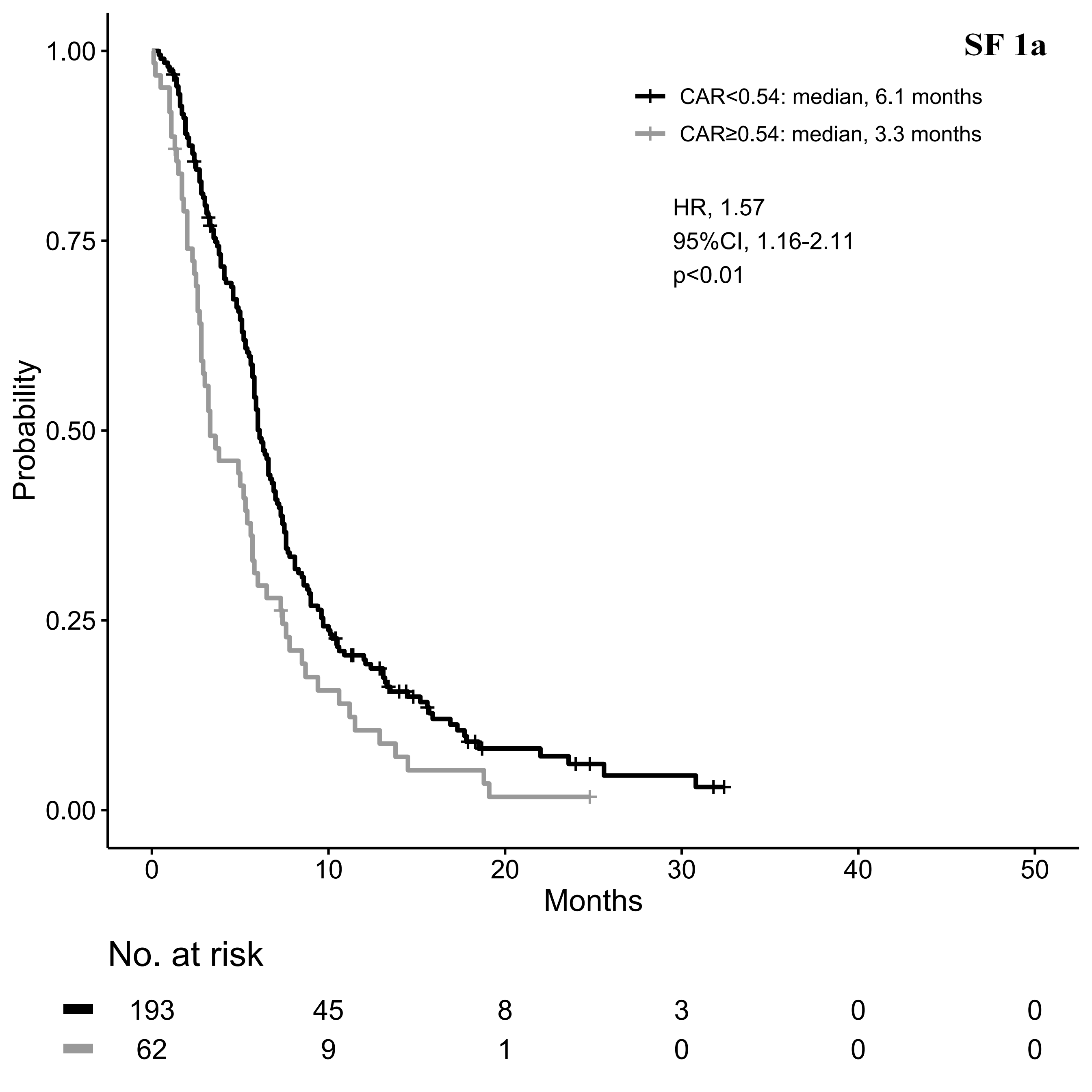
SF 1a
CAR<0.54: median, 6.1 months
CAR≥0.54: median, 3.3 months
HR, 1.57
95%CI, 1.16-2.11
p<0.01
Probability
1.00
0.75
0.50
0.25
0.00
0
10
20
30
40
50
Months
No. at risk
193 45 8 3 0 0
62 9 1 0 0 0

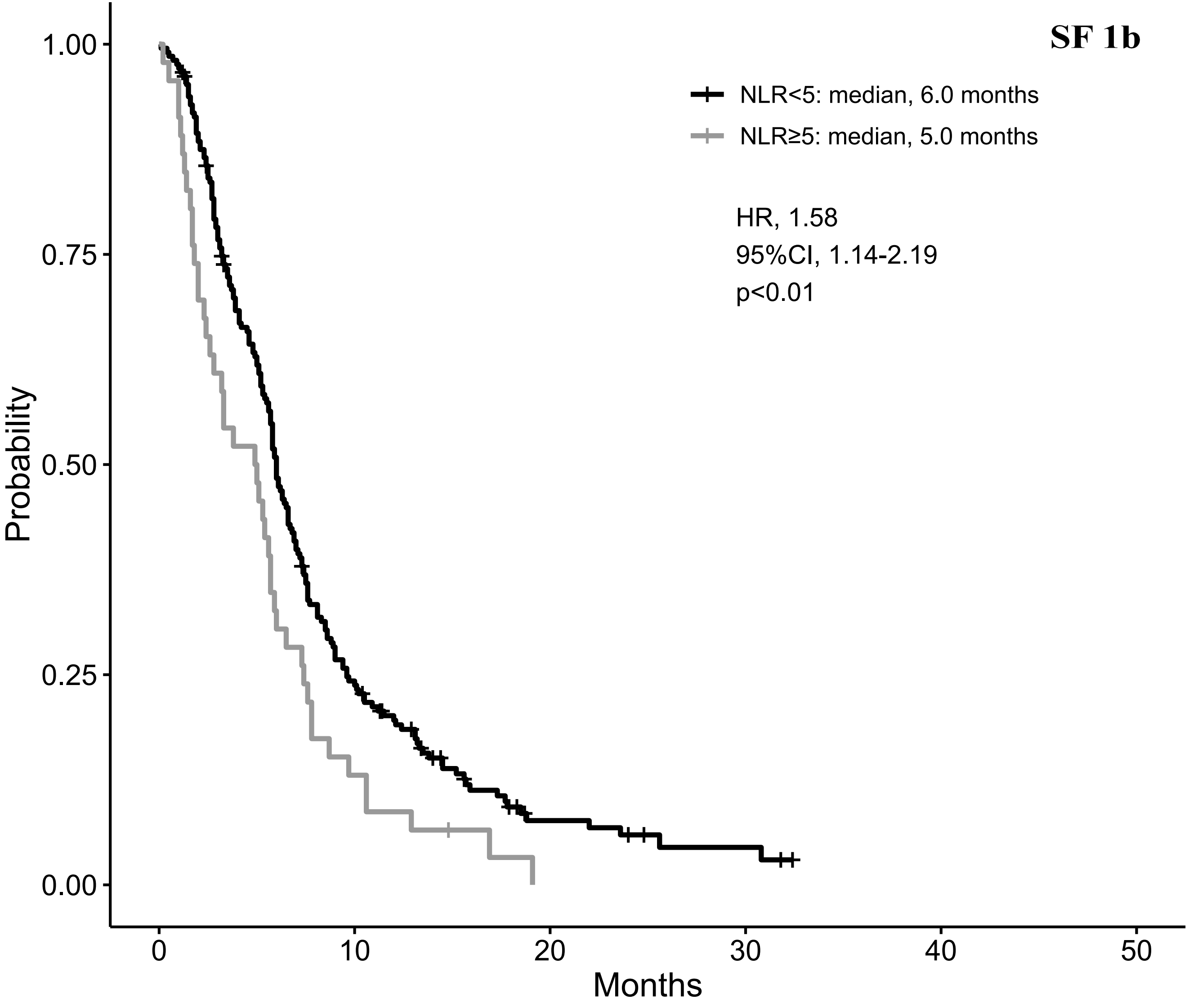

No. at risk

	0	10	20	30	40	50
NLR<5	209	48	9	3	0	0
NLR≥5	46	6	0	0	0	0

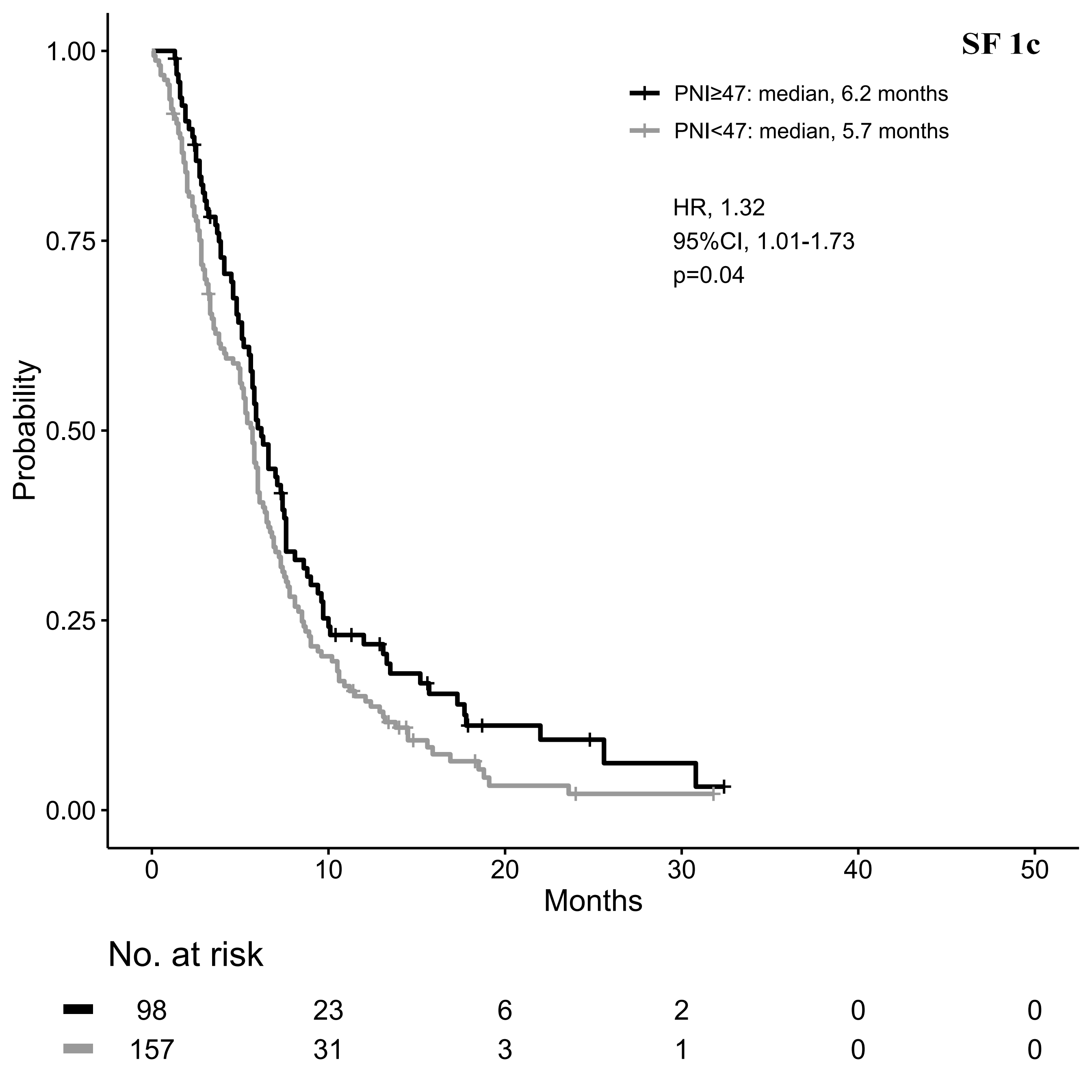

SF 1c
PNI≥47: median, 6.2 months
PNI<47: median, 5.7 months
HR, 1.32
95%CI, 1.01-1.73
p=0.04
Probability
1.00
0.75
0.50
0.25
0.00
0
10
20
30
40
50
Months
No. at risk
98 23 6 2 0 0
157 31 3 1 0 0

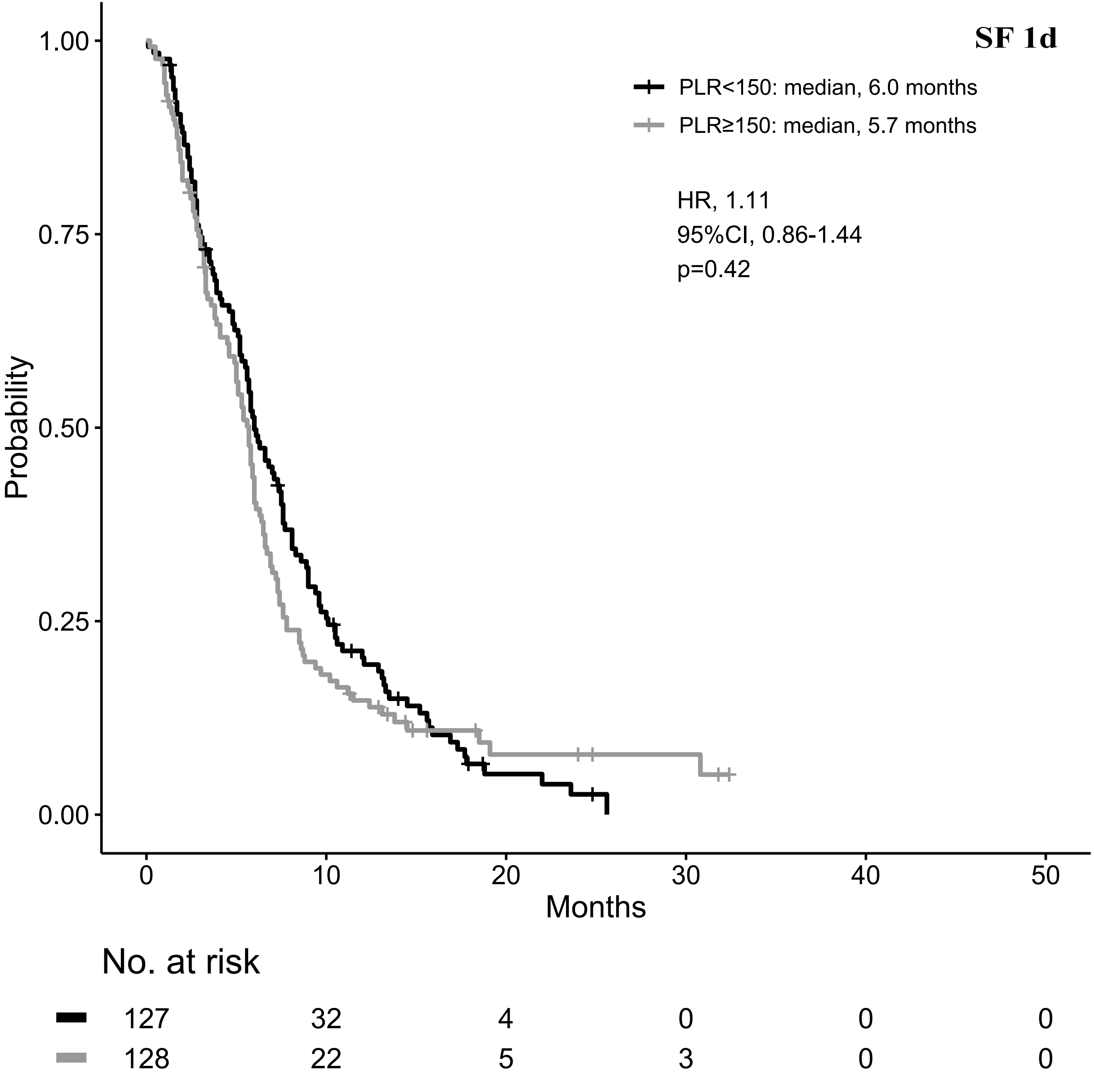
SF 1d
PLR<150: median, 6.0 months
PLR≥150: median, 5.7 months
HR, 1.11
95%CI, 0.86-1.44
p=0.42
Probability
1.00
0.75
0.50
0.25
0.00
0
10
20
30
40
50
Months
No. at risk
127 32 4 0 0 0
128 22 5 3 0 0

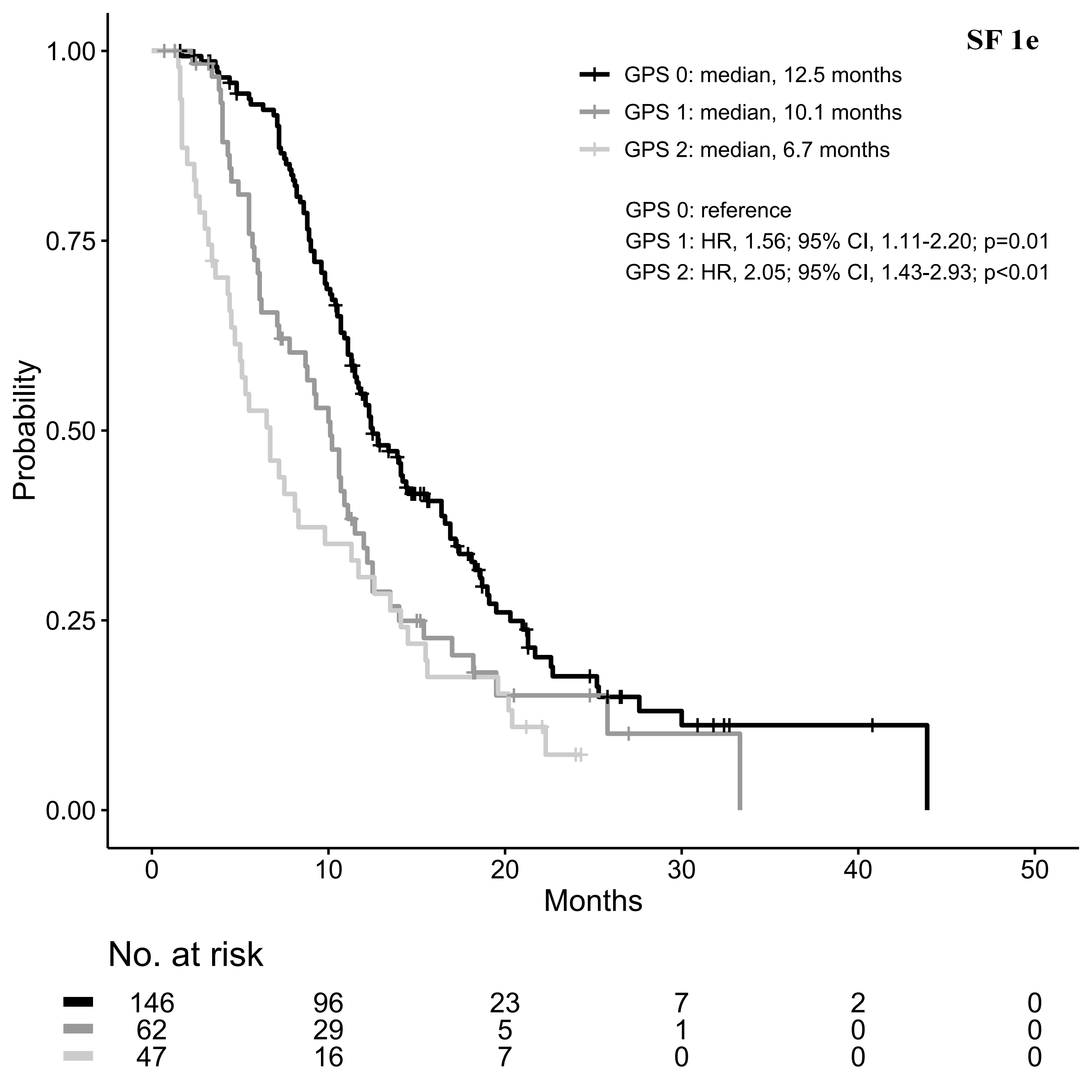
SF 1e
GPS 0: median, 12.5 months
GPS 1: median, 10.1 months
GPS 2: median, 6.7 months
GPS 0: reference
GPS 1: HR, 1.56; 95% CI, 1.11-2.20; p=0.01
GPS 2: HR, 2.05; 95% CI, 1.43-2.93; p<0.01
Probability
1.00
0.75
0.50
0.25
0.00
0
10
20
30
40
50
Months
No. at risk
146 96 23 7 2 0
62 29 5 1 0 0
47 16 7 0 0 0

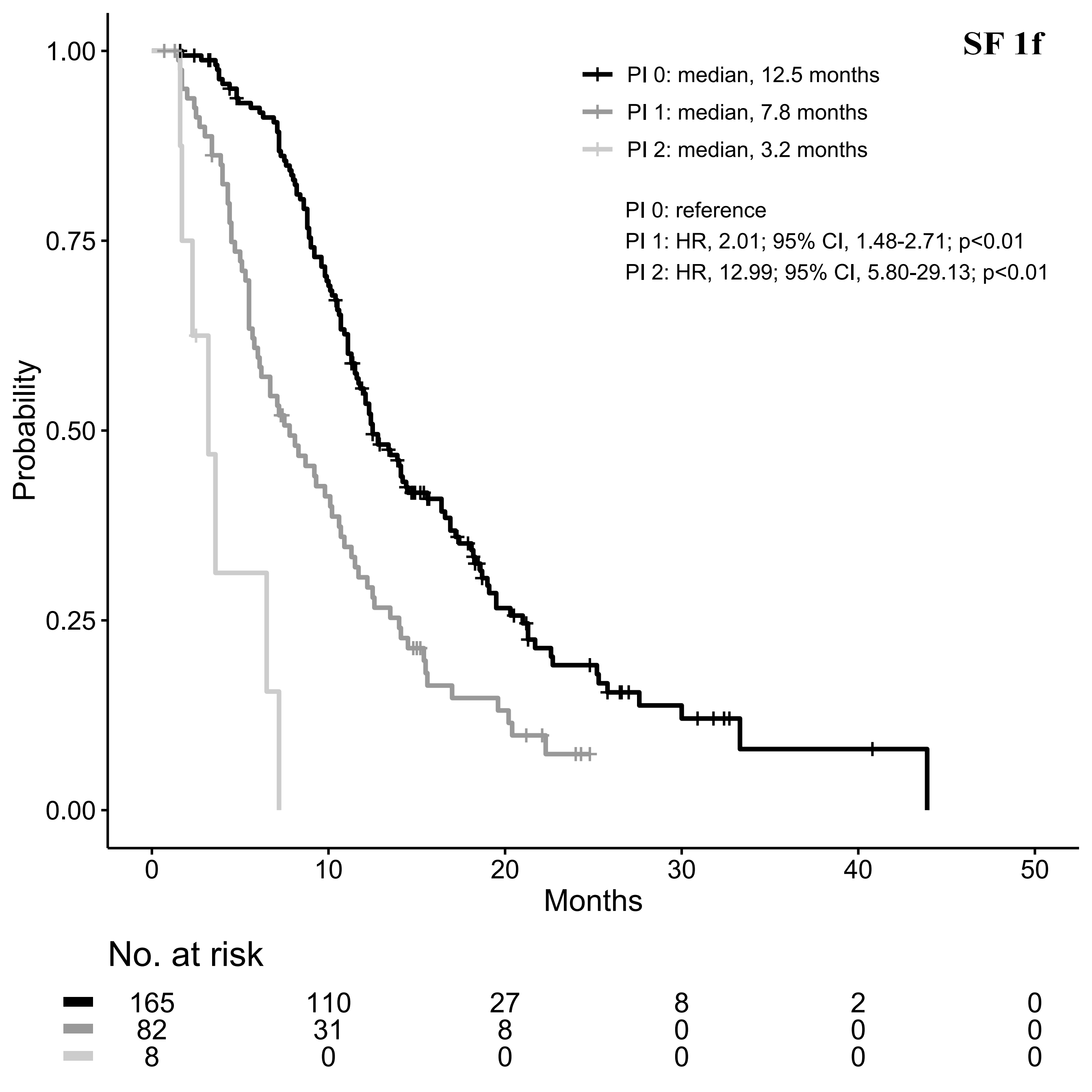
SF 1f
PI 0: median, 12.5 months
PI 1: median, 7.8 months
PI 2: median, 3.2 months
PI 0: reference
PI 1: HR, 2.01; 95% CI, 1.48-2.71; p<0.01
PI 2: HR, 12.99; 95% CI, 5.80-29.13; p<0.01
Probability
1.00
0.75
0.50
0.25
0.00
0
10
20
30
40
50
Months
No. at risk
165 110 27 8 2 0
82 31 8 0 0 0
8 0 0 0 0 0

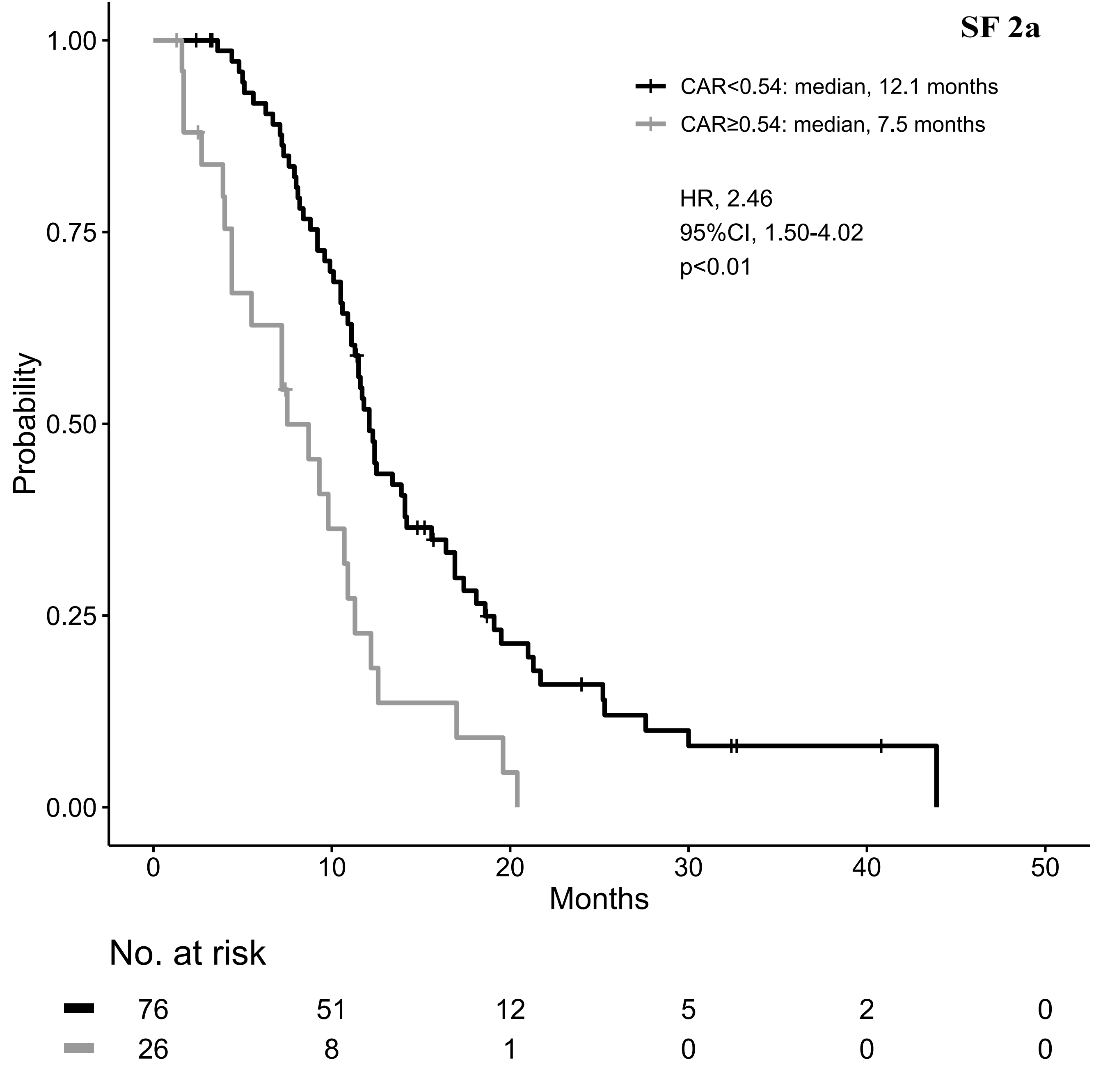
SF 2a
CAR<0.54: median, 12.1 months
CAR≥0.54: median, 7.5 months
HR, 2.46
95%CI, 1.50-4.02
p<0.01
Probability
1.00
0.75
0.50
0.25
0.00
0
10
20
30
40
50
Months
No. at risk
76 51 12 5 2 0
26 8 1 0 0 0

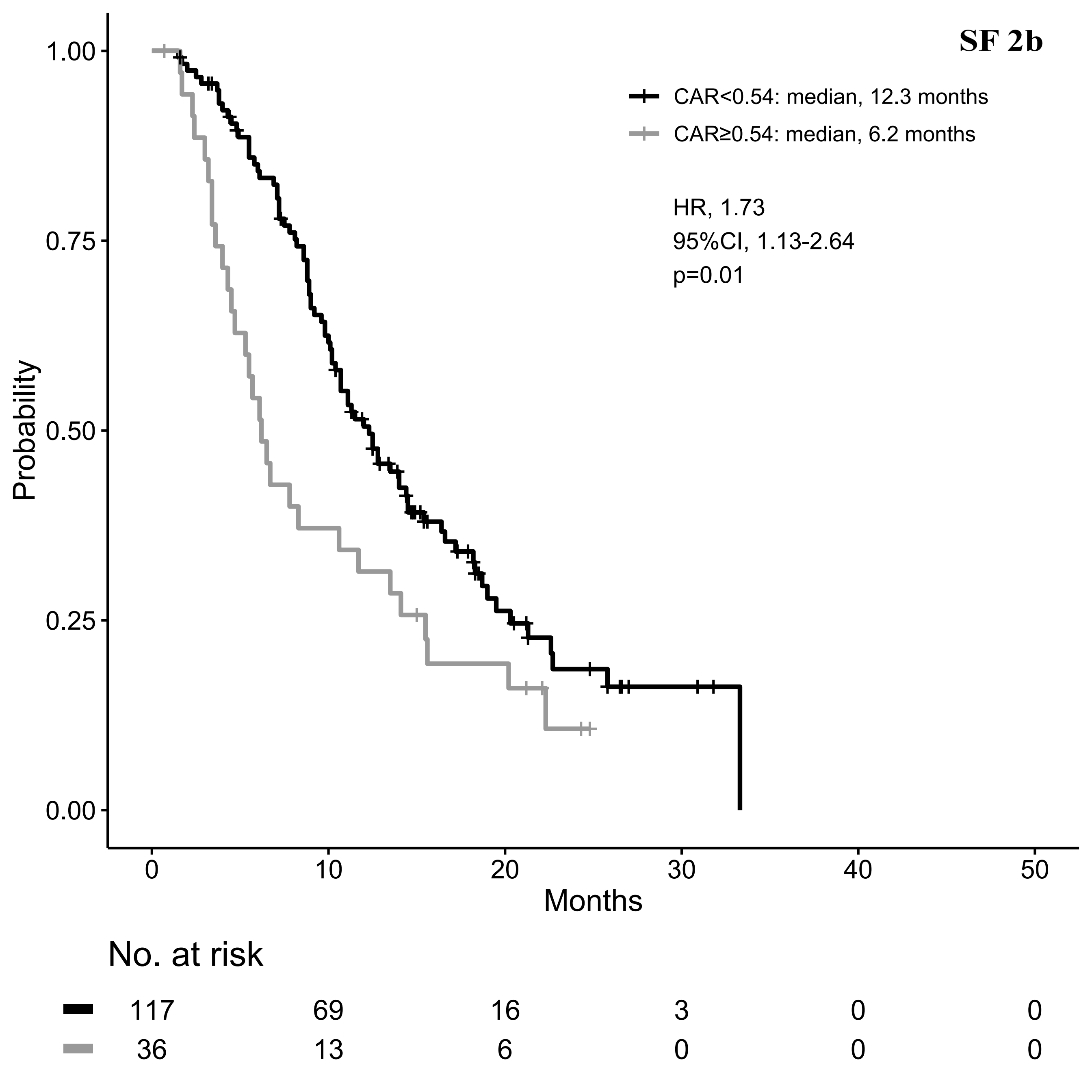
SF 2b
CAR<0.54: median, 12.3 months
CAR≥0.54: median, 6.2 months
HR, 1.73
95%CI, 1.13-2.64
p=0.01
Probability
1.00
0.75
0.50
0.25
0.00
0
10
20
30
40
50
Months
No. at risk
117
69
16
3
0
0
36
13
6
0
0
0

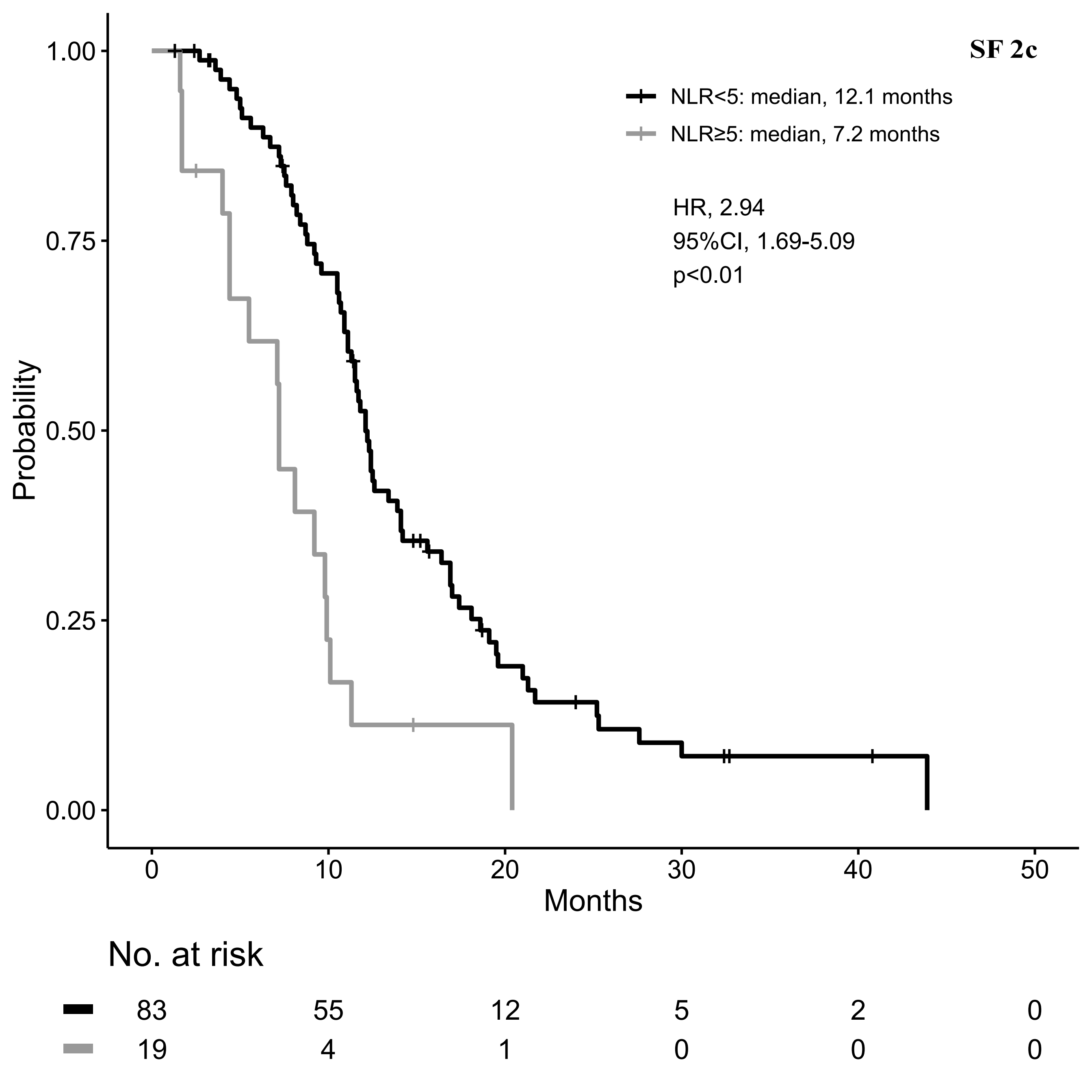

SF 2c
NLR<5: median, 12.1 months
NLR≥5: median, 7.2 months
HR, 2.94
95%CI, 1.69-5.09
p<0.01
Probability
1.00
0.75
0.50
0.25
0.00
0
10
20
30
40
50
Months
No. at risk
83 55 12 5 2 0
19 4 1 0 0 0

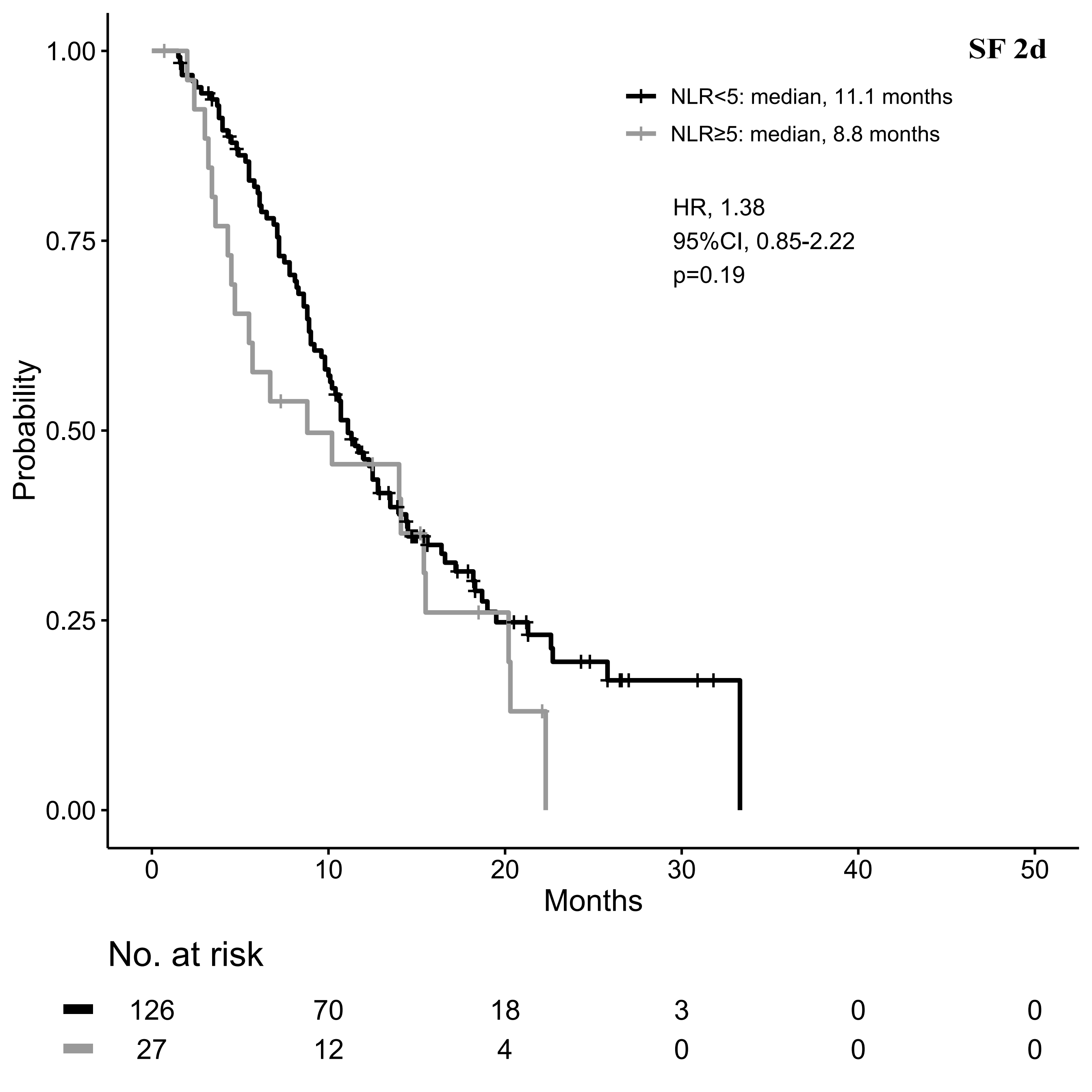

SF 2d
NLR<5: median, 11.1 months
NLR≥5: median, 8.8 months
HR, 1.38
95%CI, 0.85-2.22
p=0.19
Probability
1.00
0.75
0.50
0.25
0.00
0
10
20
30
40
50
Months
No. at risk
126 70 18 3 0 0
27 12 4 0 0 0

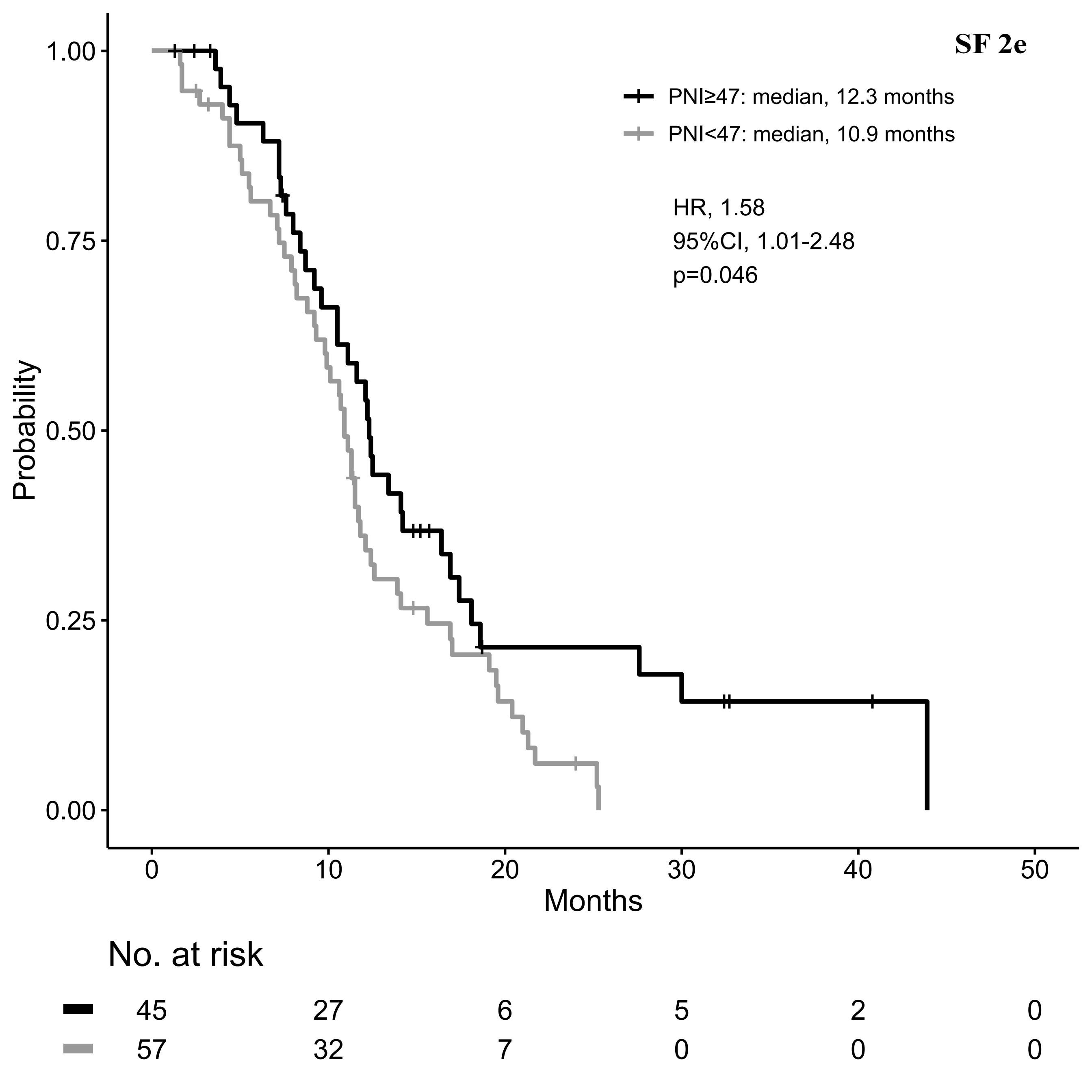

SF 2e
PNI≥47: median, 12.3 months
PNI<47: median, 10.9 months
HR, 1.58
95%CI, 1.01-2.48
p=0.046
Probability
1.00
0.75
0.50
0.25
0.00
0
10
20
30
40
50
Months
No. at risk
45 27 6 5 2 0
57 32 7 0 0 0

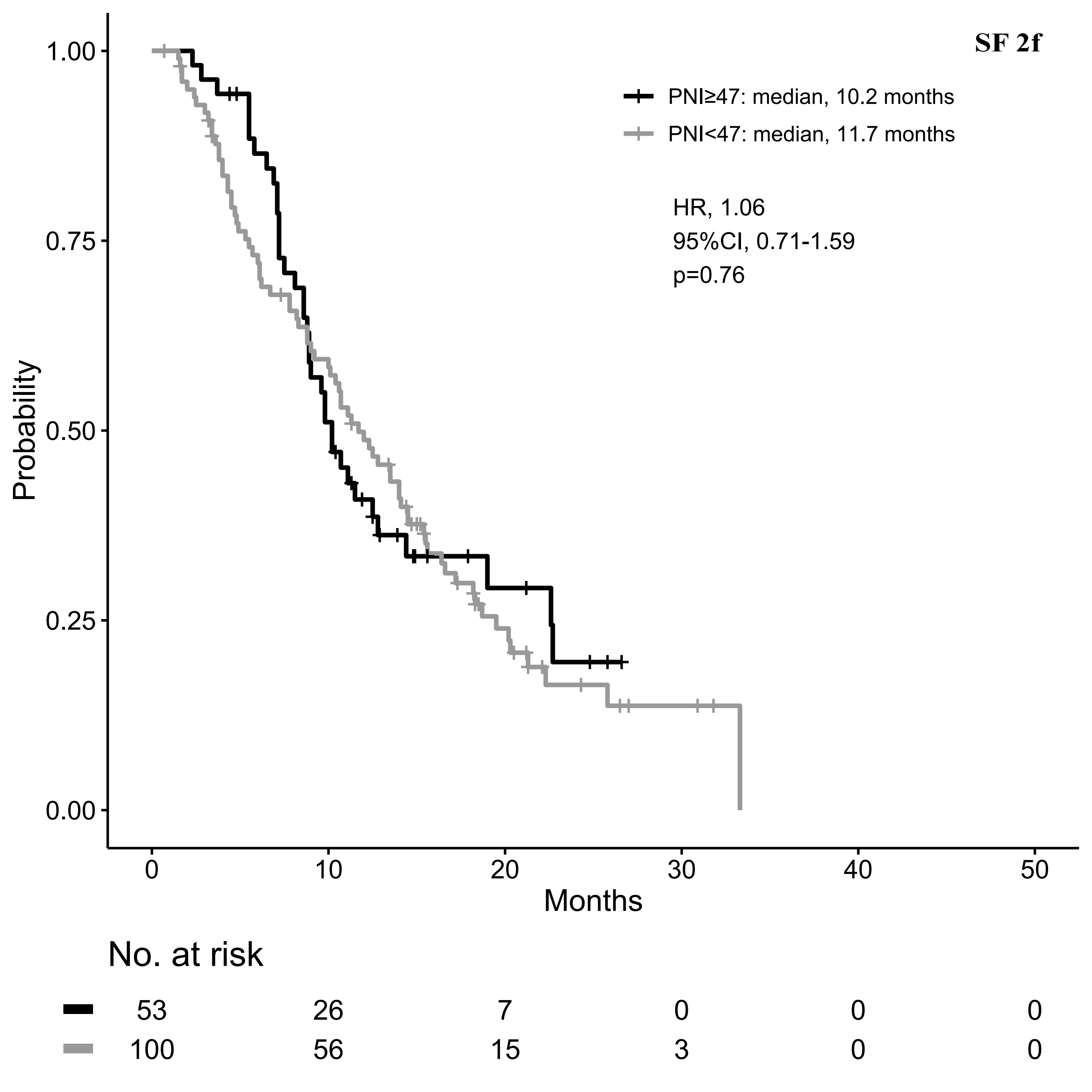
SF 2f
PNI≥47: median, 10.2 months
PNI<47: median, 11.7 months
HR, 1.06
95%CI, 0.71-1.59
p=0.76
Probability
1.00
0.75
0.50
0.25
0.00
0
10
20
30
40
50
Months
No. at risk
53 26 7 0 0 0
100 56 15 3 0 0

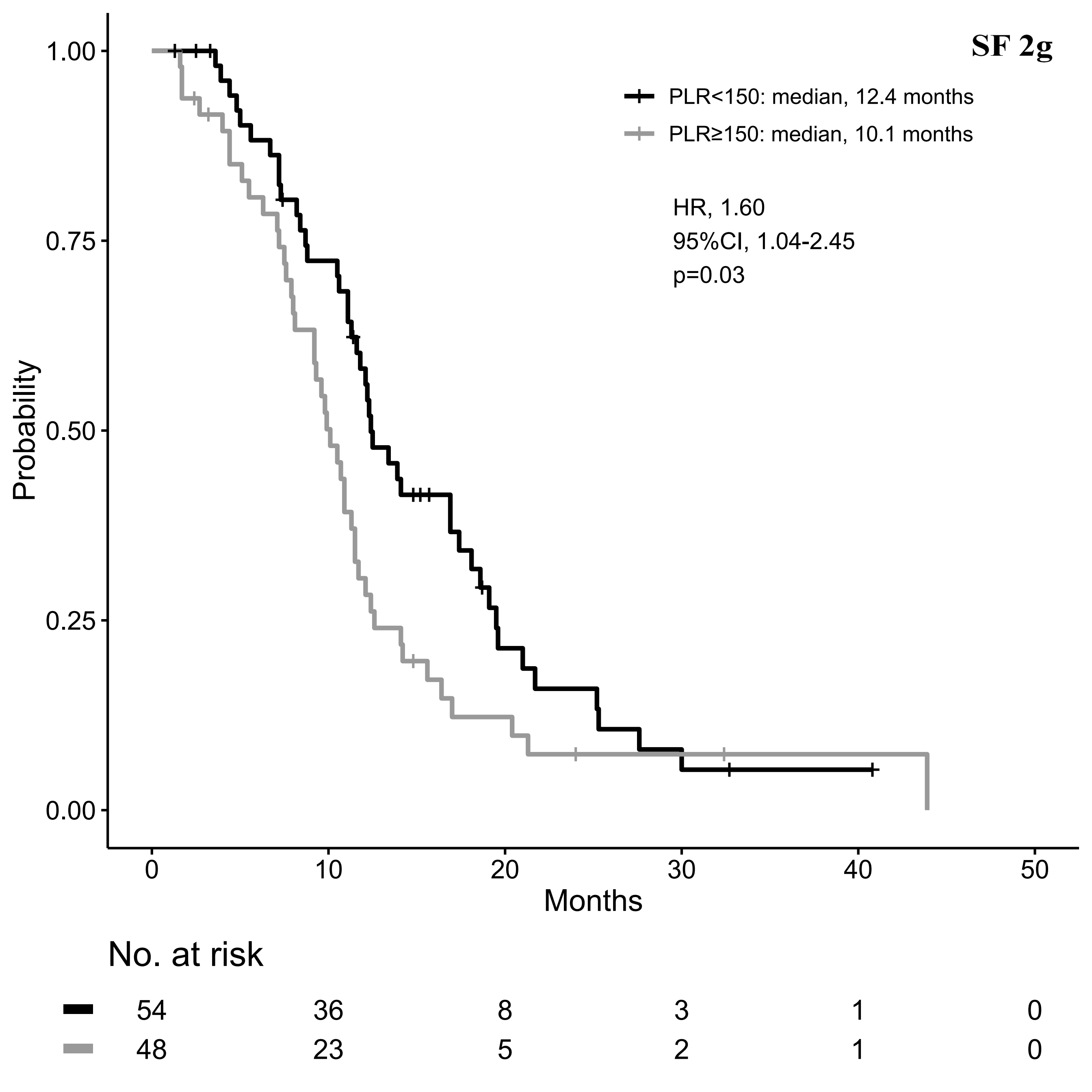
SF 2g
PLR<150: median, 12.4 months
PLR≥150: median, 10.1 months
HR, 1.60
95%CI, 1.04-2.45
p=0.03
Probability
1.00
0.75
0.50
0.25
0.00
0
10
20
30
40
50
Months
No. at risk
54 36 8 3 1 0
48 23 5 2 1 0

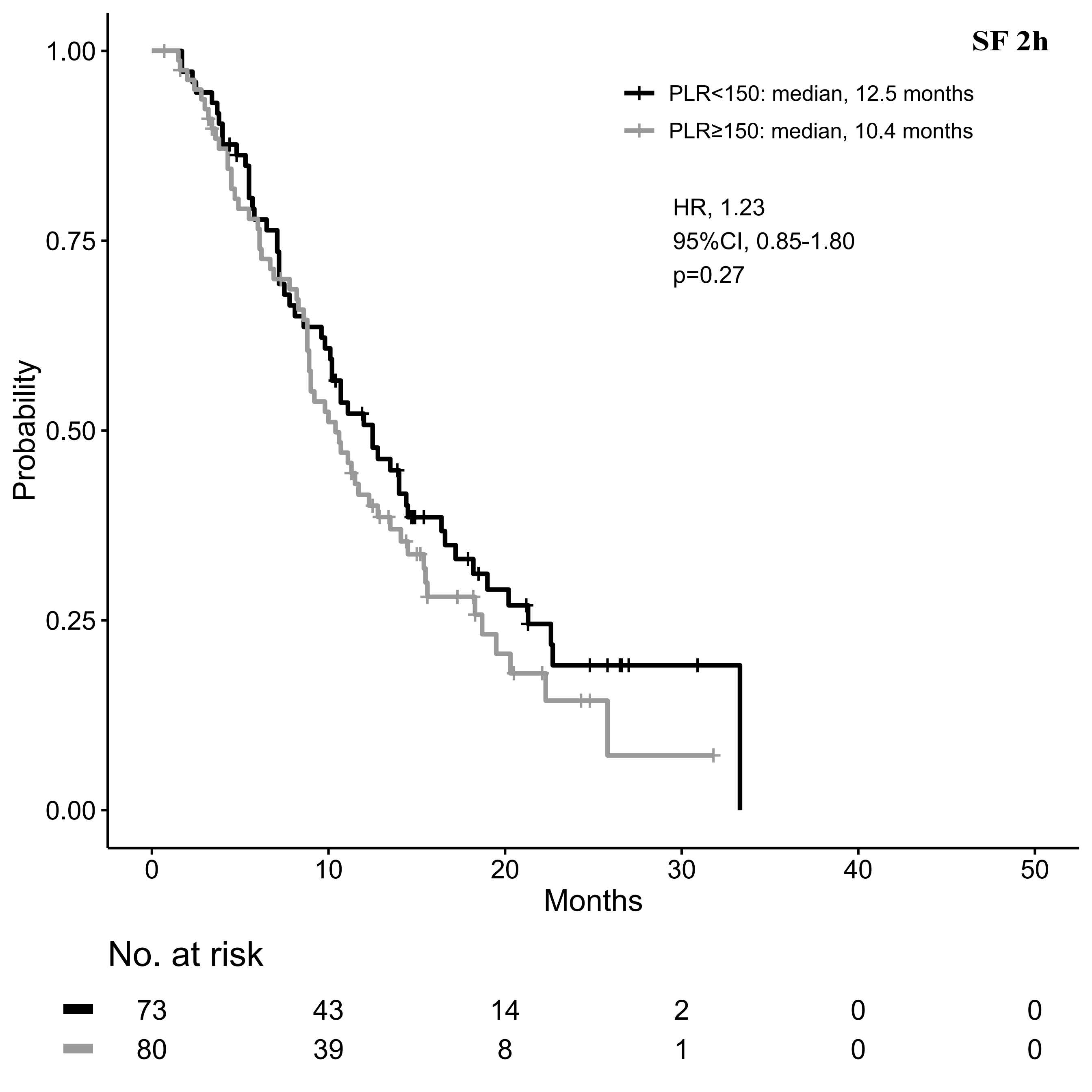
SF 2h
PLR<150: median, 12.5 months
PLR≥150: median, 10.4 months
HR, 1.23
95%CI, 0.85-1.80
p=0.27
Probability
1.00
0.75
0.50
0.25
0.00
0
10
20
30
40
50
Months
No. at risk
73 43 14 2 0 0
80 39 8 1 0 0

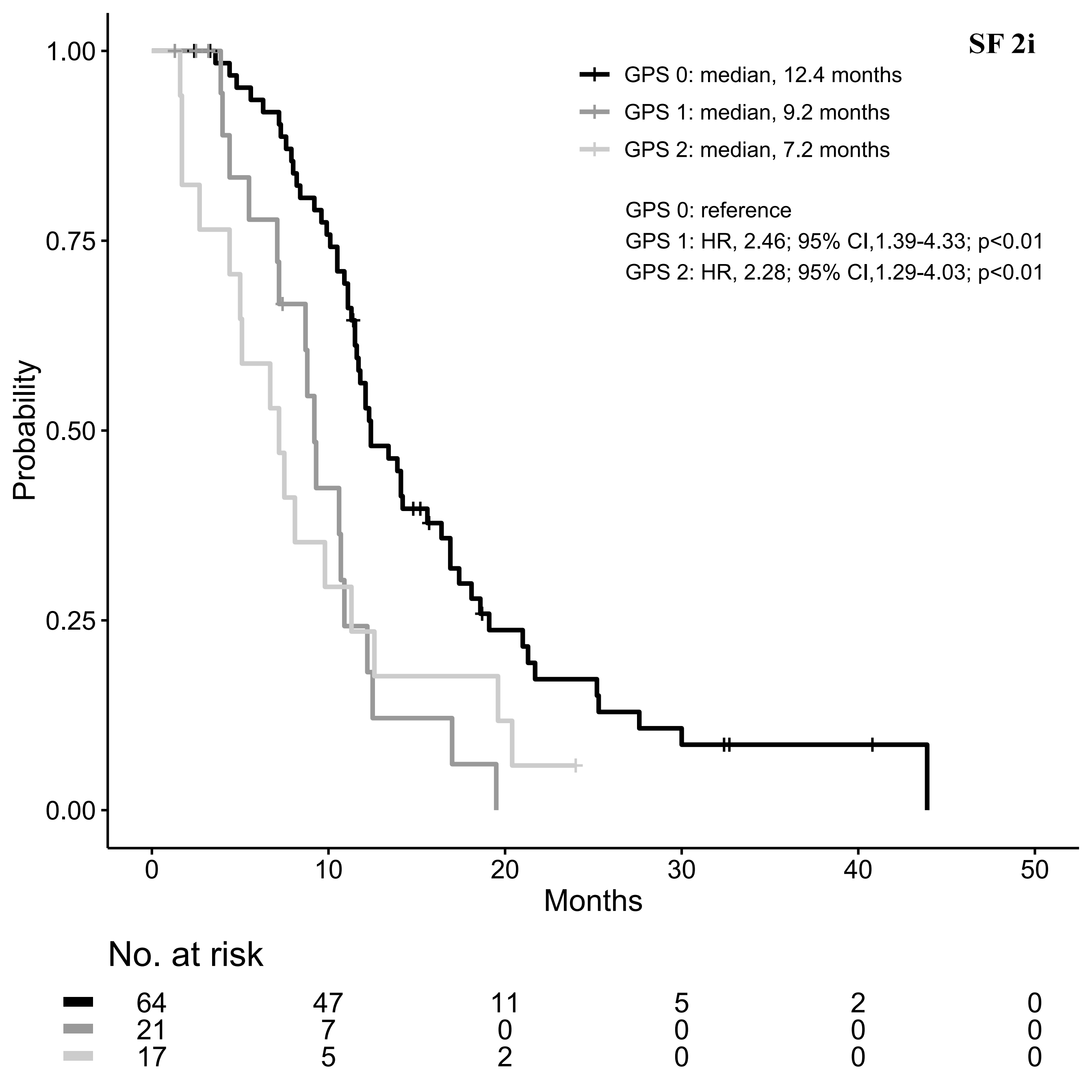
SF 2i
GPS 0: median, 12.4 months
GPS 1: median, 9.2 months
GPS 2: median, 7.2 months
GPS 0: reference
GPS 1: HR, 2.46; 95% CI,1.39-4.33; p<0.01
GPS 2: HR, 2.28; 95% CI,1.29-4.03; p<0.01
Probability
1.00
0.75
0.50
0.25
0.00
0
10
20
30
40
50
Months
No. at risk
64 47 11 5 2 0
21 7 0 0 0 0
17 5 2 0 0 0

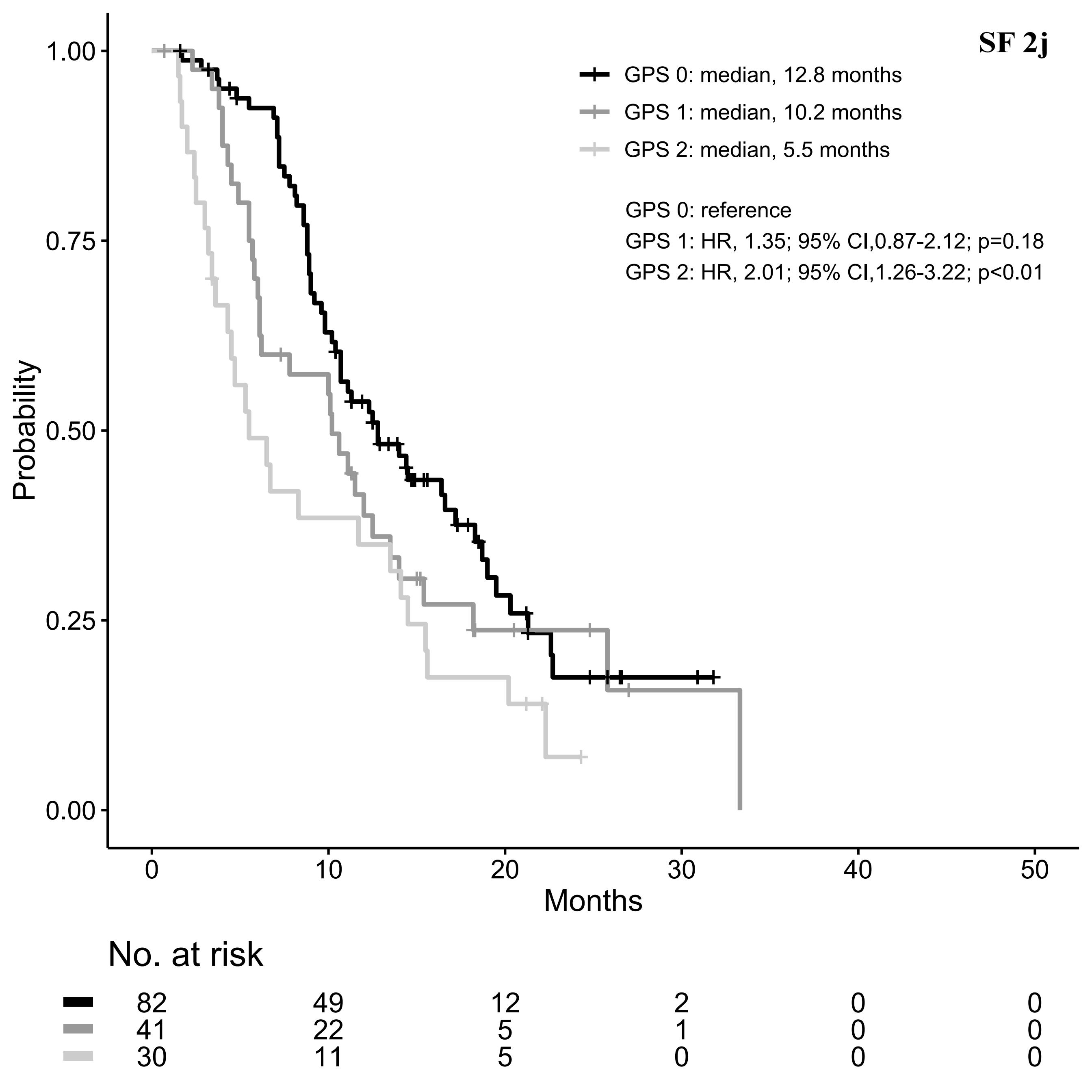
SF 2j
GPS 0: median, 12.8 months
GPS 1: median, 10.2 months
GPS 2: median, 5.5 months
GPS 0: reference
GPS 1: HR, 1.35; 95% CI,0.87-2.12; p=0.18
GPS 2: HR, 2.01; 95% CI,1.26-3.22; p<0.01
Probability
1.00
0.75
0.50
0.25
0.00
0
10
20
30
40
50
Months
No. at risk
82 49 12 2 0 0
41 22 5 1 0 0
30 11 5 0 0 0

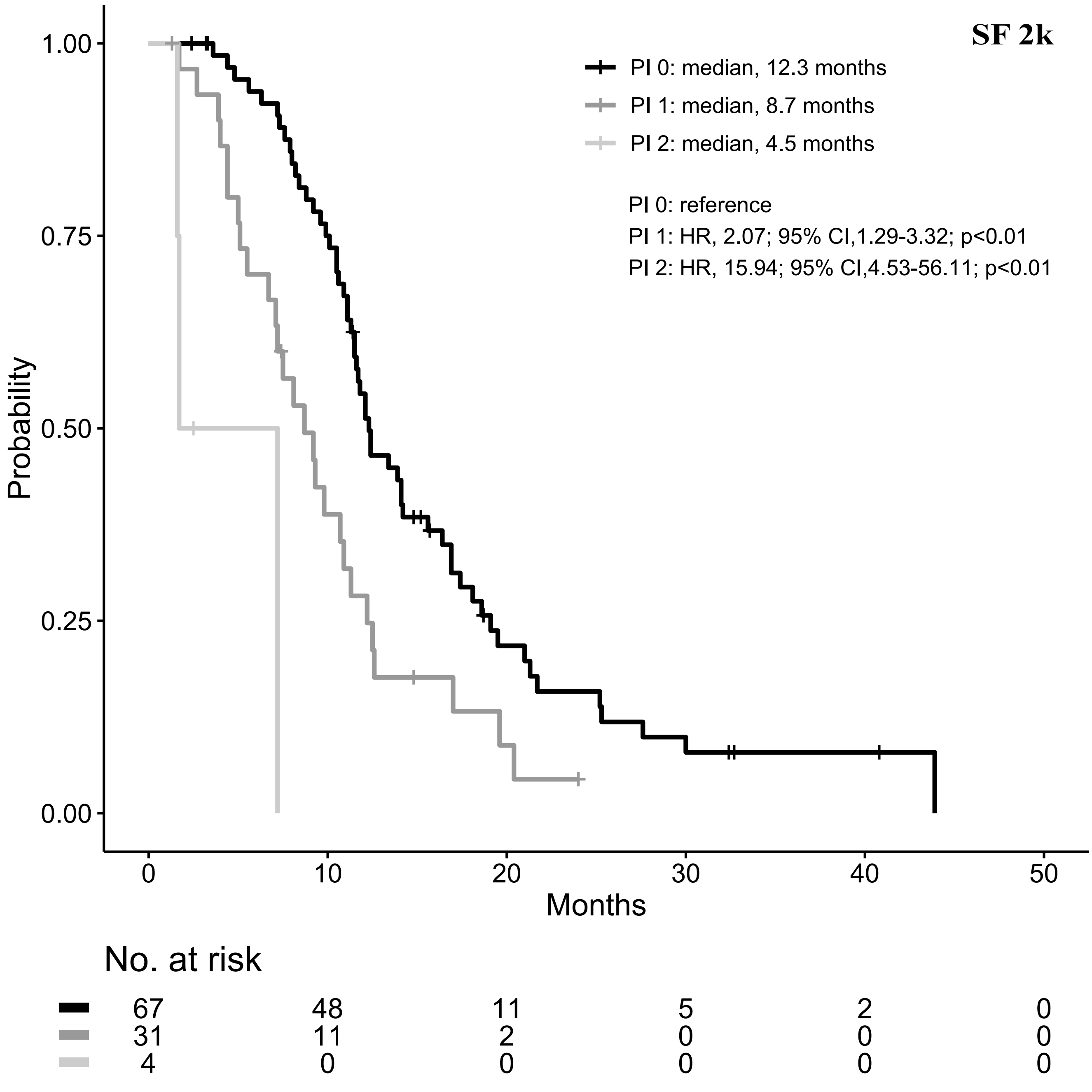
SF 2k
PI 0: median, 12.3 months
PI 1: median, 8.7 months
PI 2: median, 4.5 months
PI 0: reference
PI 1: HR, 2.07; 95% CI,1.29-3.32; p<0.01
PI 2: HR, 15.94; 95% CI,4.53-56.11; p<0.01
Probability
1.00
0.75
0.50
0.25
0.00
0
10
20
30
40
50
Months
No. at risk
67 48 11 5 2 0
31 11 2 0 0 0
4 0 0 0 0 0

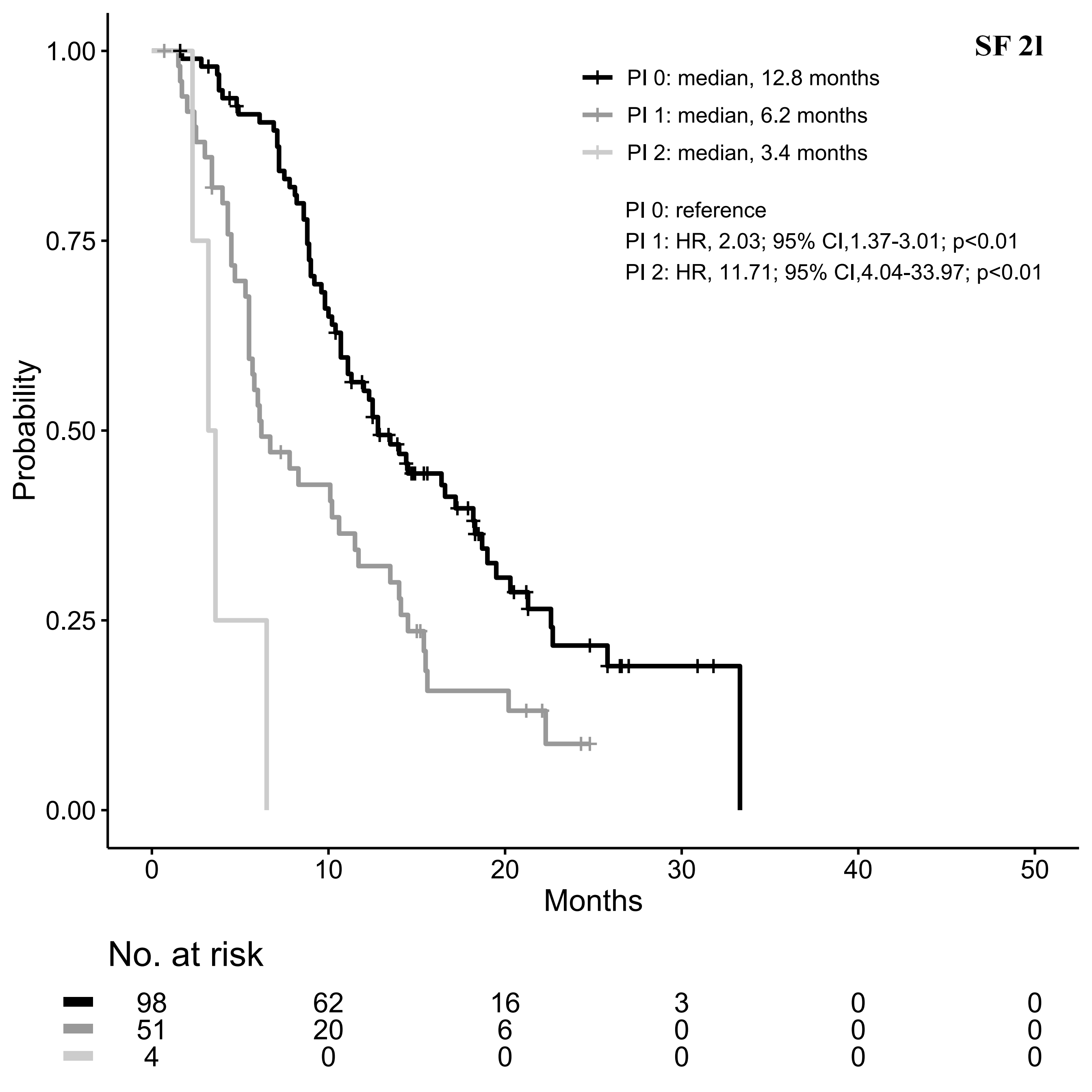
SF 2l
PI 0: median, 12.8 months
PI 1: median, 6.2 months
PI 2: median, 3.4 months
PI 0: reference
PI 1: HR, 2.03; 95% CI,1.37-3.01; p<0.01
PI 2: HR, 11.71; 95% CI,4.04-33.97; p<0.01
Probability
1.00
0.75
0.50
0.25
0.00
0
10
20
30
40
50
Months
No. at risk
98 62 16 3 0 0
51 20 6 0 0 0
4 0 0 0 0 0

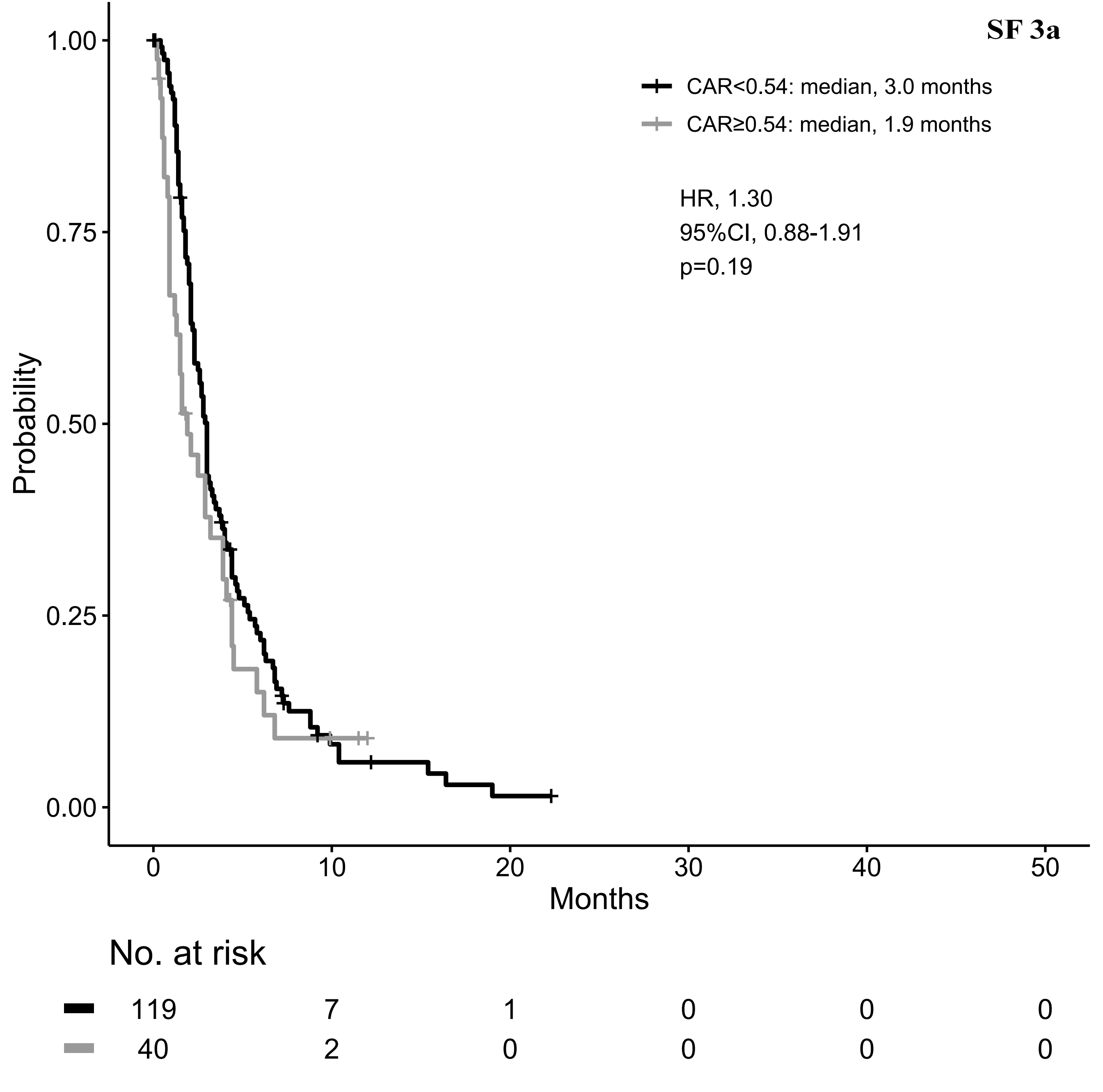
SF 3a
CAR<0.54: median, 3.0 months
CAR≥0.54: median, 1.9 months
HR, 1.30
95%CI, 0.88-1.91
p=0.19
Probability
1.00
0.75
0.50
0.25
0.00
0
10
20
30
40
50
Months
No. at risk
119 7 1 0 0 0
40 2 0 0 0 0

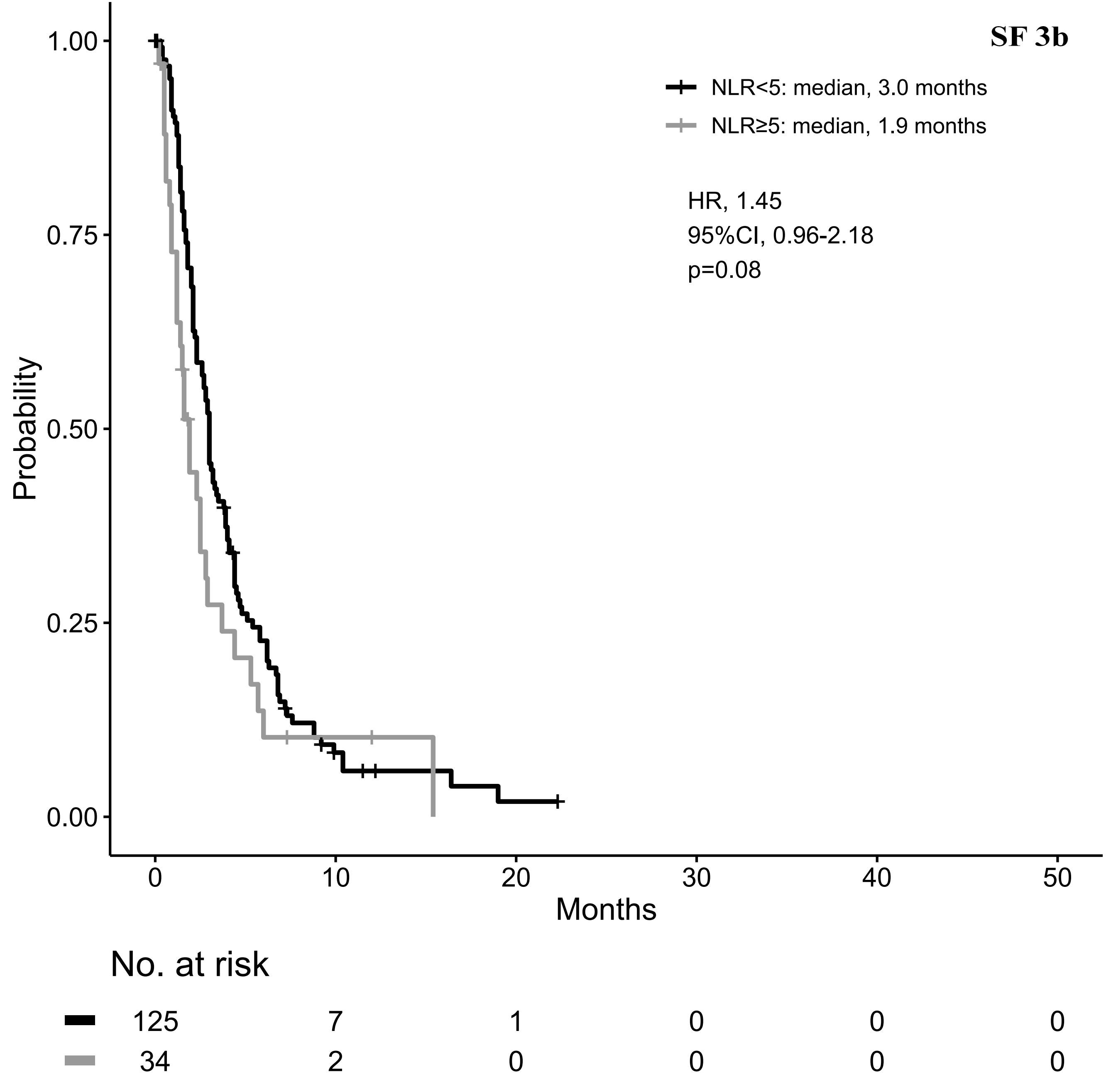
SF 3b
NLR<5: median, 3.0 months
NLR≥5: median, 1.9 months
HR, 1.45
95%CI, 0.96-2.18
p=0.08
Probability
1.00
0.75
0.50
0.25
0.00
0
10
20
30
40
50
Months
No. at risk
125 7 1 0 0 0
34 2 0 0 0 0

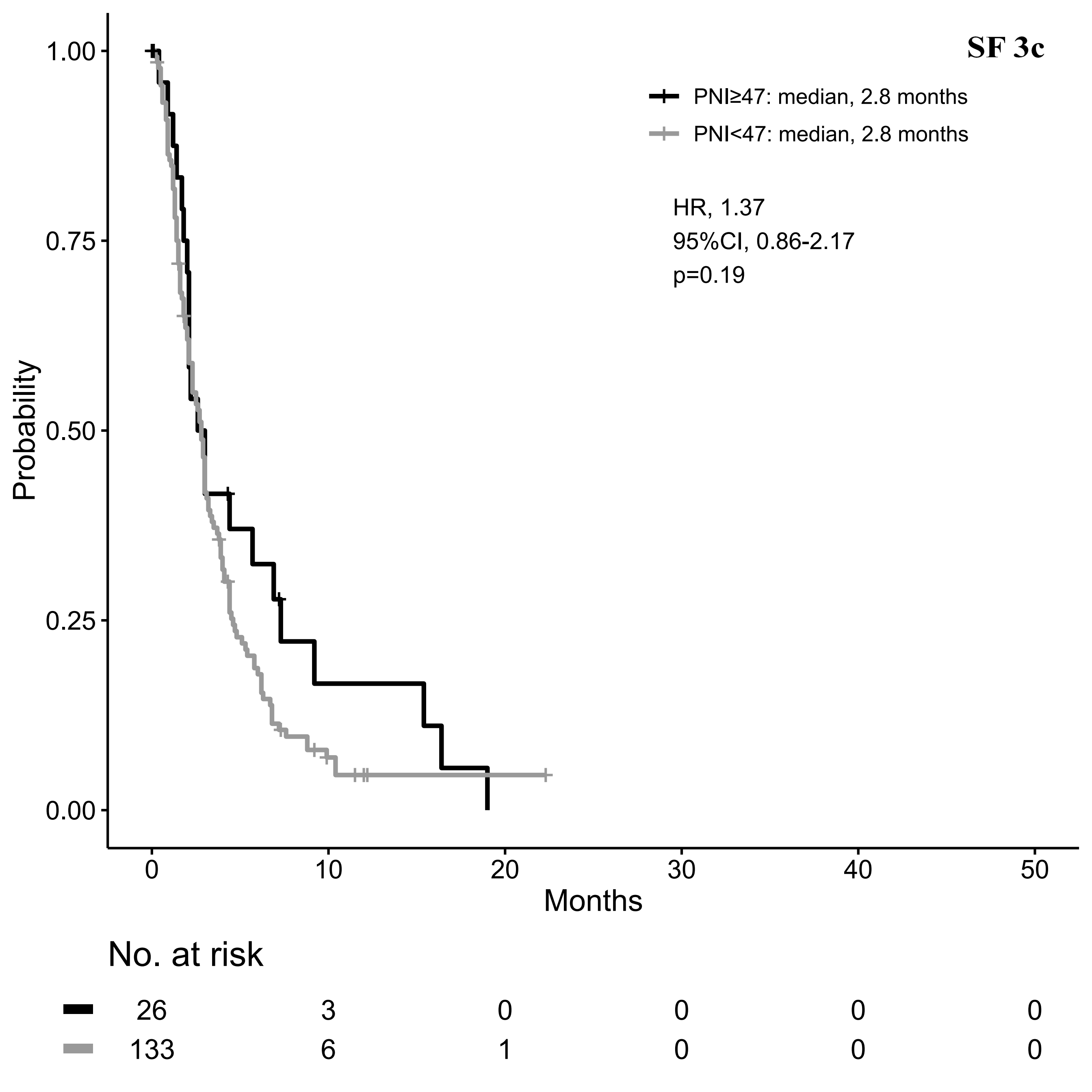

SF 3c
PNI≥47: median, 2.8 months
PNI<47: median, 2.8 months
HR, 1.37
95%CI, 0.86-2.17
p=0.19
Probability
1.00
0.75
0.50
0.25
0.00
0
10
20
30
40
50
Months
No. at risk
26 3 0 0 0 0
133 6 1 0 0 0

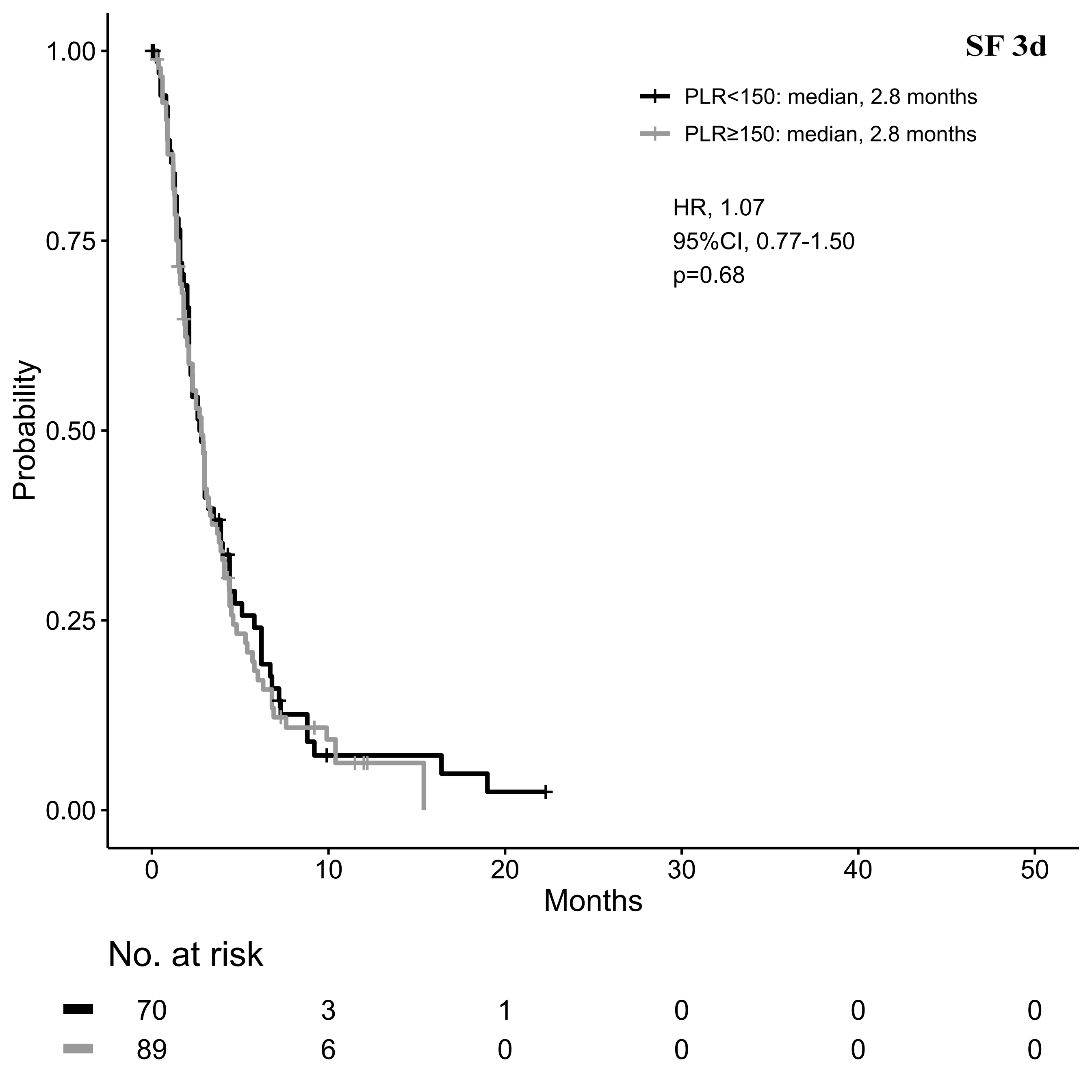

SF 3d
PLR<150: median, 2.8 months
PLR≥150: median, 2.8 months
HR, 1.07
95%CI, 0.77-1.50
p=0.68
Probability
1.00
0.75
0.50
0.25
0.00
0
10
20
30
40
50
Months
No. at risk
70 3 1 0 0 0
89 6 0 0 0 0

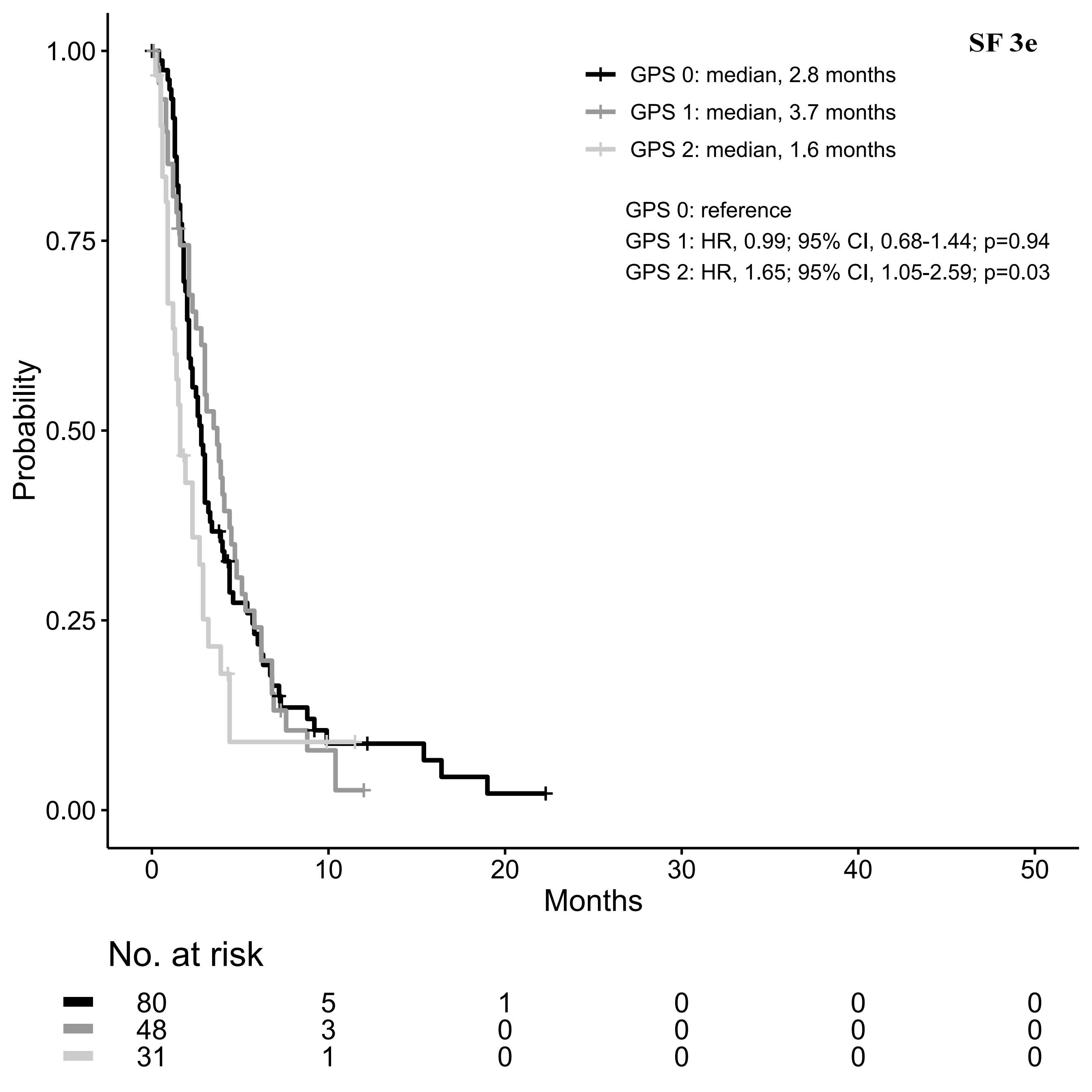
SF 3e
GPS 0: median, 2.8 months
GPS 1: median, 3.7 months
GPS 2: median, 1.6 months
GPS 0: reference
GPS 1: HR, 0.99; 95% CI, 0.68-1.44; p=0.94
GPS 2: HR, 1.65; 95% CI, 1.05-2.59; p=0.03
Probability
1.00
0.75
0.50
0.25
0.00
0
10
20
30
40
50
Months
No. at risk
80 5 1 0 0 0
48 3 0 0 0 0
31 1 0 0 0 0

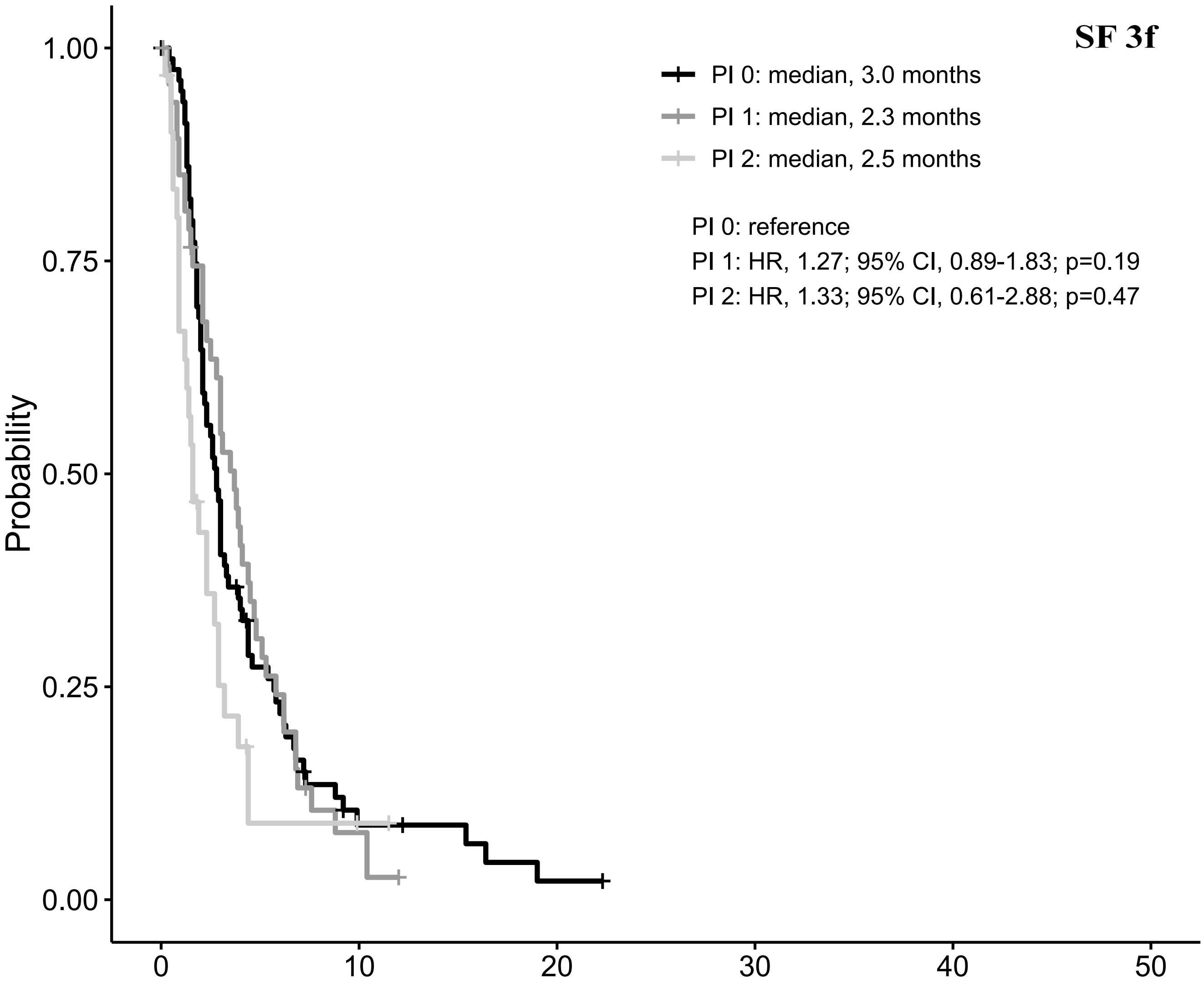

No. at risk

PI 0	80	5	1	0	0	0
PI 1	48	3	0	0	0	0
PI 2	31	1	0	0	0	0

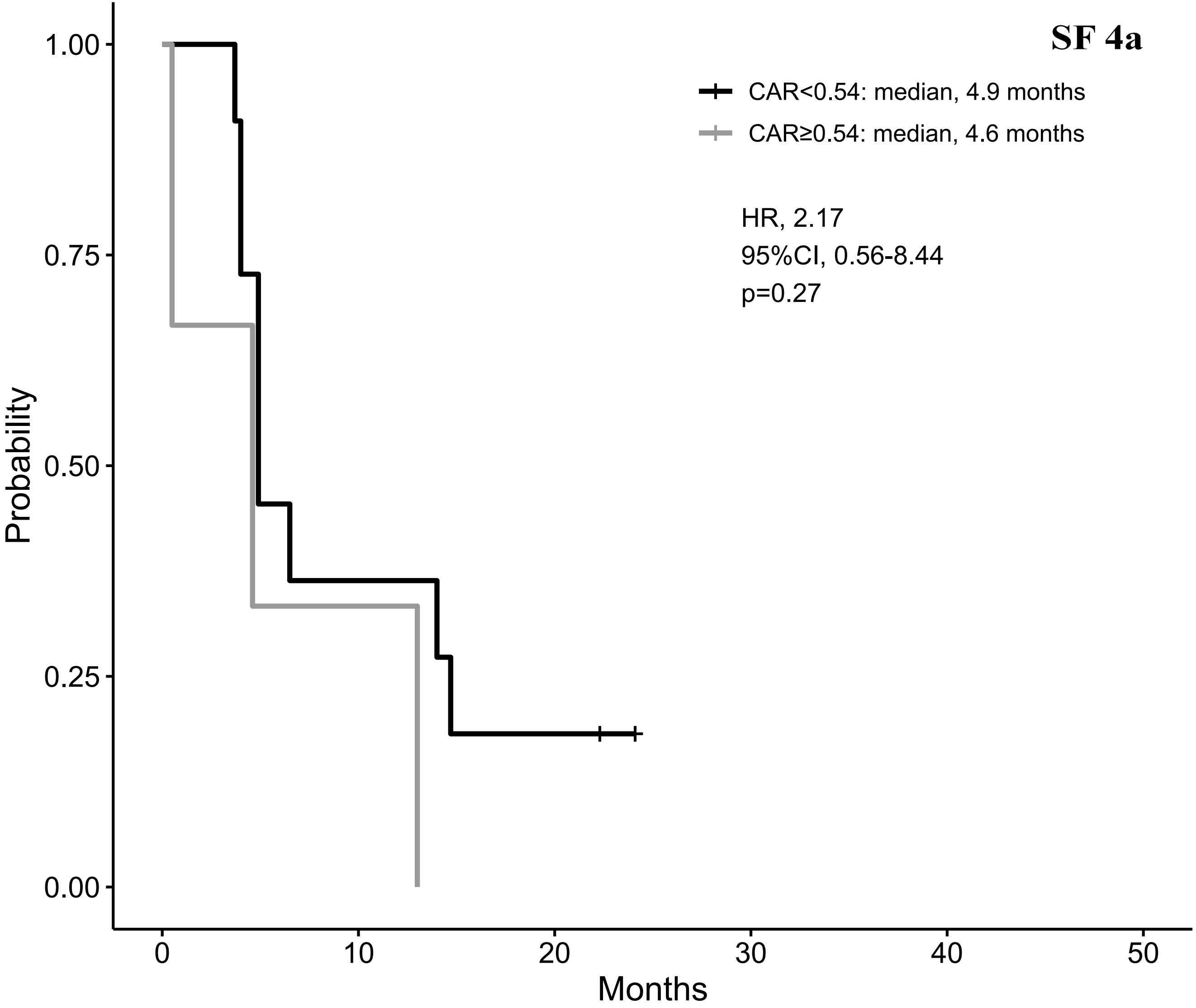

No. at risk

CAR<0.54	11	4	2	0	0	0
CAR≥0.54	3	1	0	0	0	0

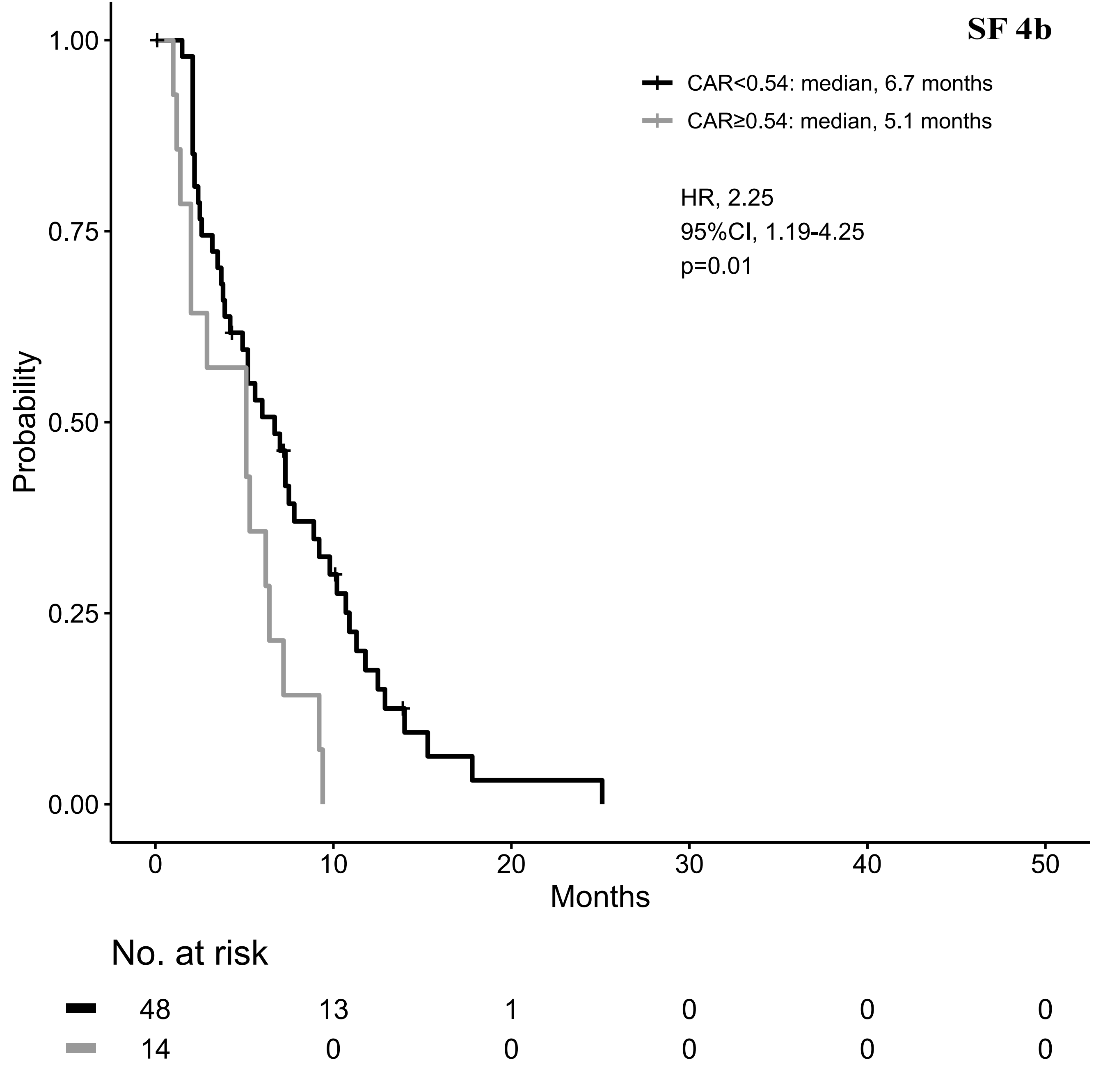
SF 4b
CAR<0.54: median, 6.7 months
CAR≥0.54: median, 5.1 months
HR, 2.25
95%CI, 1.19-4.25
p=0.01
Probability
1.00
0.75
0.50
0.25
0.00
0
10
20
30
40
50
Months
No. at risk
48
13
1
0
0
0
14
0
0
0
0
0

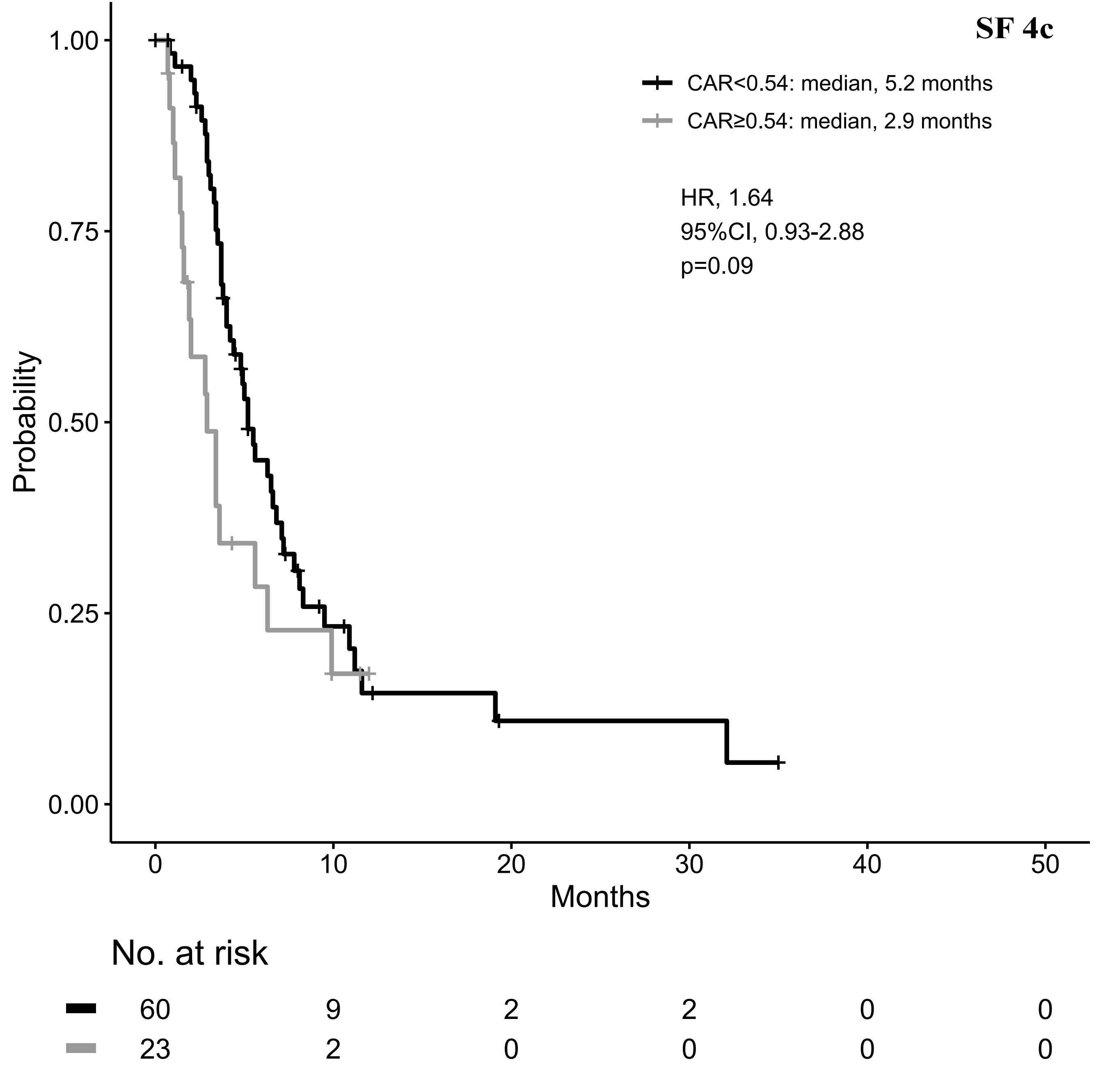
SF 4c
CAR<0.54: median, 5.2 months
CAR≥0.54: median, 2.9 months
HR, 1.64
95%CI, 0.93-2.88
p=0.09
Probability
1.00
0.75
0.50
0.25
0.00
0
10
20
30
40
50
Months
No. at risk
60 9 2 2 0 0
23 2 0 0 0 0

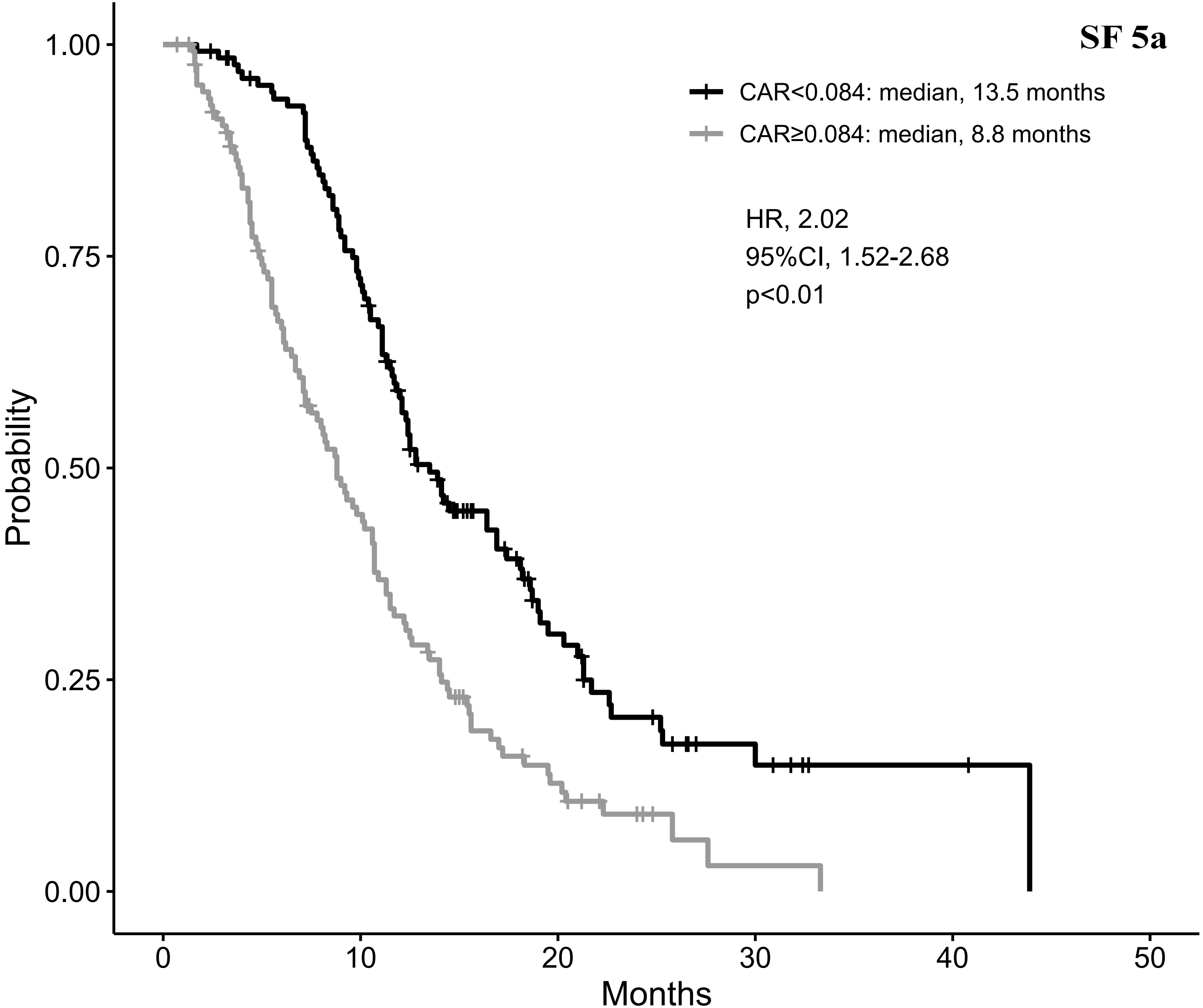

No. at risk

CAR<0.084	127	89	23	7	2	0
CAR≥0.084	128	52	12	1	0	0

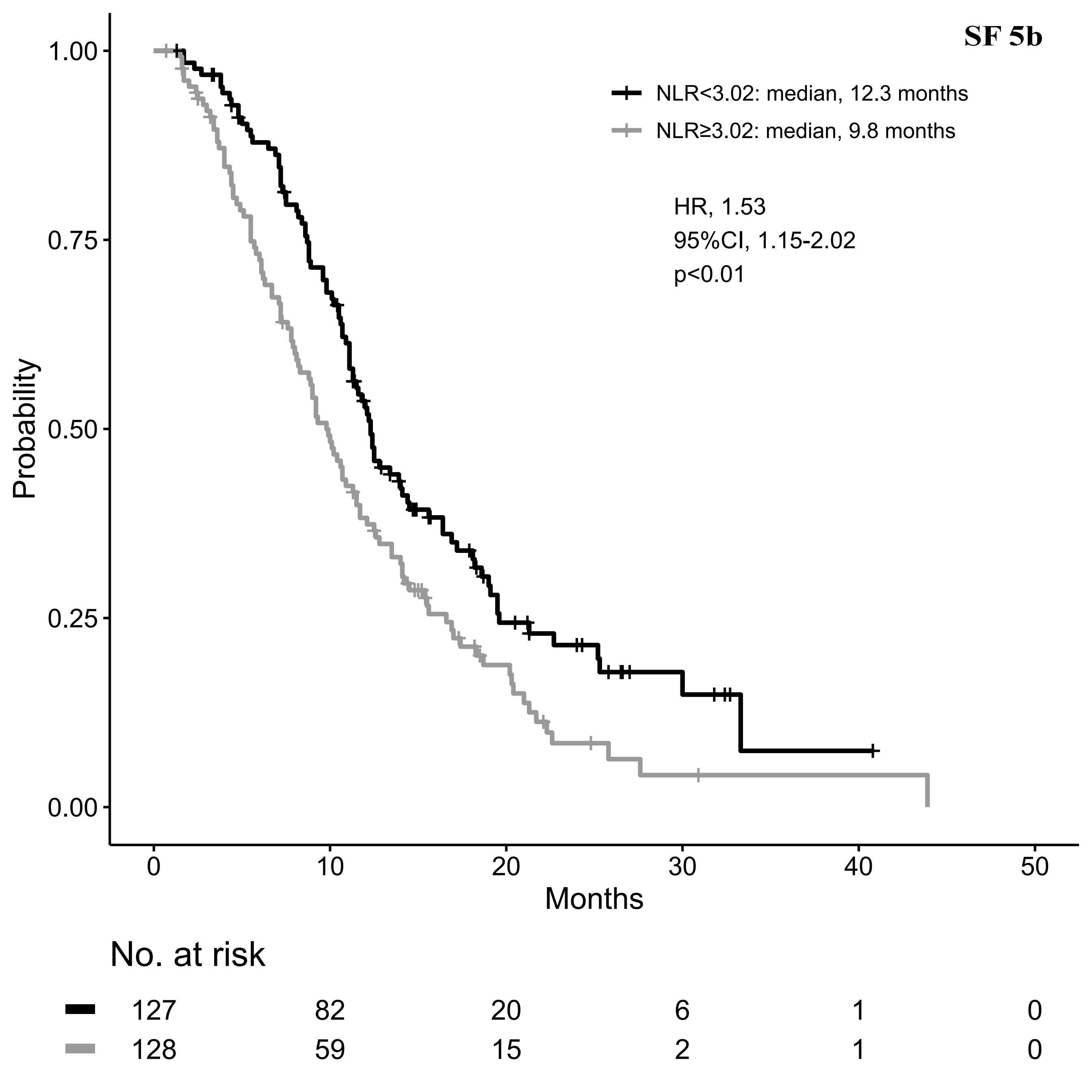
SF 5b
NLR<3.02: median, 12.3 months
NLR≥3.02: median, 9.8 months
HR, 1.53
95%CI, 1.15-2.02
p<0.01
Probability
1.00
0.75
0.50
0.25
0.00
0
10
20
30
40
50
Months
No. at risk
127 82 20 6 1 0
128 59 15 2 1 0

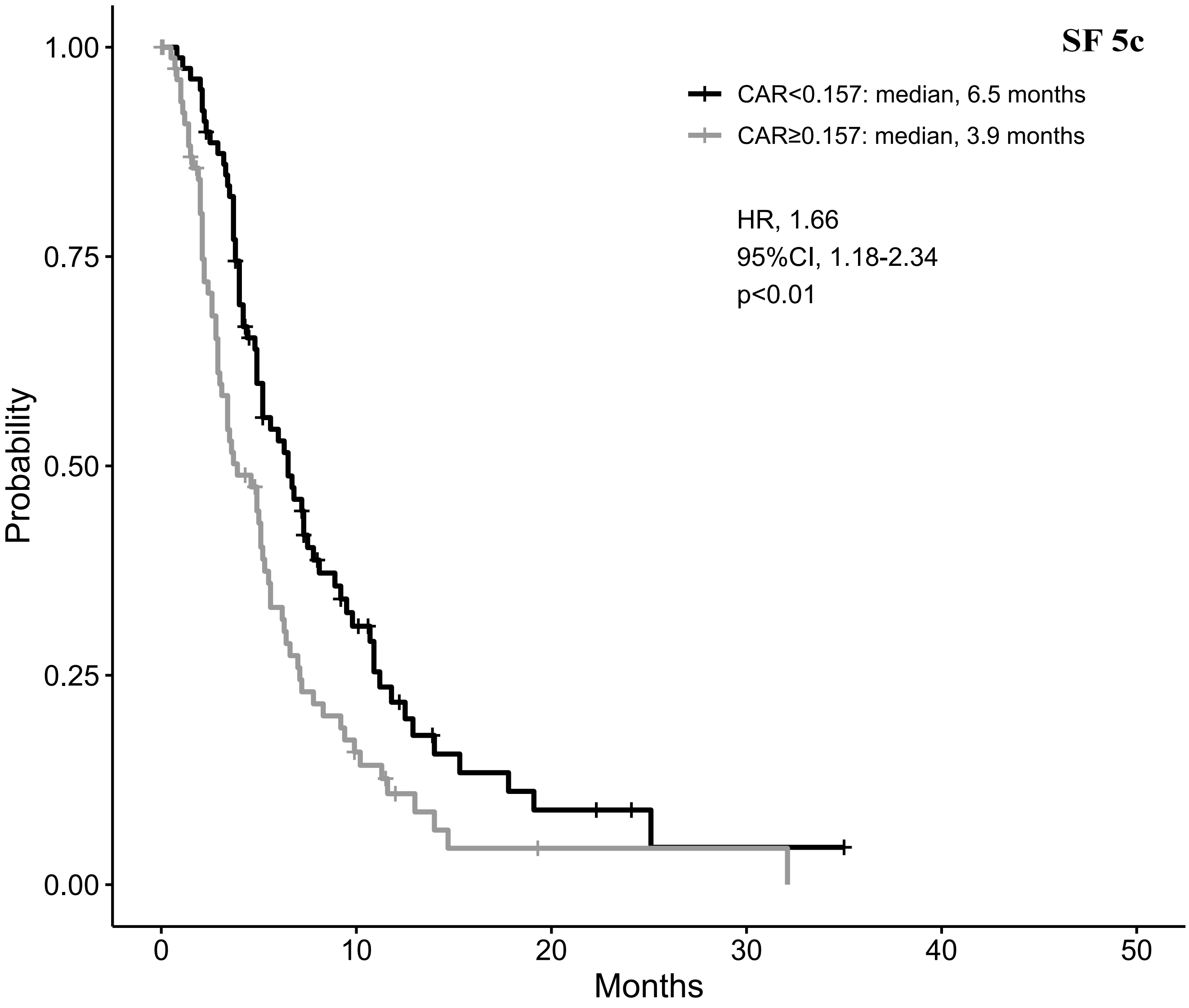

No. at risk

CAR<0.157	79	19	4	1	0	0
CAR≥0.157	80	10	1	1	0	0

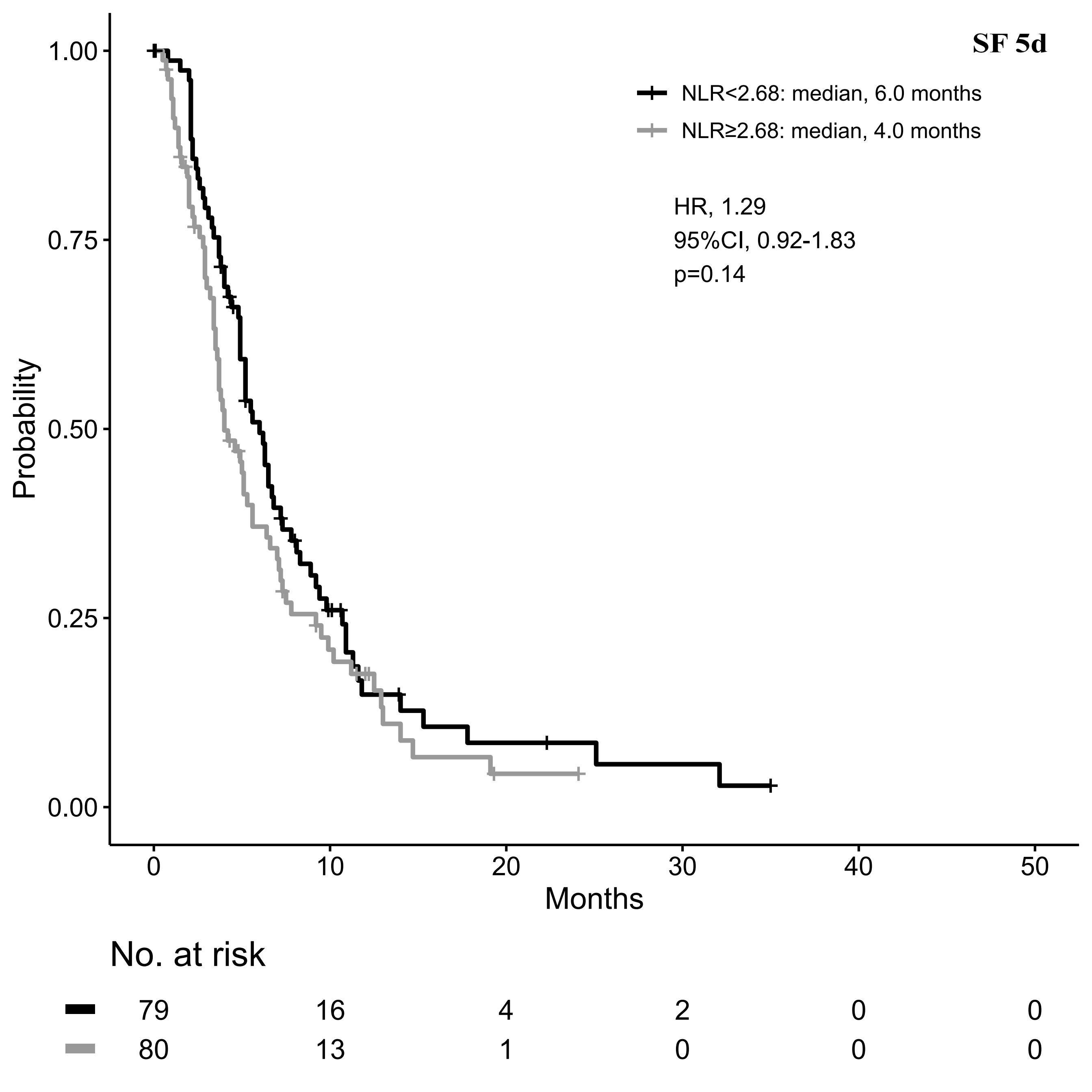
SF 5d
NLR<2.68: median, 6.0 months
NLR≥2.68: median, 4.0 months
HR, 1.29
95%CI, 0.92-1.83
p=0.14
Probability
1.00
0.75
0.50
0.25
0.00
0
10
20
30
40
50
Months
No. at risk
79 16 4 2 0 0
80 13 1 0 0 0

Supplemental Table. Baseline characteristics of the CAR < 0.54 group and CAR ≥ 0.54 group

Characteristics		Cohort 1			Cohort 2		
		CAR < 0.540	CAR ≥ 0.540	P	CAR < 0.540	CAR ≥ 0.540	P
		n=193 (%)	n=62 (%)		n=119 (%)	n=40 (%)	
Age, years	median (range)	66 (29–86)	63 (35–77)	< 0.01	65 (29–87)	62 (36–80)	0.24
	≥ 65 y.o.	110 (57)	20 (32)	< 0.01	62 (52)	16 (40)	0.19
Sex, n (%)	male	119 (62)	39 (63)	0.86	119 (62)	39 (63)	0.49
ECOG PS, n (%)	0	130 (67)	31 (50)	< 0.01	54 (45)	9 (23)	0.047
	1	56 (29)	22 (35)		52 (44)	22 (55)	
	≥ 2	7 (4)	9 (15)		13 (11)	8 (20)	
	unknown	0 (0)	0 (0)		0 (0)	1 (3)	
Malignant history, n (%)	yes	28 (15)	6 (10)	0.52	16 (13)	3 (8)	0.14
Malignant family history, n (%)	yes	59 (31)	16 (26)	0.77	41 (34)	7 (18)	0.04
Previous tumor resection, n (%)	yes	40 (21)	4 (6)	0.01	18 (15)	2 (5)	0.10
Previous biliary drainage, n (%)	yes	56 (29)	9 (15)	0.02	29 (24)	10 (25)	0.94
Pancreatic tumor location, n (%)	head	101 (52)	22 (35)	0.02	57 (48)	18 (45)	0.75
	body/tail	92 (48)	40 (65)		62 (52)	22 (55)	
Histology, n (%)	adenocarcinoma	157 (81)	55 (89)	0.35	104 (87)	34 (85)	0.91
	others	9 (5)	1 (2)		2 (2)	1 (3)	
	unknown	27 (14)	6 (10)		13 (11)	5 (13)	
Site of metastatic disease, n (%)	liver	105 (54)	49 (79)	< 0.01	73 (61)	27 (68)	0.49
	peritoneum	48 (25)	14 (23)	0.72	31 (26)	6 (15)	0.15
	lung	33 (17)	6 (10)	0.16	17 (14)	5 (13)	0.78
Number of metastatic sites, n (%)	≥ 2	66 (34)	31 (50)	0.03	46 (39)	13 (33)	0.49
Ascites, n (%)	yes	36 (19)	20 (32)	0.02	23 (19)	6 (15)	0.54
First-line chemotherapy, n (%)	FFX	76 (39)	26 (42)	0.72	60 (50)	20 (50)	0.96
	GnP	117 (61)	36 (58)		59 (50)	20 (50)	
White blood cell count, /μL	median (range)	5590 (2400–11900)	8400 (4350–23650)	< 0.01	4100 (1620–14200)	7180 (1910–21910)	<0.01
Neutrophil count, /μL	median (range)	3620 (770–8680)	5750 (2240–20810)	< 0.01	2500 (610–11720)	5260 (1230–21250)	<0.01
Lymphocyte count, /μL	median (range)	1400 (440–3710)	1170 (230–8640)	0.02	1080 (260–2630)	1100 (190–3030)	0.95
Platelet count, $\times 10^4$/μL	median (range)	19.9 (6.7–46.5)	23.3 (11.6–56.1)	< 0.01	17.6 (6.2–50.8)	20.5 (2.4–79.8)	0.07
Albumin concentration, g/dL	median (range)	4.0 (2.5–4.8)	3.34 (2.2–4.5)	< 0.01	3.7 (2.2–4.6)	3.3 (1.5–4.1)	< 0.01
CRP, mg/dL	median (range)	0.17 (0.01–2.12)	4.28 (1.73–17.0)	< 0.01	0.27 (0.01–2.00)	3.67 (2.00–16.33)	< 0.01
LDH, U/L	median (range)	174 (74–808)	203 (108–1320)	< 0.01	206 (84–713)	225 (112–447)	0.07
CEA, ng/mL	median (range)	4.7 (0.4–626.6)	13.9 (0.6–369.8)	< 0.01	7.2 (1.3–391.6)	10.8 (1.0–1379.3)	0.23
CA19-9, U/mL	median (range)	659 (1–1242609)	1821 (2–6554100)	0.12	808 (1–115905)	4111 (2–7200000)	0.02
NLR	median (range)	2.68 (0.28–8.37)	4.52 (1.25–36.88)	< 0.01	2.16 (0.37–30.00)	4.70 (1.32–49.42)	< 0.01
	≥ 5.00, n (%)	17 (9)	29 (47)	< 0.01	15 (13)	19 (48)	< 0.01
PLR	median (range)	137.3 (44.4–906.6)	209.5 (16.1–1344.8)	< 0.01	160.8 (46.6–1270.0)	178.4 (35.8–1043.1)	0.23
	≥ 150.0, n (%)	85 (44)	43 (69)	< 0.01	64 (54)	25 (63)	0.34
PNI	median (range)	46 (29–60)	39 (25–66)	< 0.01	43 (24–54)	39 (19–51)	< 0.01
	< 47, n (%)	104 (54)	53 (85)	< 0.01	95 (80)	38 (95)	0.03

CAR	median (range)	0.044 (0.002–0.523)	1.222 (0.546–7.182)	< 0.01	0.074 (0.003–0.513)	1.175 (0.562–6.387)	< 0.01
	≥ 0.540, n (%)	0 (0)	62 (100)	< 0.01	0 (0)	40 (100)	< 0.01
GPS, n (%)	0	146 (76)	0 (0)	< 0.01	80 (67)	0 (0)	< 0.01
	1	36 (19)	26 (42)		35 (29)	13 (33)	
	2	11 (6)	36 (58)		4 (3)	27 (68)	
PI, n (%)	0	165 (85)	0 (0)	< 0.01	100 (84)	0 (0)	< 0.01
	1	28 (15)	54 (87)		18 (15)	31 (78)	
	2	0 (0)	8 (13)		1 (1)	9 (23)	

Abbreviations: ECOG PS, Eastern Cooperative Oncology Group performance status; FFX, FOLFIRINOX; GnP, gemcitabine plus nab-paclitaxel; CRP, C-reactive protein; LDH, lactate dehydrogenase; CEA, carcinoembryonic antigen; CA19-9, carbohydrate antigen 19-9; NLR, neutrophil-to-lymphocyte ratio; PLR, platelet-lymphocyte ratio; PNI, prognostic nutrition index; CAR, CRP-albumin ratio; GPS, Glasgow prognostic score; PI, prognostic index.
